# A new alvarezsaurian theropod from the Upper Jurassic Shishugou Formation of western China

**DOI:** 10.1038/s41598-019-48148-7

**Published:** 2019-08-13

**Authors:** Zichuan Qin, James Clark, Jonah Choiniere, Xing Xu

**Affiliations:** 1 Key Laboratory for the Evolutionary Systematics of Vertebrates, Institute of Vertebrate, Paleontology and Paleoanthropology, Chinese Academy of Sciences, Beijing, 100044 China; 2 CAS Center of Excellence in Life and Paleoenvironment, Beijing, 100044 China; 3 University of Chinese Academy of Sciences, Beijing, 100044 China; 4 Department of Biological Sciences, George Washington University, Washington, DC USA; 5 Evolutionary Studies Institute, University of the Witwatersrand, Johannesburg, South Africa

**Keywords:** Palaeontology, Phylogenetics, Taxonomy

## Abstract

Alvarezsaurian dinosaurs, a group of bizarre theropods with greatly shortened and modified forelimbs, are known mostly from the Cretaceous of Asia and South America. Here we report a new alvarezsaurian, *Shishugounykus inexpectus* gen. et sp. nov, based on a specimen recovered from the Middle–Upper Jurassic Shishugou Formation of the Junggar Basin, western China. Together with two other alvarezsaurians from this formation, i.e., *Haplocheirus sollers* and *Aorun zhaoi*, these Shishugou forms represent the only known Jurassic alvarezsaurians worldwide. Similar to the two other Shishugou alvarezsaurians, this new alvarezsaurian displays early stages in the development of the highly modified alvarezsaurian forelimb, but it possesses a number of manual features closer to the typical coelurosaurian theropod condition. Combining morphological and histological features, our analysis indicates that the earliest known alvarezsaurians are variable in size and other important morphological features, and in particular display a mosaic distribution of forelimb features.

## Introduction

Alvarezsauria was first recognized when Bonaparte named the *Alvarezsaurus calvoi* from the Coniacian-Santonian of Argentina^1^. Since then, fossil remains of this group have been recovered from the Upper Cretaceous of North America^2,3^, South America^1,4,5^, Asia^6–15^, and Europe^16^. Many of these Upper Cretaceous taxa, such as *Mononykus*, possess highly shortened and modified forelimbs and many derived features that also occur in birds, and they were once interpreted as flightless birds^4,6,8,17–22^. The avialan affinities of late-branching alvarezsaurians were questioned by many subsequent studies^7,23,24^ and Alvarezsauria has since been placed in various positions outside Avialae in coelurosaurian phylogeny^25–32^. The discovery of *Haplocheirus sollers* from the upper part of the Shishugou Formation (Oxfordian) in Junggar Basin, Xinjiang, China, extended the fossil record of the group by nearly 70 million years and helped confirm the early-branching maniraptoran status of the group within theropods^33^. More recently, *Aorun zhaoi*, a small theropod also known from the Shishugou Formation [36], was placed within the Alvarezsauria by a phylogenetic analysis [32], adding additional diversity of the group in the Jurassic. Here we describe a new specimen recovered from the Shishugou Formation, which represents the third alvarezsaurian species in this formation, and which demonstrates significant anatomical variability previously unappreciated among the earliest known alvarezsaurians.

## Results

### Systematic palaeontology

Theropoda Marsh, 1881

Maniraptora Gauthier, 1986

Alvarezsauria Bonaparte, 1991

*Shishugounykus inexpectus* gen. et sp. nov

#### Etymology

The generic name is a combination of Shishugou (Chinese Mandarin for the formation which produced the holotype specimen of the new animal; translates as “rock” “tree” “wash” for the abundant petrified wood in the formation) and onyx (Greek, “claw); the specific name refers to the unexpected discovery of a new alvarezsaurian species from the Middle-Late Jurassic Shishugou Formation, which has produced fossils of two other Jurassic alvarezsaurians, i.e., *Haplocheirus sollers* and *Aorun zhaoi*.

#### Holotype

**Figure 1:**
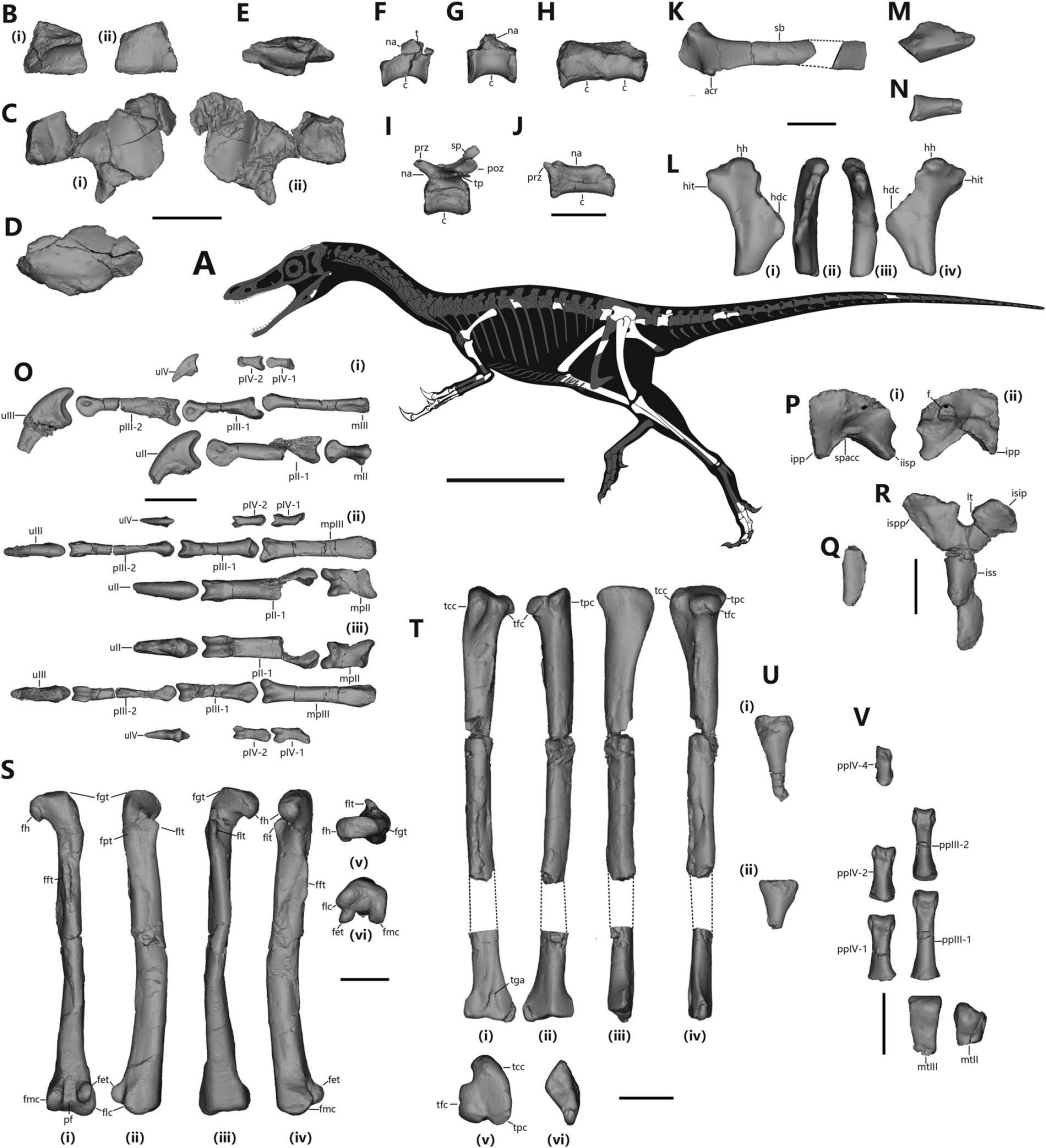
Skeletal anatomy of *Shishugounykus inexpectus* (IVPP V23567). Skeletal reconstruction showing preserved elements. (**A**), Skeletal silhouette showing preserved bones (missing portions shown in gray; Scale bar, 200 mm); (**B**), Partial left frontal in dorsal and ventral view; (**C**), Partial right frontal and parietal in dorsal and ventral view; (**D**), Partial right angular in lateral view; (**E**), Right articular in dorsal view; (**F**), An anterior dorsal in lateral view; (**G**), A posterior dorsal in lateral view; (**H**), Two most anterior sacrals in lateral view; (**I**), An anterior caudal in lateral view; (**J**), A posterior caudal in lateral view; (**K**), Right scapula in lateral view; (**L**), Partial left humerus in anterior, posterior, lateral and medial view; (**M**), Proximal end of right ulna; (**N**), Proximal end of right radius; (**O**), Right manus in lateral, dorsal and ventral view; (**P**), Partial left ilium in lateral and medial view; pubis (**Q**) and ischium (**R**) in lateral view; (**S**), Right femur in posterior, lateral, anterior and medial view; (T), Left tibia in anterior, posterior, lateral and medial view; (**U**), Left and right fibulae in lateral view; (**V**), Partial left metatarsals II and III, left pedal phalanges III-1 and 2, IV-1, 2, and 4 in dorsal view. (Figure abbreviations see supplementary materials; Scale bar, 20 mm; The skeletal silhouettes are created by Aijuan Shi using Adobe Illustrator, www.adobe.com/products/illustrator.html).

IVPP V23567, a partial skeleton (Fig. 1) including several cranial elements (possible partial right frontal and partial right parietal, partial left frontal, partial right angular, and right articular), three dorsal vertebrae, four sacral vertebrae, three caudal vertebrae, partial right scapula, partial left humerus, partial right ulna and radius, nearly complete right manus, partial left ilium, ischium, and pubis, complete right femur, partial left femur, nearly complete left and right tibiae, partial left and right fibula, a distal tarsal, partial left metatarsals II and III, left pedal phalanges III-1 and 2, IV-1, 2, and 4, and a few rib fragments and unidentifiable pieces. All recovered bones are clearly from one individual given that they are preserved in a small area (about 0.2 square meters), without any other bone nearby.

#### Locality and Horizon

Wucaiwan, Junggar Basin, Xinjiang, People’s Republic of China, Middle-Upper Jurassic Shishugou Formation^34–37^. The holotype-fossil-bearing bed is located between two volcanic tuff layers with radiometric (^40^Ar/^39^Ar) ages of 161.2 ± 0.2 and 158.7 ± 0.3 Ma, respectively^35–38^. The two tuff layers are separated by a section of fluvial sediments that is 90 meters thick, and assuming constant sedimentation rates this means that each meter of sediment is around 0.0278 million years^36^, if the sedimentation rate was relatively constant. Based on a section thickness of 36 meters between the holotype-fossil-bearing bed and the lower tuff layer, we infer that the holotype-bearing bed is ∼160.2 Ma. Using a similar method, previous studies estimate the geological ages of the fossil-bearing beds for *Aorun zhaoi* (about 13 m below the lower tuff) and *Haplocheirus sollers* (about 40 m above the lower tuff) fossils are ∼161.6 Ma and ~160.1 Ma^37^, respectively.

#### Diagnosis

*Shishugounykus inexpectus* differs from all other alvarezsaurians in having the following unique combination of features (* marks the autapomorphies; we use the II-III-IV identity of manual digits in tetanurans): supratemporal fossa occupying large portion of frontal and with indistinct anterior border (sharp anterior border in early-branching alvarezsaurians such as *Haplocheirus sollers* and supratemporal fossa occupying a small portion of frontal in late-branching alvarezsaurians); scapula with hollow acromial process but without lateral concavities*; humeral internal tuberosity mediolaterally constricted distally*, giving it a “pinched” appearance; metacarpal III straight in dorsal view (laterally bowed in most other alvarezsaurians including *Haplocheirus sollers*); ungual III-3 subequal in size to ungual II-2 (considerably smaller in most other alvarezsaurians including *Haplocheirus sollers*); iliac medial surface with step-wise transition from ischial peduncle to pubic peduncle*; distal end of metatarsal II asymmetrically ginglymoid*.

### Skeletal description and comparisons

**Figure 2:**
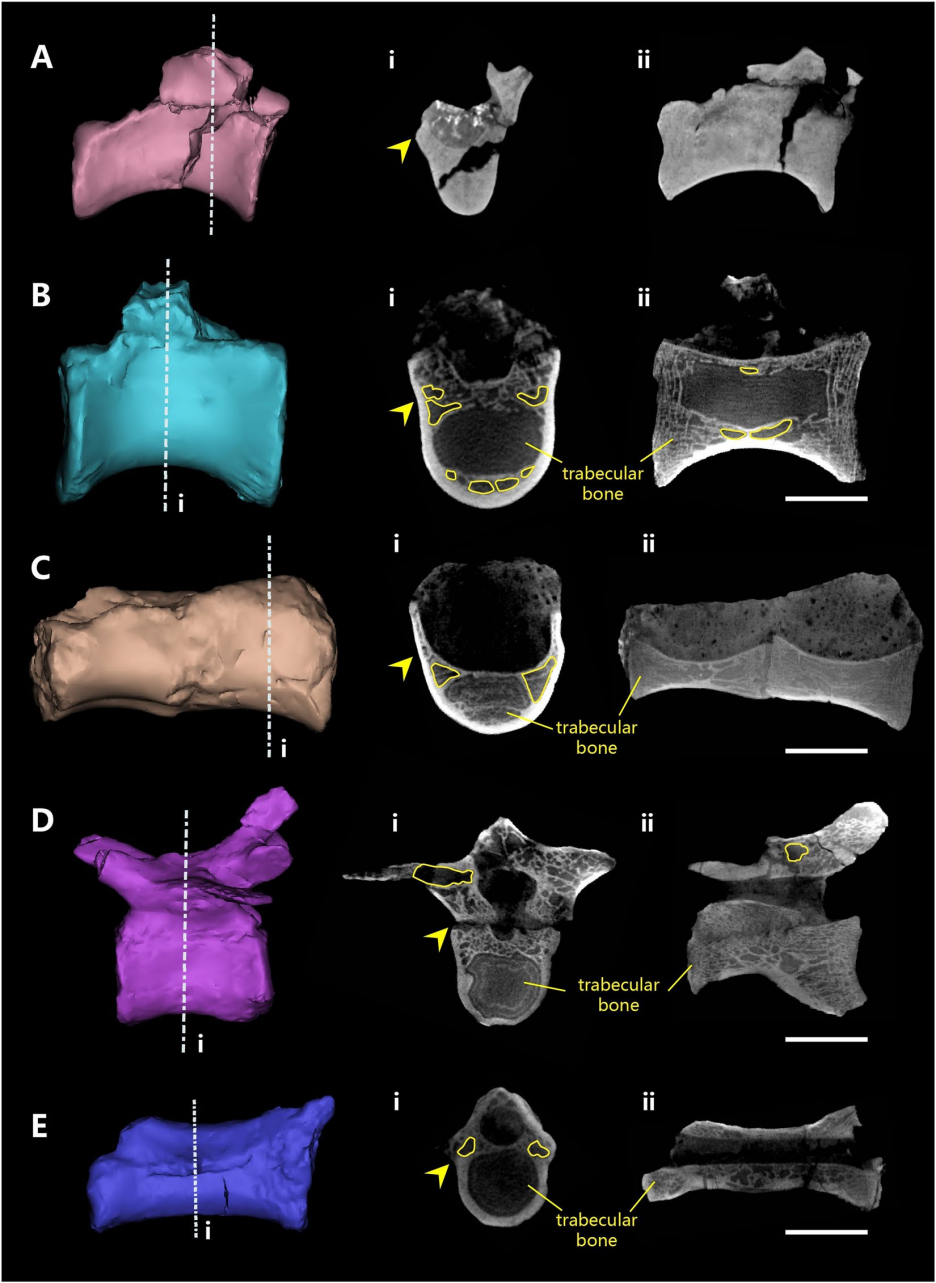
CT images of selected vertebrae of *Shishugounykus inexpectus* (IVPP V23567). (**A**), Cross section and midsagittal section of anterior dorsal vertebrae; (**B**), Cross section and midsagittal section of posterior dorsal vertebrae; (**C**), Cross section and midsagittal section of proximal sacral vertebrae; (**D**), Cross section and midsagittal section of proximal caudal vertebrae; (**E**), Cross section and midsagittal section of distal sacral vertebrae; pneumatic chambers are outlined in yellow; the sutures between centra and neural arches are marked by yellow arrows. (Scale bar, 10 mm).

The *Shishugounykus inexpectus* holotype displays three features suggesting a relatively early ontogenetic stage: the neurocentral sutures are only partially closed in one anterior caudal vertebra (Fig. 2); the fusions between the sacral centra are not complete (Fig. 2); and metacarpal II is preserved without the incorporation of the distal ‘semilunate’ carpal^39^. However, the smooth surfaces and well ossified articular ends of all preserved long bones suggest that it is not a hatchling; furthermore, the neurocentral sutures are closed in most preserved vertebrae (Fig. 2A,B,E), though many of them preserve only a small portion of the neural arch, thus suggesting a relatively late ontogenetic stage. We further investigated the ontogenetic stage of holotype individual using bone histological data (see below). Interestingly, histological features like the presence of an outside lamina of parallel-fibered tissue and abundant secondary remodeling bone tissues, suggest that the *Shishugounykus inexpectus* holotype is in a late ontogenetic stage^40,41^. Combing the evidence above, we infer this individual is an adult, close to but not at its full body size. The *Shishugounykus inexpectus* holotype is small in size, with an estimated body mass of 6.8 kg based on an empirical equation for theropod dinosaurs^42^.

The skull and mandible are represented by a partial frontal, partial laterosphenoid, partial parietal, partial angular, and articular. Most of these bones are damaged or fragmentary, and their identification was made possible with comparison to the nearly complete cranial materials from *Haplocheirus sollers* and the partial cranial material of *Xiyunykus pengi*^33,43^. However, we remain tentative about these identifications.

The frontal is represented by a partial left frontal (Fig. 1B) and a piece of bone tentatively identified as the posterior portion of the right frontal. The dorsal surface of the frontal is smooth and slightly convex. As in early-branching alvarezsaurians such as *Haplocheirus sollers* and *Xiyunykus pengi*^33^, the supratemporal fossa occupies a large portion of the frontal and has an anteriorly convex border, but unlike the former the anterior border is indistinct, a feature more similar to the condition in late-branching alvarezsaurians such as *Shuvuuia deserti*^8^. In ventral view, a strongly curved crest (the crista crania) is present, terminating close to the orbital rim. There is a depression along the posterior margin of the postorbital process of the tentatively identified right frontal, which represents the articular surface for the frontal process of the postorbital.

A piece of bone tentatively identified as the partial right parietal is represented only by partial lateral portion (Fig. 1C). In dorsal view, the posteromedial margin of the preserved parietal is slightly raised, representing the posteromedial border of the supratemporal fossa. Although both the right frontal and right parietal display some morphological features consistent with the frontal and parietal identifications, only the suspected crista crania represents an indication of the endocranial cavity, and thus they are only tentatively identified as frontal and parietal, awaiting confirmation by better preserved material.

A posteroventral portion of the right angular is preserved (Fig. 1D). The preserved portion suggests that the angular contributed to a proportionally larger portion of the posterior half of the mandible than in *Haplocheirus sollers*^33^. The lateral surface is slightly convex, similar to that of *Haplocheirus sollers*^33^. The medial surface is medially concave, forming the ventral part of the internal mandibular fossa. There is a wide, oblique groove along the posterior half of the ventral surface of the preserved angular, which forms the articular facet for the articular.

One isolated element is tentatively identified as a nearly complete right articular (Fig. 1E), which displays several unusual features. It has a sub-rectangular outline in dorsal view. The articular surface for the surangular is partially broken, but a large and deep transverse groove is present, which has not been previously reported in other alvarezsaurians. Also unusual are two features unknown in other alvarezsaurians: the anteromedial corner of the element, which represents the contact surface for the prearticular, displays a distinctive medial extension that is relatively larger than *Haplocheirus*^33^ and differs from the strap-like extension in *Shuvuuia*^19^; the glenoid portion is slightly convex rather than concave. However, some other features are similar to those in early-branching alvarezsaurians such as *Haplocheirus* and *Xiyunykus*^33,43^: there is a small notch on the lateral margin of the element in dorsal view and the retroarticular process is short and transversely broad.

Three dorsal vertebrae are preserved, and they are identified as an anterior one, a middle one, and a posterior one (the latter is likely the posteriormost one). All three dorsal centra are well preserved, but their neural arches are mostly broken. All preserved dorsals have elongate centra (much longer anteroposteriorly than dorsoventrally) and lack a pneumatic fossa on their central lateral surface as in most other alvarezsaurians except *Linhenykus monodactylus*^11,19^. Micro CT scans reveal that some internal pneumatic chambers are present bilaterally on a dorsal portion of the preserved posterior centrum (Fig. 2B), like those of *Archaeornithomimus*^44^, but without any fossa or foramina on the surface of the centrum. The middle of the centrum is occupied by trabecular bone, and pneumatic chambers are located ventrally (Fig. 2B), which are not present in those of *Archaeornithomimus*^44^.

The anterior dorsal has a transversely narrow centrum (Fig. 1F), and its posterior articular surface is slightly wider transversely than dorsoventrally. Both the anterior and posterior surfaces of the anterior dorsal centrum are concave, but with the latter distinctly larger than the former in posterior view. There is a distinct tubercle projecting laterally and posteriorly at the base of the posterior end of the neural arch, a feature appearing also in *Xiyunykus pengi* and *Bannykus wulatensis* but to a much lesser degree^43^. A posteroventrally extending ridge is identified as the posterior centrodiapophyseal lamina, but pneumatic fossae surrounding it are poorly developed, as in *Bannykus wulatensis*^43^. The centrum of the middle dorsal has slightly concave anterior and posterior articular surfaces, which are transversely wider than dorsoventrally deep. The central lateral surface is slightly depressed close to the neurocentral suture, though a pneumatic fossa is absent. The posterior dorsal is similar to the middle one in general outline except that the centrum is proportionally wider transversely (Fig. 1G). Both anterior and posterior articular surfaces of the posterior dorsal centrum are flat or even slightly convex, somewhat similar to the biconvex condition in the Cretaceous alvarezsaurians such as *Bannykus wulatensis*, *Xiyunykus pengi*, *Mononykus olecranus* and *Shuvuuia deserti*^19,43,45^.

Four sacral vertebrae are preserved. The anteriormost two sacral vertebrae are fused to each other (Fig. 1H), and both are represented by a complete centrum and a small portion of the neural arch; two separated posterior sacral vertebrae (probably the posteriormost two) are both represented by only the centra. Pleurocoels are absent in all four sacrals as in other alvarezsaurians. However, CT scans reveal bilaterally located pneumatic chambers in the middle part of centrum (Fig. 2C). The first sacral centrum is only slightly compressed dorsoventrally, with its anterior articular surface slightly wider transversely than dorsoventrally, but the remaining sacral centra are highly compressed dorsoventrally (e.g., the anterior articular surface of the third sacral centrum is about twice as wide transversely as dorsoventrally deep). The first sacral centrum bears a slightly concave anterior articular surface, somewhat similar to the condition in Cretaceous alvarezsaurians^17,22,33^. The ventral surfaces of the first and last sacral centrum are broadly convex, but those of the middle two sacral centra bear a shallow sulcus, a feature common in theropods, but unlike the keeled ventral surfaces in many Cretaceous alvarezsaurians including *Xiyunykus pengi*^43^, *Shuvuuia deserti*^19^, and *Mononykus olecranus*^17^.

Three separate caudal vertebrae are preserved, and they are identified as an anterior one, an anteromedian one, and a middle one. All preserved caudal centra are amphicoelous, lack pneumatic fossae, and bear no longitudinal ventral sulcus.

The anterior caudal is nearly complete (Fig. 1I), and its centrum and neural arch are firmly sutured together, but with the suture visible. The centrum of the anterior caudal is longer anteroposteriorly than dorsoventrally in lateral view and has a rounded ventral surface. The prezygapophyses extend anterodorsally considerably beyond the anterior central articular surface, with the articular surfaces dorsomedially oriented. A deep infra-spinal fossa is present at the base of the anterior margin of the neural spine between the prezygapophyses. The postzygapophyses extend posteriorly slightly beyond the level of the posterior central articular surface. The transverse processes have a centrally positioned, anteroposteriorly broad base and are much longer mediolaterally than anteroposteriorly wide. There are pneumatic chambers located close to the base of transverse processes (Fig. 2D), like those of *Archaeornithomimus*^44^. They extend posterolaterally, with the distal end about the level of the posterior central articular surface. There is a tall caudal neural spine located immediately above the postzygapophyses. The blade-like neural spine is anteroposteriorly narrow in lateral view and posterodorsally oriented. This spine is different from those of *Haplocheirus sollers*, in which the neural spines have a wide base and are oriented nearly dorsally^33^.

The preserved antero-middle caudal is represented by an incomplete centrum. It is shallower in lateral view and slightly more compressed transversely than the anterior caudal centrum. The middle caudal is nearly complete, missing postzygapophyses and parts of neural spine and transverse processes. The middle caudal (Fig. 1J) is much longer than the anterior caudal (about 1.5 times as long as the latter), a feature also known in some South American alvarezsaurians such as *Alvarezsaurus*^1^. The centrum is about 2.5 times as long anteroposteriorly as dorsoventrally deep in lateral view, and transverse width and dorsoventral depth of the anterior and posterior articular surfaces are about the same. Also similar to some South American alvarezsaurians, the neural arch is positioned anteriorly, the prezygapophyses are short (projecting slightly beyond the anterior central articular end), and the transverse processes are positioned posteriorly. CT scans reveals two bilateral pneumatic chambers, slightly ventral to the spinal foramen (Fig. 2E), which are nonexistent in *Archaeornithomimus*^44^. The neural spine is a low ridge, and its posterior portion is inferred to be more prominent than the anterior portion.

A middle chevron is mostly preserved, missing both anterior and posterior extremities. It is inferred to be shaped somewhat like an inverted T lateral view. It is strongly expanded anteroposteriorly distally to form an anterior and a posterior process, with the anterior one transversely thinner and probably anteroposteriorly shorter than the latter.

The right scapula is preserved (Fig. 1K), missing much of the acromial process and the distal portion. The scapula is in general similar to that of *Haplocheirus sollers*^33^: the acromial process is large and expands anteriorly and medially from the scapula blade, a large fossa is present on the scapular lateral surface close to the proximal end, the glenoid process is short and projects posteriorly nearly perpendicular to the scapular blade, a small lip is present dorsal to the glenoid fossa, the proximal portion of the anterior margin of scapular blade is sharp and the proximal half of the posterior margin of the scapular blade is robust, and the scapular blade is strongly curved medially and is distally expanded. However, there are some differences in scapular morphology between the two taxa. Compared to *Haplocheirus sollers*^33^, the acromial process is longer proximodistally and much thicker transversely, the fossa is relatively shallower, the articular surface for the coracoid is proportionally much wider transversely, and the proximal half of the posterior margin of the scapular blade is proportionally thicker transversely. Most unusually, the acromial process is hollow inside.

The left humerus is preserved (Fig. 1L), missing part of the deltopectoral crest and the distal portion. The humeral head is relatively small, and it expands proximally and posteriorly, but not anteriorly. The internal tuberosity is large and proximodistally long. Unlike some late-branching alvarezsaurians such as *Mononykus olecranus* and *Shuvuuia deserti*^19^, it is relatively thin anteroposteriorly rather than strongly expanded, is lower than the humeral head, and the separation from the latter is minimal. *Haplocheirus sollers* possesses a deep notch between the internal tuberosity and the humeral head as in some late-branching alvarezsaurians^33^ and late-branching therizinosaurs^46^, but this might be a preservational artefact. A shallow notch is present on the medial margin of the internal tuberosity in anterior or posterior view, a feature appearing to be also present in *Xiyunykus pengi*. In medial view, the internal tuberosity is pinched distally, while in other alvarezsaurians it is consistent in width along the length. There is an obliquely extended elongate tubercle on the posterior surface of the internal tuberosity, which is also seen in *Bannykus wulatensis*^43^. The deltopectoral crest is mostly broken, but it is inferred to be large and extends anterolaterally rather than anteriorly as in late-branching alvarezsaurians. A shallow groove runs distally immediately distal to the deltopectoral crest, a feature also seen in *Xiyunykus pengi* and some other maniraptorans, but it is longer and located more distally than in other taxa^43^. The diaphysis displays a sub-triangular cross section where it is broken about 15 mm distal to the deltopectoral crest. An isolated bone fragment is probably a portion of the humeral shaft, and the rounded cross section shows the bone is extremely thin-walled (about 1.5 mm in thickness compared to a 10-mm-diameter for the cross section of the bone).

The ulna (Fig. 1M) is represented only by the partial proximal end of the right ulna. The olecranon process is mostly missing, but it is inferred to be large. As in most theropods, the olecranon process is in line with the coronoid process and is sub-triangular in cross section, unlike the anteroposteriorly compressed and medially extended one in Cretaceous alvarezsaurians. The coronoid process is relatively small, and there is a large articular facet for the humerus on the coronoid process, which extends proximally onto the olecranon process. The lateral flange is small but robust, and bears a flat articular facet for the humerus. There is a longitudinal ridge along the lateral surface extending distally.

The radius (Fig. 1N) is represented only by a partial proximal portion of the right radius. The radius is much thinner than the ulna. The proximal end has an elongate oval shape in proximal view and bears a deep fossa representing the articular facet for the humerus.

The right manus is mostly preserved (Fig. 1O), missing all carpals, metacarpal IV, and phalanx IV-3. Over all the hand has a robust digit II and two slender lateral digits. Digit III is markedly longer than digits II and IV, and it is much slenderer than digit II.

Metacarpal II is short and stout, but proportionally more slender than that of *Haplocheirus sollers* (proximodistal length/transverse width ratio 1.64, compared to 1.26 in *Haplocheirus sollers*^33^). As in other alvarezsaurians, the proximal articular surface is sub-L-shaped due to the lack of a ventral extension of the proximolateral corner, rather than sub-triradiate due to a prominent ventral extension as in most other tetanuran theropods, and the lateral portion of the proximal articular surface is slightly inclined distally. As in most tetanuran theropods, a proximomedial process is present, and it is proportionally smaller than in *Haplocheirus sollers*^33^. The proximomedial process is defined medially by a depression on the ventral surface. The dorsal surface of metacarpal II is shallowly concave and the ventral surface is broad and flat. There is a distinct dorsal projection at the proximolateral corner of the bone, the lateral surface of which contributes to part of the articular facet for metacarpal III. The articular facet for metacarpal III is slightly concave and occupies more than the proximal half of the lateral surface of metacarpal II. The distal end is strongly ginglymoid, with two hemicondyles separated by a deep groove. The hemicondyles are asymmetrical, with the lateral condyle extending distally far more than the medial one. The collateral ligament pits are nearly absent, though the medial surface of the distal end is slightly concave.

Metacarpal III is much thinner than metacarpal II. It is straight in dorsal view, unlike the laterally bowed one in most other alvarezsaurians including *Haplocheirus sollers* and *Aorun zhaoi*^33,37^. Similar to Cretaceous alvarezsaurians but unlike *Haplocheirus sollers* and *Aorun zhaoi*^33,37^, metacarpal III is flattened dorsoventrally, with a transverse width greater than its dorsoventral depth. The dorsal surface is flat and lacks a longitudinal thick ridge along the medial edge of the proximal portion of the shaft as in some alvarezsaurians such as *Haplocheirus sollers* and *Bannykus wulatensis*^33,43^. As in *Bannykus wulatensis*^43^, there is a lateral flange overhanging the lateral surface of the proximal one-fourth of the shaft, together with the lateral surface forming the articular facet for metacarpal IV, while *Haplocheirus sollers* lacks such a lateral flange^33^. The ventral surface of the proximal portion is slightly concave and that of the remaining shaft is slightly convex. There is a slightly curved, longitudinal ridge along the lateral edge of the ventral surface, emanating from the proximal end, and terminating at the level of the proximal two-thirds of the shaft. There is a shallow extensor pit on the dorsal surface near the distal end, while it is absent in *Aorun zhaoi* and *Haplocheirus sollers*^37^. The distal end of metacarpal III is ginglymoid with a well-developed trochlea extending onto the dorsal surface of the phalanx, and the lateral hemicondyle is larger than the medial one.

As in other alvarezsaurians, phalanx II-1 is the most robust element, with a shaft diameter much greater than any other phalanx. Phalanx II-1 is longer than metacarpal III and phalanx III-2, while the opposite is true in *Haplocheirus sollers*^33^. It is straight in medial or lateral views, unlike the ventrally curved one in many other alvarezsaurians including *Haplocheirus sollers* and *Aorun zhaoi*^33,37^. The ventral surface of the middle portion is slightly concave, but a fully developed ventral furrow as in many other alvarezsaurians including *Haplocheirus sollers* is absent^33^. However, a flexor pit is present immediately proximal to the distal end. The dorsal surface of phalanx II-1 is transversely convex, the medial surface is slightly inclined laterally, and the lateral surface is vertical, while in *Haplocheirus sollers* and *Aorun zhaoi*^33^, the shaft has a sub-triangular cross section, with both the medial and lateral surfaces inclined toward the central. The distal end is ginglymoid with more dorsally displaced collateral ligament than in *Haplocheirus sollers* but similar to the condition in *Aorun zhaoi*^33^. In medial or lateral views, the hemicondyles are more elongated proximodistally than in *Haplocheirus sollers*.

Phalanx III-1 is a dorsoventrally flattened element, with a relatively greater transverse width, while the opposite is true for *Haplocheirus sollers* and *Aorun zhaoi*^33,37^. It has an asymmetrical proximal articular surface with a mediolaterally narrow medial cotyle and a wide lateral cotyle. The proximal end is expanded both transversely and ventrally, and bears a proximoventral heel which is particularly prominent along the medial side. The ventral surface is flat and distally displays a well-developed flexor fossa. The distal end is strongly ginglymoid and the collateral ligament pits are located centrally.

Phalanx III-2 is subequal in length to metacarpal III, similar to the condition in *Aorun zhaoi* but unlike in *Haplocheirus sollers* where phalanx III-2 is distinctly shorter than metacarpal III^33^. The proximal articular surface is mediolaterally narrow and sub-triangular in outline. The proximal half of the shaft is highly compressed transversely as in *Aorun zhaoi*^37^, though in the latter, the whole shaft is strongly compressed. The distal end is strongly ginglymoid with a relatively wider trochlea between the hemicondyles, compared to the compressed distal end and narrow trochlea of *Aorun zhaoi*. Deep collateral ligament pits are dorsally displaced.

Phalanges IV-1 and IV-2 are similar in size. Both are mediolaterally narrow, unlike the relatively robust ones in *Haplocheirus sollers*^33^. Phalanx IV-1 is relatively slender, with a ratio of proximodistal length/mid-shaft width 4.1, compared to 3.2 in *Haplocheirus sollers*. The distal end is strongly ginglymoid with shallow ligament pits, while in *Haplocheirus sollers* they are absent^33^. Phalanx III-2 has a large proximoventral heel, which is more prominent along the medial side. The distal end bears shallow ligament pits.

Ungual II is relatively small, estimated to be shorter in length than phalanx II-1 as in *Aorun zhaoi*^37^, whereas in *Haplocheirus sollers*^33^ it is considerably longer than phalanx II-2. It is curved ventrally, to a degree intermediate between *Haplocheirus sollers* and *Aorun zhaoi*^33,37^. The proximal articular surface is about twice as tall dorsoventrally as wide mediolaterally, and is a dorsoventrally tall ellipse in outline, unlike in Cretaceous alvarezsaurians where the proximal surface is proportionally lower dorsoventrally and has sub-rectangular outline^17,19^. The flexor tubercle is slightly distally located, and is relatively small and somewhat compressed transversely, unlike the transversely expanded one in *Haplocheirus sollers*. The ungual is laterally compressed, with a sub-oval cross section.

Ungual III is subequal in size to ungual II, proportionally much larger than that of other alvarezsaurians including like *Haplocheirus sollers* and *Aorun zhaoi*^33,37^. It curves ventrally much more than that of *Haplocheirus sollers* and *Aorun zhaoi*^33,37^. The flexor tubercle is only slightly distally displaced. The cross section of ungual III is sub-triangular, with a transversely flat ventral surface which inclines medially. The medial collateral groove is positioned higher than the lateral one.

Ungual IV is significantly smaller than the other two unguals, with the proximal end about half the height of that of ungual III. It is relatively robust, with a length/depth ratio of about 2.0 compared to 3.3 in *Haplocheirus sollers*^33,37^. It curves ventrally much more so than in *Haplocheirus sollers* and *Aorun zhaoi*. The flexor tubercle is only slightly displaced distally, small in size, and transversely narrow, unlike the distally located and transversely expanded one in *Haplocheirus sollers*^33^.

The ilium is represented only by the acetabular region of the left ilium (Fig. 1P). The pubic peduncle is mostly broken, but it is inferred to be level with the iliac blade rather than medially projected. The supraacetabular crest expands laterally near the center of the acetabulum but more anteriorly it inclines ventrally to form a hood, unlike the supraacetabular crest of *Bannykus wulatensis* which projects laterally along nearly whole length of the acetabulum^43^. These features are closer in condition to Late Cretaceous parvicursorine alvarezsaurians such as *Xixianykus*^10^. The ischial peduncle is relatively small and well developed along the transverse axis showing an oblate articular facet for the ischium. This transverse widening may represent the initial development of the highly modified ischial peduncle of Late Cretaceous alvarezsaurians^17^. In medial view, the acetabular opening is subtriangular in outline, as in other alvarezsaurians (e.g., *Mononykus olecranus*, *Patagonykus puertai*)^4,17^. There are several unusual features in the ilium: the medial surface of the ischial peduncle is flat, and with a step-wise transition to that of the pubic peduncle which is strongly convex; a deep and round fossa is positioned anterodorsal to the ischial peduncle on the medial surface of iliac blade. These features might be autapomorphies of *Shishugounykus inexpectus* if they are not preservational artefacts.

Only a small piece of the left pubis is preserved (Fig. 1Q). The shaft is considerably narrower than the ischial shaft in lateral view, with a longitudinal oblique groove running along the lateral surface of the preserved shaft. The pubic apron has a slightly convex anterior surface and a flat posterior surface, and it emanates from the anterior surface of the pubic shaft.

A proximal portion of the left ischium is preserved (Fig. 1R). The pubic peduncle is large and at an angle of about 110 degrees to the pubic shaft. The proximal articular facet for the pubis is sub-semi-circular in outline, which is transversely much narrower anteriorly than posteriorly. The iliac peduncle is slightly slenderer than the pubic peduncle in lateral view but is transversely wider than the latter. The articular facet for the ilium is sub-rectangular. There is a lateral tubercle at the anterolateral corner of the iliac peduncle. An obturator process is absent in the preserved portion of the ischium, suggesting that it is either absent as in Late Cretaceous alvarezsaurians or more distally positioned than in *Haplocheirus*. The ischial shaft is rod-like but much wider anteroposteriorly than transversely. The lateral surface of the ischial shaft is flat, and the medial surface is convex. There is a longitudinal groove along the posterior surface of the ischium, a feature also reported in *Xixianykus zhangi*^10^.

The femur is represented by the nearly complete right femur (Fig. S1) and most of the left one, which is missing the proximal end. The femoral head is oriented medially and is slightly lower than the greater trochanter, without a constricted neck from the latter. Its articular surface is extensive, with a relatively large distal component, which is different from Late Cretaceous alvarezsaurians such as *Xixianykus*^10^. There is an oblique ligament groove on the posterior surface of the femoral head, but its lateral border is indistinct. The greater trochanter is narrower anteroposteriorly than the femoral head and is oriented anteromedially rather than anteroposteriorly. An anteroposteriorly narrow trochanteric shelf is present on the femoral lateral surface immediately below the greater trochanter. The lesser trochanter, which is separated from the great trochanter by a deep notch, is proximally broken, but it is inferred to be wing-like structure and to lower than the greater trochanter, as in early-branching alvarezsaurians including *Bannykus wulatensis*^43^. The bump-like posterolateral trochanter is present distal to the trochanter shelf on the femoral lateral surface, separated from the lesser trochanter by a shallow groove. The fourth trochanter is present as a thick ridge, emanating about 35 mm distal to the proximal end of the femur and running distally for 25 mm along the posteromedial margin. There is a shallow groove immediately lateral to the fourth trochanter on the femoral posterior surface. The femoral shaft is slender and slightly bowed anteroposteriorly. Distally, the anterior surface is smooth, without an anterior longitudinal groove like in *Patagonykus puertai*^4^. The posterior surface is transversely concave, bears a shallow, triangular depression (popliteal fossa) bounded by the lateral and medial ridges emanating from the lateral and medial distal condyles, respectively. The popliteal fossa opens distally as in *Patagonykus puertai* and *Alvarezsaurus calvoi*^1,4^ but differs from the distally closed ones in *Mononykus olecranus* and *Shuvuuia deserti*^8,17,22^. The medial condyle is rectangular in distal view and is anteroposteriorly convex. The ectocondylar tuber is sub-hemispherical, with the media surface convex and lateral surface flat. The lateral surface of the ectocondylar tuber also appears to be flat in some late-branching alvarezsaurians such as *Mononykus olecranus* and *Xixianykus zhangi*^10,17^. The tuber is proximodistally elongate and transversely narrow, and is distally separated from the lateral condyle by a shallow notch. The lateral condyle is cone-shaped and extends further distally than the medial condyle as in all known alvarezsaurians^19^.

A nearly complete left tibia (Fig. 1T) and the proximal and distal portions of right tibia are preserved. The proximal articular surface of the tibia is almost flat and slightly inclined laterally, and it has a semicircular anteromedial margin, like that of *Patagonykus puertai*^4^. In proximal view, the cnemial crest is prominent like that of *Patagonykus puertai*^4^, and differs from the robust and proximodistally short cnemial crest of Late Cretaceous alvarezsaurians such as *Mononykus olecranus*^17^. Like *Patagonykus puertai*^4^, it projects proximally only to the level of the proximal part of the fibular (lateral) and posterior condyle, rather than beyond them as in some theropods^47–49^. In anterior view, the cnemial crest is cylindrical, and it gradually narrows as a ridge on the anterior surface of the tibial shaft distally. A short, thick ridge is present on the lateral surface of the cnemial crest, which is absent or weak in early-branching alvarezsaurians including *Xiyunykus pengi* and *Patagonykus puertai*^4,43^. There is no accessory medial crest as in *Mononykus olecranus*^17^. The cnemial crest is separated from the fibular (lateral) condyle by a smooth and wide notch. Overall, the fibular condyle resembles those of early-branching alvarezsaurians including *Xiyunykus pengi* and *Patagonykus puertai*^4,43^, which is composed of a comparatively small anterior prominence and a bulky posterior prominence. These two prominences are separated by a shallow depression. Comparatively, the anterior prominence of *Shishugounykus* is intumescent and orbicular rather than weak and pyramidal like the anterior prominence in *Xiyunykus pengi* and *Patagonykus puertai*^4,43^. The fibular and posterior condyles are separated by a narrow notch, which is shallow and wide compared to *Xiyunykus pengi* and *Patagonykus puertai*^4,43^. The posterior condyle is triangular in proximal view, and its surface inclines anterodistally, but is not as oblique as that of *Xiyunykus pengi*^43^. The fibular crest is relatively prominent and is located about 10 mm distally from the fibular condyle, as in *Xiyunykus pengi* and *Patagonykus puertai*^4,43^, but unlike the fibular crest connecting to the proximal end in *Haplocheirus sollers*^33^. The shaft is straight and circular in cross section. The distal end is mediolaterally much broader than anteroposteriorly narrow, with the medial margin much thicker than the lateral margin in distal view. There is a distally tapered, relatively long groove on the anterior surface of the distal portion of the tibia, representing the contact surface for the ascending process of the astragalus. The groove is deeper and mediolaterally broader than that in *Aorun zhaoi*. Further, this groove is slightly oblique from mediodistal to lateroproximal, which is different from the almost proximodistally oriented grooves in *Aorun zhaoi* and *Tugulusaurus faciles*^37,50^.

The fibulae are represented by the proximal end of both right and left bones (Fig. 1U). The proximal end is crescentic in outline in proximal view and it is nearly symmetrical in lateral view, unlike the asymmetrical condition in *Haplocheirus sollers* and *Patagonykus puertai*^4,33^, but close to the condition in *Xiyunykus pengi*^43^. There is a shallow fossa on the medial surface immediately distal to the proximal end, from which is a shallow groove extends distally along the shaft.

A lamelliform distal tarsal is preserved, implying no fusion between it and the proximal metatarsals. Only the distal portion of left metatarsal II and III are preserved (Fig. 1V). The distal end of metatarsal III is symmetrical while the slightly ginglymoid one of metatarsal II is asymmetrical, with the lateral hemicondyle much larger than the medial one. Both metatarsals bear distinct collateral ligament pits on both lateral and medial surfaces of the distal end.

The left pedal phalanges III-1 and 2, IV-1, 2, and 4 are well preserved (Fig. 1V). Phalanx III-1 is the longest of all the preserved phalanges, with a slender and circular-cross-sectioned shaft. The proximal articular surface is concave, and the distal end is slightly ginglymoid. A well-developed extensor ligament pit immediately proximal to the distal condyle is nearly as wide as the shaft. Phalanx III-2 is similar in general morphology to III-1, but is shorter (about 80% of the latter in length) and has a deeper ginglymus distally. Phalanges IV-1 and 2 are stouter than those of digits III, and their extensor ligament pits are shallower than those of digit III. Phalanx IV-4 is short and its distal end is asymmetric where the lateral condyle is more developed than the medial one.

### Histological description and comparisons

**Figure 3:**
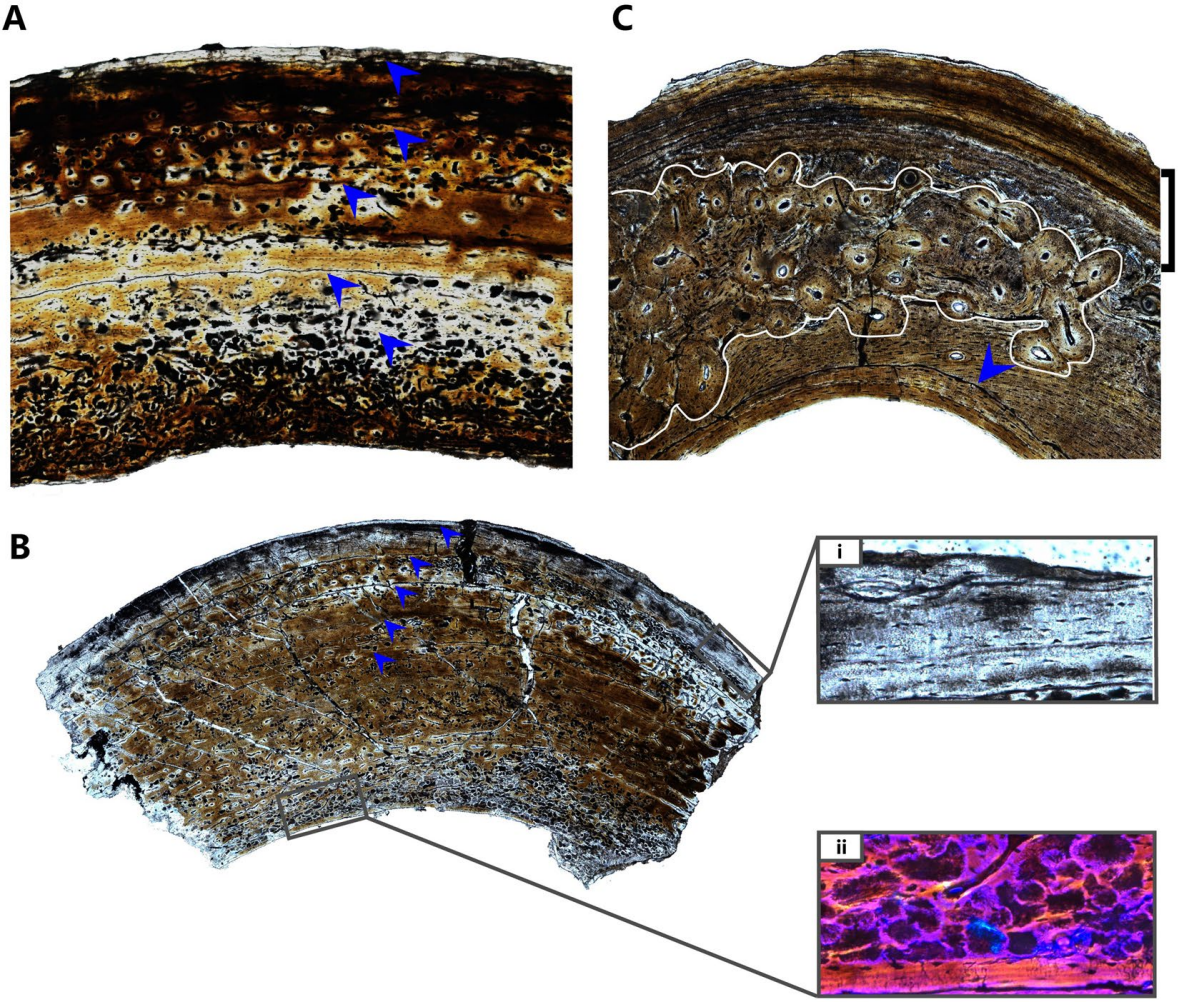
Bone histology of *Shishugounykus inexpectus* (IVPP V23567). Photographs of transverse thin section of the femoral (**A**), tibial (**B**) and fibular (**C**) mid-shaft of *Shishugounykus inexpectus* IVPP V 23567 under normal and polarized light; Blue arrows indicate growth marks in (**A**); Blue arrows indicate growth marks in (**B**), (i) and (ii) show the outermost avascular parallel-fibered tissue in normal light and innermost endosteal lamellae in polarized scope, respectively; In (**C**), the blue arrow indicates the doublet endosteal lamellae, the secondary osteons are marked by a white line, and the EFS structure is indicated by a black bracket.

The cortex of the sampled femoral section is about 1.6 mm thick, compared to a femoral diameter of 16.1 mm at the sampled region (Fig. 3A). The cortex is mostly composed of fibrolamellar tissues like most of published sections of alvarezsaurians, but differs from the woven-tissue dominated condition in *Aorun zhaoi*^37^. There is a lamina of parallel-fibered tissue at the outermost of the section, possibly representing an EFS structure. Most of the osteocyte lacunae are oval in fibrolamellar bone, and are mostly flattened in parallel-fibered tissue. The longitudinal primary osteons occupy most of the cross section without distinct secondary reconstruction. A very thin but continuous layer of endosteal lamellae (i.e., the parallel-fibered tissue) lines the marrow cavity, suggesting a termination of dilatation of the cavity. Five LAGs are preserved in the cortex, and the widest interval is the one between the endosteal surface and the first LAG. Moving outward, the intervals between LAGs are all smaller than the first interval, but they are subequal except the outmost interval. The marrow cavity is big, and 4 LAGs are inferred to be eroded by the expansion of the medullary cavity based on the size of the marrow cavity and the interval between preserved LAGs. Noteworthy is that the parallel-fibered tissue is present only at the endosteal and periosteal surfaces of the section, unlike some small theropods in which the parallel-fibered tissues are present alternating with the fibrolamellar tissues throughout the cortical section^51–53^.

The tibial section shares similar histological features with the femur section (Fig. 3B), but has a thicker cortex (2.6 mm), smaller marrow cavity, thicker endosteal lamellae (Fig. 3B-ii), and thicker outermost avascular parallel-fibered layer (Fig. 3B-ii). Most of the lacunae are oval and flattened lacunae are rare. The tibial section also preserves 5 LAGs with 4 missing as a result of erosion of the marrow cavity.

The fibular section has a 0.8 mm thick cortex and a well-developed marrow cavity (Fig. 3C), which differs from undeveloped marrow cavities in most fibular sections of other coelurosaurians^54^. Most of the fibular cortex comprises fibrolamellar bone with well-developed longitudinal secondary osteons. Secondary remodeling occurred at least twice, as indicated by newly formed secondary osteons overlapping on the previously formed secondary osteons. The distribution of secondary osteons is uneven, laterally rare and anteriorly dense. The outermost cortex displays a fully developed EFS structure, with at least 5 tightly bunched LAGs and non-vascularized parallel-fibered tissue. The EFS in the outermost section and the endosteal lamellae located in the innermost part of the fibular section form a bistratal structure, representing convincing evidence for the termination of growth of the fibula.

## Discussion

Three Shishugou theropods, i.e., *Shishugounykus inexpectus*, *Haplocheirus sollers* and *Aorun zhaoi*, were placed at the base of the Alvarezsauria by our numerical phylogenetic analysis, with *Haplocheirus sollers* more crownward than the other two species, the phylogenetic relationships of which are unresolved relative to each other. The alvarezsaurian affinities of these three species are supported by the following synapomorphies: internal tuberosity of humerus offset from humeral head by distinct notch (char. 386.1; note that this feature is also a synapomorphy of therizinosaurians diverging after *Neimongosaurus/Erliansaurus*); lack of collateral ligament fossae on the distal condyles of metacarpal II (char. 418.1); ungual II distinctly larger than other unguals (char. 440.1); the proximal end of the lateral grooves of manual ungual II-2 partially enclosed by lateral notches (char. 446.1); the distal projection of lateral femoral distal condyle is distinctly further than medial condyle (char. 532.1); a low and rounded fibular crest (char. 543.1); and the bracing for the ascending process of the astragalus on the tibia is step-like and running proximodistally (char. 546.2).

Alvarezsaurians are among the few theropod subgroups that have only relatively small representatives (all known alvarezsaurians have a body mass less than 50 kg, smaller than most other theropod subgroups) and some late-branching alvarezsaurians are among the smallest non-avialan dinosaurs (e.g., *Xixianykus zhangi* has an adult-body-size of 0.5 kg)^10,51,55–57^. Compared to late-branching alvarezsaurians, early branching alvarezsaurians have relatively large body sizes. Based on an empirical equation for theropod body size^58^, the holotypes of the Shishugou alvarezsaurians *Shishugounykus inexpectus*, *Aorun zhaoi*, and *Haplocheirus sollers* are estimated to be about 7 kg, 4 kg, and 19 kg in body-mass, respectively; the holotypes of the Early Cretaceous alvarezsaurians *Xiyunykus pengi* and *Bannykus wulatensis* are about 15 and 24 kg, respectively; and the Late Cretaceous alvarezsaurians range in body mass from 0.25 to 50 kg, but most are lighter than 2 kg in weight. Even considering the ontogenetic variability in body-size estimation (e.g., the holotype of *Aorun zhaoi* is an early juvenile individual), the data indicates that early-branching alvarezsaurians remained at relatively large body sizes for about 50 million years in their early evolution and significant miniaturization occurred only in the Late Cretaceous. Alvarezsaurian size evolution therefore seems to be more complex than originally thought, and further studies are required, for example, determining whether the very large *Kol ghuva* indeed represents a member of the lineage. Comparing the body-size data and ontogenetic-stage data of the known early alvarezsaurian specimens, we infer that *Shishugounykus inexpectus* is probably the smallest early-branching alvarezsaurian, and is about one-third of the body-mass of the near-contemporary *Haplocheirus sollers*. This suggests that size variation in early alvarezsaurians that is greater than size variation in Late Cretaceous taxa, although sample sizes are low. Several previous studies have suggested insectivorous diets for alvarezsaurians (although *Haplocheirus* has been suggested to have been omnivorous^59^). The coexistence of two different-sized anteaters in South America, *Myrmecophaga tridactyl* and *Tamandua tetradactyla*^60^ suggests that even if the Shishugou alvarezsaurians were insectivores they could have coexisted within an ecosystem.

Body size variation among close relatives is also found in other theropods, such as tyrannosaurid theropods. The biggest tyrannosaurid *Tyrannosaurs rex* shares similar lifespan and an exponential growth period with its close but smaller relatives such as *Daspletosaurus torosus*, but the it has a much higher maximum growth rate and thus has a much larger body size^54,61^. The slow growth rate of *Shishugounykus inexpectus* holotype is supported histologically by the dominance of longitudinal canals and a relatively lower porosity. Therefore, we infer that the maximum body size of *Shishugounykus inexpectus* was constrained by its very slow growth rate. Further histological investigation is needed to test this observation, for example by comparison with histological condition of the large Shishugou alvarezsaurian *Haplocheirus sollers*.

**Figure 4:**
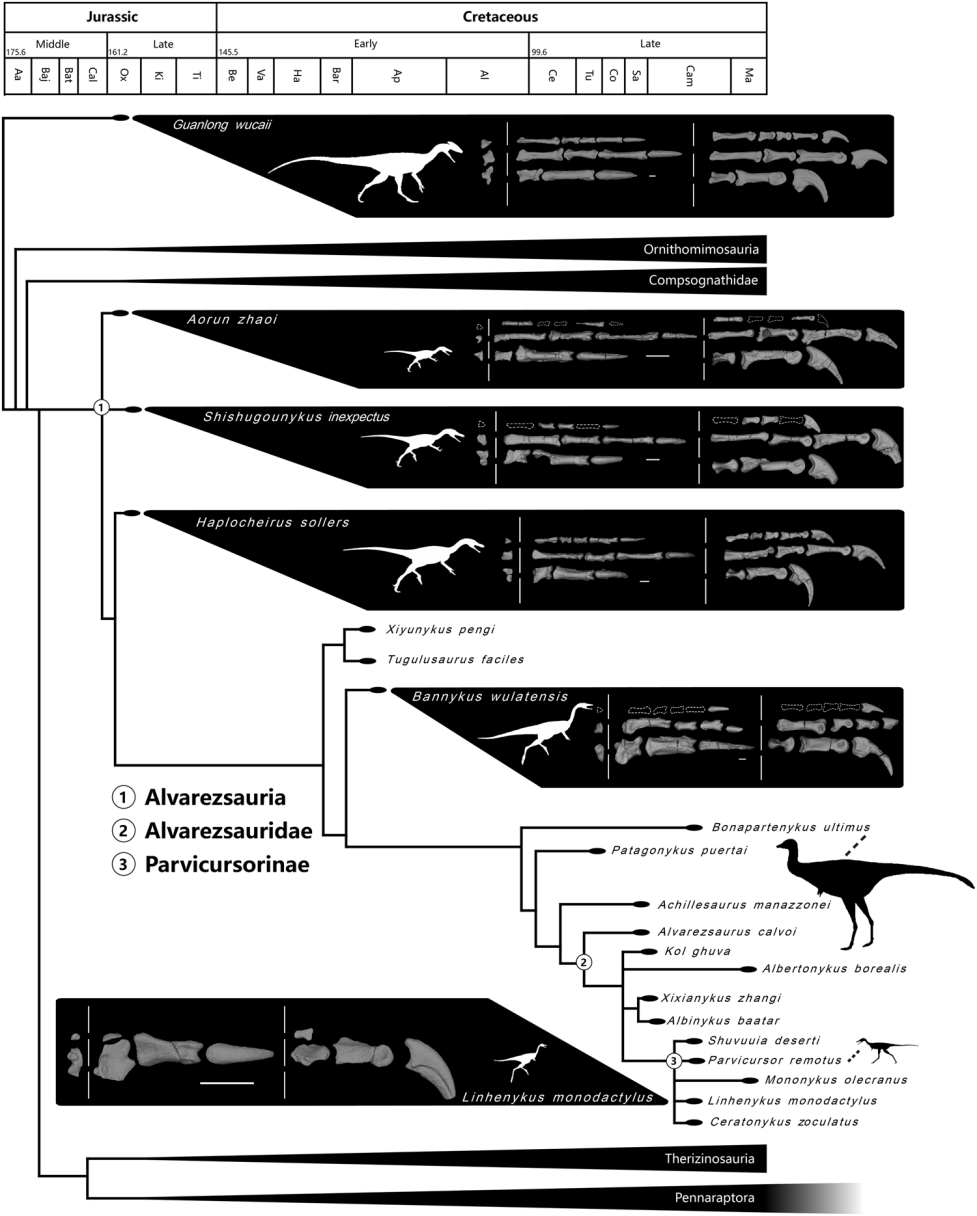
Time-calibrated alvarezsaurian phylogeny showing alvarezsaurian hand evolution (Scale bar, 10 mm); silhouettes show the size variation both in early-branching and late-branching alvarezsaurians.

Parvicursorine alvarezsaurians such as *Mononykus* and *Linhenykus* have the most specialized forelimb, and particularly the most specialized hand, among the non-avialan theropods, and these specializations have been suggested to be adaptations to fossorial activities^10,11,62^. The evolution of a typical tetanuran hand to the highly specialized hand of parvicursorines mainly involves three major modifications: broadening and flattening of the palm, enlargement and modification of the thumb, and reduction of two lateral digits. Numerous features related to these three major modifications had already evolved in some or all three Shishugou alvarezsaurians (Fig. 4): metacarpals II and III are proportionally broader transversely and thinner dorsoventrally than typical coelurosaurian metacarpals II and III, metacarpal III is laterally bowed and distally highly asymmetrical with a much larger lateral hemicondyle, metacarpal IV is proportionally shorter and thinner than typical for coelurosaurian metacarpal IV and lacks the ginglymus on its distal end, manual phalanges II-1 and -2 are enlarged and specialized in a number of features (depressing of medial and lateral surfaces and ventral axial furrowing of phalanx II-1), and manual digits III and IV displays initial shortening and narrowing.

However, these features are not equally developed in the three Shishugou alvarezsaurians (Fig. 4). *Haplocheirus sollers* has the most specialized hand, which is more similar to that of Cretaceous alvarezsaurians than that of *Shishugounykus inexpectus* and *Aorun zhaoi*. Metacarpal II is proportionally wider transversely and thinner dorsoventrally in *Haplocheirus sollers* than in *Shishugounykus inexpectus* and *Aorun zhaoi*; manual phalanx II-1 is proportionally wider transversely in *Haplocheirus sollers* than in *Shishugounykus inexpectus* and *Aorun zhaoi*, is distally curved ventrally in lateral view (straight in *Shishugounykus inexpectus* and slightly curved in *Aorun zhaoi*), and has a shaft with depressed medial and lateral surfaces (slightly convex in *Shishugounykus inexpectus* and slightly depressed in *Aorun zhaoi*) and with furrowed ventral surface (only slightly furrowed in *Shishugounykus inexpectus* and *Aorun zhaoi*); metacarpal III is laterally bowed in dorsal view (straight in *Shishugounykus inexpectus* and laterally bowed in *Aorun zhaoi*); manual phalanx III-2 is shorter than metacarpal III (subequal in *Shishugounykus inexpectus* and *Aorun zhaoi*); metacarpal IV is about 45% the length of metacarpal III (slightly shorter than metacarpal III in *Aorun zhaoi*); unguals III and IV are relatively straight in lateral view (more recurved in *Shishugounykus inexpectus* and relatively straight in *Aorun zhaoi*) and have more distally located flexor tubercles.

Interestingly, there are a few manual features in which *Shishugounykus inexpectus* and/or *Aorun zhaoi* display a condition closer to that in Cretaceous alvarezsaurians than *Haplocheirus sollers* does (Fig. 4). A pair of flexor processes are present at the proximal end of metacarpal II in *Aorun zhaoi*, but are absent in *Shishugounykus inexpectus* and *Haplocheirus sollers*; metacarpal III is dorsoventrally compressed in *Shishugounykus inexpectus*, but remains dorsoventrally deep in *Aorun zhaoi* and *Haplocheirus sollers*; metacarpal IV is much thinner than other metacarpals in *Aorun zhaoi* (it is proportionally thinner in *Haplocheirus sollers*, but not to the degree in *Aorun zhaoi*); the flexor tubercles of all unguals are strongly compressed transversely in *Aorun zhaoi* and slightly compressed in *Shishugounykus inexpectus*, but expanded in *Haplocheirus sollers*; ungual II is relatively straight in lateral view in *Aorun zhaoi*, but is strongly curved ventrally in *Shishugounykus inexpectus* and *Haplocheirus sollers*; ungual IV is about half the size of ungual III in *Shishugounykus inexpectus*, compared to two-thirds in *Aorun zhaoi* and *Haplocheirus sollers*. Finally, *Shishugounykus inexpectus* and *Aorun zhaoi* share several features related to the transverse narrowing of manual phalanges III and IV, though it is more developed in *Aorun*.

It is noteworthy that some of these features are also present in ornithomimosaurians (e.g., relatively straight unguals with distally located flexor tubercles and the presence of a pair of flexor processes at the proximal end of manual phalanx II-1), suggesting that these features are either synapomorphies of a more inclusive group comprising ornithomimosaurians, alvarezsaurians and some other theropod groups, or independently evolved in ornithomimosaurians and alvarezsaurians. Nevertheless, the variability of the anatomy in the manus of the closely related Shishugou alvarezsaurians is surprising. These features indicate that the acquisition of alvarezsaurian hand features, specifically the broadening and flattening of the palm, the enlargement and modification of the thumb, and reduction of two lateral digits, was a complex process, and there is a strong mosaic distribution of hand features in early alvarezsaurian evolution. However, the lack of multiple specimens of any of the three species means that some of these features may have been variable intraspecifically.

Our stratigraphic and radiometric data indicate that *Aorun zhaoi*, *Shishugounykus inexpectus*, and *Haplocheirus sollers* lived between 161.2 and 158.7 Ma, representing the only known Jurassic alvarezsaurians to date. The discoveries of three alvarezsaurian taxa living at roughly the same time (probably within at most 1.5 million years of each other) from the Wucaiwan Locality are unexpected, and these discoveries demonstrate an early diversification at the base of the Alvarezsauria near the Middle-Late Jurassic boundary. The earliest known alvarezsaurians not only process a taxonomical diversification and substantial body size variation (see above), but also have a large number of morphological variations.

In the phylogenetic framework set by our numerical phylogenetic analysis, 9 synapomorphies have been identified for the Alvarezsauria. Furthermore, there are at least 77 morphological differences between the three Shishugou alvarezsaurian, including 58 variations in the character-matrix and 19 additional variations (Tables S2 and S3), indicating the presence of a large number of morphological variations in the three Shishugou alvarezsaurians spanning only 1.5 million years. The new data thus suggests a large number of skeletal modifications in early alvarezsaurians, and it is particularly evident for the forelimbs, which represent the mostly specialized anatomical region of the body.

## Methods

### Descriptions and comparisons

The descriptions of the new taxon are accomplished with the observations on the holotype specimen IVPP 23567. Comparisons were based on direct observations of other specimens, published descriptions and images. It is worth mentioning that we homologize the three fingers of alvarezsaurians with digits II, III and IV of the early-branching Theropoda manus^63^.

### Phylogenetic definitions

Alvarezsauria: the most inclusive clade containing *Alvarezsaurus calvoi* but not *Passer domesticus* or *Ornithomimus edmontonicus*.

### Phylogenetic analysis

The phylogenetic analysis of IVPP V23567 was performed on a broadly sampled theropod data matrix from Xu *et al*.^43^. The dataset was assembled in Excel 2016 and analyzed on TNT v.1.5^64^. The phylogenetic analysis followed the parsimony optimality criterion performed with the New Technology Search on TNT v.1.5 with search strategies including Sectorial Searches and Tree Drift^64^. Our phylogenetic analysis provided 202 most parsimonious trees (MPTs) with a tree length of 3202 steps, a CI (consistency index) of 0.218 and a RI (retention index) of 0.602. Based on the pool of 202 MPTs, the strict consensus topologies (see Supplementary text: Fig. S1) were calculated^64^. The Absolute Bremer support test^65^ for nodes was also calculated saving suboptimal trees up to 10 steps and storing up to 10,000 trees in memory (see Supplementary text: Fig. S1).

### High-resolution CT scanning

Every element of IVPP V 23567 was scanned by a 225kv (for small skeletal elements) or a 450 kV (for large skeletal elements) micro-computerized-tomography apparatus at Key Laboratory of Vertebrate Evolution and Human Origins, CAS^66^. The initial CT scanning data was processed by a two-dimensional reconstruction software developed by the Institute of High Energy Physics, CAS^66^. And the three-dimensional segmentation and rebuilding of these data was performed on Mimics.

### Histological techniques

The ontogenetic stage of IVPP V23567 was assessed by histological analysis on ground sections from its femur, tibia and fibula. The manufacturing flow of these ground sections was performed in the histological lab at the Key Laboratory of Vertebrate Evolution and Human Origins, CAS, with standard palaeohistological techniques^67^. Specimens were embedded in resin, then cut by a circular saw (EXAKT400CP) fitted with a diamond-tipped wafering blade. By using the circular saw (EXAKT400CP) and a wheel grinder (EXAKT400CS), research materials were manufactured into thin slices with final thickness between 50 to 80 mm. Finally, each slice was capped with a glass cover slip and then labeled. After this manufacturing flow, ground sections were observed and photographed in normal and polarized light on a microscope (ZEISS Axio Imager 2) at the Key Laboratory of Vertebrate Evolution and Human Origins, CAS^68^.

### Nomenclatural acts

This published work and its nomenclatural acts have been registered in ZooBank which is a proposed online registration system for the International Code of Zoological Nomenclature (IZCN). The LSID (Life science identifiers) for this publication is urn:lsid:zoobank.org:pub:B12FD816-4CEA-4681-A4B3-AE1E6639D969. The associated information can also be viewed through any standard web browser by appending the LSID to the prefix “http://zoobank.org/”.

## Software Availability

The phylogenetic analysis was performed using TNT v.1.5 software (https://cladistics.org/tnt/); The 3D segmentation and rebuilding of the CT data was performed using Mimics (Version 10.01); The images (including the skeletal restoration and silhouettes) were produced using Adobe Illustrator CC, which is available at www.adobe.com/products/illustrator.html.

## Supplementary information

Supplementary Materials to: A new alvarezsaurian theropod from the Upper Jurassic Shishugou Formation of western China

## Data Availability

The holotype specimen IVPP V23567 is housed in the Collection House of IVPP, which is accessible to both professionals and the general public through applications. The data reported in this publication are detailed in the main text and its supplementary files.
