# Supplementary material for: A new alvarezsaurian theropod from the Upper Jurassic Shishugou Formation of western China
Source: Sci Rep. 2019 Aug 13;9:11727. doi: 10.1038/s41598-019-48148-7 (PMC6692367; doi:10.1038/s41598-019-48148-7)
Supplement: Supplementary file 1 — Supplementary Materials to: A new alvarezsaurian theropod from the Upper Jurassic Shishugou Formation of western China [file 41598_2019_48148_MOESM1_ESM.pdf]

Supplementary Materials to:

A new alvarezsaurian theropod from the Upper Jurassic Shishugou Formation of western China

Zichuan Qin[1 2 3], James Clark[4], Jonah Choiniere[5], Xing Xu[1 2*]

[1] Key Laboratory for the Evolutionary Systematics of Vertebrates, Institute of Vertebrate Paleontology and Paleoanthropology, Chinese Academy of Sciences, Beijing, 100044, China
[2] CAS Center of Excellence in Life and Paleoenvironment, Beijing, 100044, China
[3] University of Chinese Academy of Sciences, Beijing, 100044, China
[4] Department of Biological Sciences, George Washington University, Washington, DC, USA
[5] Evolutionary Studies Institute, Center of Excellence in Palaeosciences, University of the Witwatersrand, South Africa
[*]Correspondence to Xing Xu (xingxu@vip.sina.com)

Supplementary Text：
Phylogenetic analysis
Table. S1 to S3
Figure. S1
Diagnosis of *Shishugounykus inexpectus*
Figure abbreviations

Supplementary Text

Phylogenetic analysis

Character and character state descriptions. Character definitions below are cited for reference to the publication where our understandings of the characters are derived from. Where possible, we have traced the character to its original source, but in many cases this was not feasible. Citations are not intended as an exhaustive list of all uses of a given character in phylogenetic analyses.

The following characters are ordered in our analysis:
47 74 82 99 118 124 131 151 180 184 222 228 229 235 238 239 265 266 274 275 282 283 287 311 312 321 329 332 334 335 348 351 358 380 381 384 386 389 395 429 430 435 437 442 444 446 467 469 480 506 507 520 522 559 580 587

1. Contour feathers (Rauhut, 2003, #165)
0 absent
1 present

2. Vaned feathers on forelimb (Norell et al., 2001[1]; Kirkland et al., 2005 #1 [2])
0 symmetric
1 asymmetric

3. Shape of premaxillary body (portion in front of the external naris) (Rauhut, 2003, #1; in ornithomimosaurs, the premaxillary body is considerably reduced and this state scored 0)
0 wider than high, or approx. as wide as high
1 significantly higher than wide

4. Premaxillae (Benson 2009 #2; Sereno, 1998 #10; originally in Charig and Milner, 1997)
0 unfused
1 fused

5. Premaxillary-nasal suture dorsal view (Benson, 2009 #3; originally in Sereno et al 2004 #12)
0 v-shaped
1 w-shaped

6. Premaxillary-maxillary suture (Benson, 2009 #8; originally in Sereno et al., 1998 #11)
0 scarf or butt joint
1 interlocking joint

7. Premaxillary body in front of external nares (Rauhut, 2003, #2; state 2 added from Benson, 2009)
0 rostrocaudally shorter than body below nares and angle between anterior margin and alveolar margin more than 75 degrees
1 rostrocaudally longer than body below the nares and angle less than 70 degrees, naris overlaps premaxillary tooth row
2 much longer than body below naris, naris located posterior to premaxillary tooth row

8. Ventral process at the posterior end of premaxillary body (gives the posterior process a forked appearance in lateral view) (Rauhut, 2003, #4)
0 absent
1 present

9. Maxillary process of premaxilla (Norell et al., 2001; Kirkland et al., 2005 #20; also Rauhut, 2003, #6 (with character states in different order); also scored from Smith et al 2007, with states changed)
0 contacts nasal to form posterior border of nares
1 reduced so that maxilla participates broadly in external naris
2 extends posteriorly to separate maxilla from nasal posterior to nares

10. Internarial bar (Norell et al., 2001; Kirkland et al., 2005 #21)
0 dorsoventrally rounded
1 dorsoventrally flat

11. Crenulate margin on buccal edge of premaxilla (Norell et al., 2001; Kirkland et al., 2005 #22)
0 absent
1 present

12. Caudal margin of naris (Norell et al., 2001; Kirkland et al., 2005 #23)

0 farther rostral than the rostral border of the antorbital fossa
1 nearly reaching or overlapping the rostral border of the antorbital fossa

13. Premaxillary symphysis (Norell et al., 2001; Kirkland et al., 2005 #24)
0 acute, V-shaped
1 rounded, U-shaped

14. Subnarial foramen (Holtz et al., 2004 #25; Tykoski and Rowe, 2004 #11; Holtz, 1994 #25; Benson, 2009 #5)
0 absent
1 present

15. Groove on lateral surface of premaxilla, extending ventrally from the narial fossa
0 absent
1 present

16. Maxillary fenestra (Norell et al., 2001; Kirkland et al., 2005 #27, also Rauhut, 2003, #17(JNC 2007 redefined as "maxillary fenestra"; originally in Gauthier 1986)
0 absent
1 present

17. Maxillary fenestra recessed within a shallow, caudally or caudodorsally open fossa, which is itself located within the maxillary antorbital fossa (Turner et al #239, see also Witmer, 1997 p43)
0 absent
1 present

18. Longitudinal position of maxillary fenestra (Norell et al., 2001; Kirkland et al., 2005 #28 (Redefined JNC 2007)
0 situated at rostral border of antorbital fossa
1 situated posterior to rostral border of antorbital fossa

19. Latitudinal position of maxillary fenestra (Turner et al., 2007 #237)
0 situated approximately mid-height of the antorbital fossa
1 displaced dorsally in antorbital fossa

20. Foramen on caudal edge interfenestral bar between the maxillary and antorbital fenestrae
0 absent
1 present, pierces ventral portion of bar

21. Promaxillary fenestra (fenestra promaxillaris) (Norell et al., 2001; Kirkland et al., 2005 #29; also Rauhut, 2003 #16 (= 'promaxillary foramen'; JNC 2007 changed tertiary to 'promaxillary'; originally in Carpenter 1992)
0 absent
1 present

22. Palate formed by (Norell et al., 2001; Kirkland et al., 2005 #25, removed the word "secondary" to reflect that the palate is in fact the primary palate)
0 premaxilla only
1 premaxilla, maxilla and vomer

23. Palatal shelf of maxilla (Norell et al., 2001; Kirkland et al., 2005 #26)
0 flat
1 with midline ventral "tooth-like" projection

24. Ventrolateral margin of the maxilla posterior to ascending process
0 flat or rounded as it grades onto tooth row
1 developed as a sharp, ventrolaterally-projecting ridge

25. Anteroposterior length of palatal shelf of maxilla
0 short
1 long, with extensive palatal shelves

26. Orientation of the maxillae towards each other as seen in dorsal view (Rauhut, 2003, #10)
0 acutely angled
1 subparallel

27. Ascending process of the maxilla (Rauhut, 2003, #11)
0 confluent with anterior rim of maxillary body and gently sloping posterodorsally
1 offset from anterior rim of maxillary body

28. Form of anterior projection of maxilla (Reductive coding of Rauhut, 2003, #11; modified by JNC 02-19-2009)
0 offset from anterior rim of maxillary body, with anterior projection of maxillary body shorter than high
1 offset from anterior rim of maxillary body, with anterior projection of maxillary body as

long as high or longer

29. Ascending process of maxilla (Turner et al #240, modified from Gauthier, 1986, Cracraft 1986, Chiappe 1996, Clarke and Norell 2002)

0 prominent, exposed laterally and medially

1 weakly developed, lacking lateral exposure and only slight medial exposure (Most theropods, including Velociraptor mongoliensis, have a prominent ascending ramus of the maxilla. In derived avialans this lamina becomes reduced or absent.)

30. Anterior margin of maxillary antorbital fossa (Rauhut, 2003, #13; some taxa coded from Benson, 2009 #11)

0 rounded or pointed

1 square

31. Dorsal border of the internal antorbital fenestra lateral view (Turner et al 2007 #242)

0 formed by lacrimal and maxilla

1 formed by nasal and lacrimal (Turner et al 2007 #242)

32. Dorsal border of the antorbital fossa lateral view (Turner et al 2007 #243, also Rauhut, 2003, #20 (states remain the same) ; originally Sereno et al 1994)

0 formed by lacrimal and maxilla

1 formed by nasal and lacrimal

2 formed by maxilla, premaxilla and lacrimal

33. Lateral exposure of lamina of the ventral ramus of nasal process of maxilla (Turner et al 2007 #244)

0 present, large broad exposure

1 present, reduced to small triangular exposure

34. Maxillary antorbital fossa in front of the internal antorbital fenestra (Rauhut, 2003, #14; originally in Sereno et al 1996)

0 40% or less of the length of the external antorbital fenestra

1 more than 40% of the length of the external antorbital fenestra

35. Extent of antorbital fossa on jugal ramus of maxilla (Benson 2009 #12; originally in Sereno et al., 1998 #40; mentioned in Novas, 1989)

0 less than half the dorsoventral height of jugal ramus

1 more than half dorsoventral height of jugal ramus

36. Maxilla, pneumatic region on medial side of maxilla posteroventral to maxillary fenestra (Benson 2009, #16; originally mentioned in Bonaparte, 1986)

0 absent

1 present

37. Horizontal ridge on the lateral surface of maxilla at the ventral border of the antorbital fossa (Rauhut, 2003, #15; originally in Rowe and Gauthier 1986)

0 absent

1 present

38. Medial constriction between articulated premaxillae and maxillae in dorsal or ventral view (Rauhut, 2003, #8)

0 absent

1 present

39. Subnarial gap between maxilla and premaxilla at the alveolar margin (Rauhut, 2003, #9and from Benson, 2009 #10; also in Tykoski 2005)

0 absent

1 present

40. Maxillary paradental plates (Benson, 2009 #21; also Forster, 1999 #3; originally mentioned in Rauhut, 1995)

0 unfused

1 fused

41. Medial surface of maxillary paradental (interdental) plates (Benson, 2009 #23; originally in Carrano et al., 2002 #61)

0 smooth or finely pitted

1 dorsoventrally striated

42. Maxillary paradental (interdental) plates, ventral extent (Benson, 2009 #24; originally mentioned in Britt, 1991)

0 to the same ventral level as lateral maxillary wall

1 dorsal to ventral level of maxillary wall

43. Maxillary paradental plates, dorsal margin of anterior end (Benson et al., 2009 #218)

0 horizontal
1 inclined anteroventrally

44. Ventral edge of maxillary body and ventral ramus
0 ventrally flat
1 ventrally convex

45. Nasals
0 unfused
1 fused

46. Dorsal surface of the nasals (Rauhut, 2003, #18; some taxa coded from Benson, 2009 #29)
0 smooth
1 rugose

47. Nasal crest (Rauhut et al., 2009; modified according to Makovicky et al 2009, #264)
0 absent
1 present, single median crest
2 present, bilateral crests along lateral nasal margins

48. Pneumatic foramen in ventrolateral margins of the nasals (Rauhut, 2003, #19; additional taxa scored from character 38 of Smith et al 2007 supplementary info, also from Benson, 2009 #32; also Norell et al., 2001; Kirkland et al., 2005)
0 absent
1 present

49. Shape of nasals (Rauhut, 2003, #21)
0 expanding posteriorly
1 of subequal width throughout their length

50. Pronounced lateral rims of the nasals, sometimes bearing lateral cranial crests (Rauhut, 2003, #22; additional taxa coded from Benson, 2009 #28)
0 absent
1 present

51. External nares (Rauhut, 2003, #7)
0 facing laterally
1 facing anterolaterally

52. Length of nares (Rauhut et al., 2009)
0 less than 20 percent skull length
1 greater than 20 percent skull length

53. Jugal pneumatic recess in posteroventral corner of antorbital fossa (Norell et al., 2001; Kirkland et al., 2005 #34, also Rauhut, 2003, #26 (with states reversed); originally in Sereno et al 1996)
0 present
1 absent

54. Medial jugal foramen (Norell et al., 2001; Kirkland et al., 2005 #35)
0 present on medial surface ventral to postorbital bar
1 absent

55. Sublacrimal part of jugal (Rauhut, 2003, #23)
0 tapering
1 bluntly squared anteriorly
2 expanded

56. Anterior end of jugal (Rauhut, 2003, #24 reductive coding by JNC; additional taxa coded from Benson, 2009 #33; originally in Holtz, 1994 #86)
0 reaches internal antorbital fenestra
1 excluded from the internal antorbital fenestra

57. Form of anterior end of jugal (Rauhut, 2003, #24 reductive coding by JNC; additional taxa coded from Benson, 2009 #33; originally in Holtz, 1994 #86)
0 without anterior process underneath antorbital fenestra
1 expressed at the rim of the internal antorbital fenestra and with a distinct process that extends anteriorly underneath it

58. Jugal antorbital fossa (Rauhut, 2003, #25; originally in Holtz 1994with states reversed)
0 absent or developed as a slight depression
1 large, crescentic depression on the anterior end of the jugal

59. Jugal (Rauhut, 2003, #27)
0 broad, plate-like
1 very slender, rod-like

60. Jugal contribution to postorbital bar (Norell et al., 2001; Kirkland et al., 2005 #32)
0 contribute equally to postorbital bar
1 ascending process of jugal reduced
61. Anteroposterior width of postorbital bar (Zanno et al., 2009 #347)
0 subequal to preorbital bar
1 expanded, greater than twice width of preorbital bar
62. Rugosity on ventrolateral surface of jugal below orbit (Rauhut et al., 2009)
0 absent
1 present
63. Jugal and quadratojugal (Norell et al., 2001; Kirkland et al., 2005 #37)
0 separate
1 fused and not distinguishable from one another
64. Quadratojugal (Norell et al., 2001; Kirkland et al., 2005 #36; also Rauhut, 2003, #47)
[In basal theropods the quadratojugal has a small medially directed tab that wraps around the lateral surface of the quadrate and is visible in posterior view. In derived theropods, the process is anteroposteriorly long, dorsoventrally short and visible in lateral view, but not in posterior view.]
0 hook-shaped, with a dorsoventrally tall, mediolaterally short process that wraps around the lateral margin of the quadrate and is visible in posterior view
1 with a dorsoventrally short, anteroposteriorly long process only visible in lateral view
65. Quadratojugal and quadrate (JNC 3-24-2009, taken from Chiappe et al., 1998)
0 sutural connection present
1 sutural connection absent
66. Anteriormost level of jugal process of quadratojugal relative to infratemporal fenestra (Benson, 2009 #59; originally in Holtz et al., 2004 #126)
0 ventral to
1 anterior to
67. Supraorbital crests on lacrimal in adult individuals (Norell et al., 2001; Kirkland et al., 2005 #38; also Rauhut, 2003 #32; also Benson, 2009 #38)
0 absent
1 present
68. Form of supraorbital crests (Reductive coding of Norell et al., 2001; Kirkland et al., 2005 #38 by JNC 02-19-2009)
0 dorsal crest above orbit
1 lateral expansion anterior and dorsal to orbit
69. Enlarged foramen or foramina opening laterally at the angle of the lacrimal (Norell et al., 2001; Kirkland et al., 2005 #39; also Rauhut, 2003, #28; originally in Molnar et al 1990)
0 absent
1 present
70. Lacrimal foramen number
0 single
1 paired
71. Lacrimal foramina
0 exposed laterally
1 developed within a pocket formed by a lateral lacrimal sheet of bone and a rostrally open pocket in the lacrimal angle
72. Height of the lacrimal (Rauhut, 2003, #29)
0 significantly less than height of the orbit, and usually fails to reach the ventral margin of the orbit
1 as high as the orbit, and contacts jugal at the level of the ventral margin of orbit
73. Orientation of jugal ramus of lacrimal (Benson, 2009 #35; also Smith et al., 2007 #60; originally in Sereno et al., 1998 #15)
0 strongly sloping anteroventrally
1 subvertical
2 sloping posteroventrally
74. Dorsoventral thickness of maxillary ramus of lacrimal (Benson, 2009 #37; originally in Sereno et al 1994, 1996 #33)
0 very slender, much less than anteroposterior thickness of jugal ramus
1 moderate, less than or subequal to anteroposterior thickness of jugal ramus
2 greater than anteroposterior thickness of jugal ramus
75. Suborbital spur on posterior edge of ventral ramus of lacrimal (Benson, 2009 #43; also

Currie and Carpenter, 2002 #31; originally mentioned in Sampson et al., 1998)
0 absent
1 present

76. Lacrimal posterodorsal process (Norell et al., 2001; Kirkland et al., 2005 2003 #40; reductive coding by JC 2008-09-03)
0 absent
1 present

77. Length of lacrimal posterodorsal process (Norell et al., 2001; Kirkland et al., 2005 2003 #40; state added by Senter, 2007, #40)
0 subequal in length to maxillary ramus
1 much shorter than maxillary ramus

78. Direction of lacrimal posterodorsal process (Character derived from Norell et al., 2001; Kirkland et al., 2005 2003 #40; reductive coding by JC 2008-09-03; Senter, 2007, #40)
0 projects horizontally
1 projects posterodorsally or completely dorsally

79. Passage of the nasolacrimal duct (Rauhut, 2003, #30)
0 leading through the body of the ventral process of the lacrimal
1 ventral process of lacrimal not pierced, lateral side depressed below the level of the surrounding bones, and nasolacrimal duct passes lateral to the process

80. Jugal ramus of lacrimal (Smith et al 2007 #59)
0 broadly triangular, articular end nearly twice as wide anteroposteriorly as lacrimal body at lacrimal angle
1 strut-like, roughly same width anteroposteriorly throughout ventral ramus

81. Prefrontal
0 absent
1 present

82. Size of prefrontal
0 small, forms anterolateral rim of orbit with descending process proceeding along medial surface of the descending process of the lacrimal
1 small, forms small portion of skull roof and not expressed at orbital margin, no descending process
2 hypertrophied, forms portion of orbital rim and skull roof, with descending process

83. Configuration of lacrimal and frontal (Rauhut, 2003, #35)
0 lacrimal separated from frontal by prefrontal
1 lacrimal contacts frontal

84. Frontals (Norell et al., 2001; Kirkland et al., 2005 #42; also Rauhut, 2003, #36 with states reversed)
0 narrow anteriorly as a wedge between nasals
1 end abruptly anteriorly, suture with nasal transversely oriented
2 nasals extend further medially than laterally, invading anteromedial contact between frontals

85. Frontal supratemporal fossa (Turner et al 2007, #245)
0 limited extension of supratemporal fossa onto frontal
1 supratemporal fossa covers most of postorbital process of the frontal and extends anteriorly onto the dorsal surface of the frontal

86. Groove on orbital rim of frontal, possibly for reception of frontal process of postorbital (Makovicky and Turner, 2008)
0 absent
1 present

87. Anterior emargination of supratemporal fossa on frontal (Norell et al., 2001; Kirkland et al., 2005 #43)
0 straight or slightly curved
1 strongly sinusoidal and reaching onto postorbital process

88. Frontal postorbital process (dorsal view): (Norell et al., 2001; Kirkland et al., 2005 #44)
0 smooth transition from orbital margin
1 sharply demarcated from orbital margin

89. Frontal edge (Norell et al., 2001; Kirkland et al., 2005 #45)
0 smooth in region of lacrimal suture
1 edge notched

90. Postorbital in lateral view (Norell et al., 2001; Kirkland et al., 2005 #4)

0 with straight anterior (frontal) process
1 frontal process curves anterodorsally and dorsal border of temporal bar is dorsally concave

91. Lateral surface of anterior process of postorbital (Benson, 2009 #45; originally in Carrano et al., 2002 #24)
0 thin and unornamented
1 dorsoventrally thickened into a laterally projecting and rugose platform

92. Contact between lacrimal and postorbital (Rauhut, 2003, #39; originally in Sampson et al 1998, also Benson, 2009 #44)
0 absent
1 present

93. Cross-section of the ventral process of the postorbital (Rauhut, 2003, #41; originally in Sereno et 1994, 1996, also Benson, 2009 #49 states 1 and 2)
0 triangular
1 U-shaped

94. Jugal process of the postorbital (Rauhut, 2003 #40; also Norell et al., 2001; Kirkland et al., 2005 #3 (with states reversed), also Benson, 2009 #50 with reductive coding; originally in Harris, 1998 #9)
0 ventrally directed and tapering
1 with suborbital anterior spur

95. Postorbital jugal process form of anterior suborbital spur (Benson, 2009 #50 with reductive coding; originally in Harris, 1998 #9)
0 small
1 large curving flange

96. Supraorbital shelf formed mostly by an additional ossification (palpebral) (Benson, 2009 #46; originally in Coria and Currie, 2006 #11)
0 absent
1 present

97. Orbit (Norell et al., 2001; Kirkland et al., 2005 #2)
0 circular in lateral or dorsolateral view
1 dorsoventrally taller than anteroposteriorly wide, often keyhole shaped

98. Parietals (Norell et al., 2001; Kirkland et al., 2005 #47)
0 separate
1 fused

99. Parietal supratemporal fenestra (Rauhut, 2003, #43and added state 2; originally in Molnar et al 1990; State 0 of this character the same as state 0 of character 45 in Turner et al 2007 supplementary information, but states 1 and 2 of that character are not comparable)
0 separated by a horizontal plate formed by the parietals
1 contact each other posteriorly, but separated anteriorly by an anteriorly widening triangular plate formed by the parietals
2 nearly confluent over parietals and only separated by a thin line of bone along the sagittal suture

100. Anteromedial corner of supratemporal fossa (Benson, 2009 #61; also Forster, 1999 #33; originally mentioned in Coria and Currie, 2002)
0 open dorsally
1 roofed by shelf of frontoparietal

101. Sagittal crest (Norell et al., 2001; Kirkland et al., 2005 #46; state 0 removed and coded as state 0 for Rauhut #43, states 1 and 2 converted to 0 and 1, respectively)
0 dorsal surface of parietals smooth with no sagittal crest
1 sagittal crest present

102. Form of sagittal crest (Reductive coding of Norell et al., 2001; Kirkland et al., 2005 #46 by JNC 02-19-2009)
0 parietals dorsally convex with very low sagittal crest along midline
1 dorsally convex with well-developed sagittal crest

103. Posteriorly placed, knob-like dorsal projection of the parietals (Rauhut, 2003, #42; originally in Coria and Currie 2002)
0 absent
1 present

104. Connections of quadratojugal process of squamosal (Norell et al., 2001; Kirkland et al., 2005 #49; also Rauhut, 2003, #46, with state 0 and 2 coded as 1 and state 1 coded as 0)

0 contacts quadratojugal
1 does not contact quadratojugal

105. Infratemporal fenestra shape (Norell et al., 2001; Kirkland et al., 2005 #5 wording modified; also Holtz et al., 2004 #112; originally in Allain, 2002 #31; additional taxa scored from Benson, 2009 #51)
0 rectangular, postorbital bar parallels quadratojugal and squamosal articular area
1 lower temporal fenestra constricted mesially by squamosal and quadratojugal approaching postorbital bar

106. Shape of quadratojugal process of the squamosal (Rauhut, 2003, #45)
0 tapering
1 broad, and usually somewhat expanded

107. Posterolateral shelf on squamosal overhanging quadrate head (Norell et al., 2001; Kirkland et al., 2005 #50)
0 absent
1 present

108. Quadrate head (Norell et al., 2001; Kirkland et al., 2005 #213)
0 covered by squamosal in lateral view
1 quadrate cotyle of squamosal open laterally exposing quadrate head

109. Descending process of squamosal (Norell et al., 2001; Kirkland et al., 2005 #48)
0 parallels quadrate shaft
1 nearly perpendicular to quadrate shaft

110. Supratemporal fenestra (Norell et al., 2001; Kirkland et al., 2005 #216)
0 bounded laterally and posteriorly by the squamosal
1 supratemporal fenestra extended as a fossa on to the dorsal surface of the squamosal

111. Quadrate (Norell et al., 2001; Kirkland et al., 2005 #53; also Rauhut, 2003 #48, also Benson, 2009 #54)
0 solid
1 hollow

112. Mandibular joint (Rauhut, 2003 #51; also Norell et al., 2001; Kirkland et al., 2005 #52 (with state 1 recoded as state 2); additional taxa scored from Benson, 2009 #56)
0 approximately straight below quadrate head
1 significantly posterior to quadrate head
2 significantly anterior to quadrate head

113. Quadrate medial pneumatic recess (depression and foramen in the area of the mandibular condyle on medial surface) (Benson, 2009 #57, also Eddy, 2008; originally mentioned in Britt, 1991)
0 absent
1 fossa adjacent to mandibular condyle, foramen at base of pterygoid ramus

114. Quadrate posterior pneumatic recess (This character described in Eddy, 2008 #58)
0 absent
1 present as a lens-shaped fossa extending dorsally or dorsomedially from the quadrate foramen

115. Dorsal end of the quadrate (Rauhut, 2003, #50; also Norell et al., 2001; Kirkland et al., 2005 #51)
0 with a single head that fits into a slot on the ventral side of the squamosal
1 double-headed, medial head contacts the braincase

116. Quadrate foramen (Reductive coding of Rauhut 2003 #49)
0 absent
1 present

117. Quadrate foramen (Rauhut, 2003, #49; also scored with reference to Kirkland et al #55 with states reversed)
0 developed as a distinct opening between the quadrate and quadratojugal
1 almost entirely closed in the quadrate

118. Ectopterygoid (Rauhut, 2003 #67; character states slightly changed by JC 2008-07-09)
0 slender, without ventral fossa
1 expanded, with a ventral depression medially
2 expanded, with a deep groove leading into the ectopterygoid body medially
3 deeply excavated and medial opening constricted into a foramen

119. Dorsal recess on ectopterygoid (Norell et al., 2001; Kirkland et al., 2005 #61)
0 absent

1 present

120. Ectopterygoid (Rauhut, 2003, #66)
0 posterior to palatine
1 lateral to palatine

121. Palatine and ectopterygoid (Norell et al., 2001; Kirkland et al., 2005 #63 (after Currie 1995))
0 separated by pterygoid
1 contact

122. Contact between pterygoid and palatine (Rauhut 2003, #68; originally in Ostrom, 1969)
0 continuous
1 discontinuous in the mid-region, resulting in a subsidiary palatal fenestra

123. Flange of pterygoid (Norell et al., 2001; Kirkland et al., 2005 #62)
0 well developed
1 reduced in size or absent

124. Shape of palatine in ventral view (Rauhut, 2003, #65; also Norell et al., 2001; Kirkland et al., 2005 #64 (with 0 recoded as state 1 and state 1 recoded as state 2); originally in Harris, 1998)
0 plate-like trapezoidal or subrectangular
1 tetraradiate
2 jugal process strongly reduced or absent

125. Suborbital fenestra (Norell et al., 2001; Kirkland et al., 2005 #65)
0 similar in length to orbit
1 reduced in size or absent

126. Infratemporal fenestra (Rauhut, 2003, #38)
0 smaller than or subequal in size to orbit
1 strongly enlarged, more than 1.5 times the size of the orbit

127. Postorbital part of the skull roof (Rauhut, 2003, #44)
0 as high as orbital region
1 deflected ventrally in adult individuals

128. Preorbital region of the skull in post-hatchling individuals (Rauhut, 2003, #71)
0 elongate, nasals considerably longer than frontals, maxilla at least twice the length of the premaxilla
1 shortened, nasals subequal in length to frontals or shorter, maxillary length less than twice the length of the premaxillary body

129. Occipital region of the skull faces (Benson, 2009 #65; also Currie et al., 2003 #63, Forster, 1999 #43, Coria and Currie, 2002; originally mentioned in Currie and Zhao, 1994)
0 posteriorly
1 posteroventrally

130. Basipterygoid processes (Norell et al., 2001; Kirkland et al., 2005 #13)
0 well-developed, extending as a distinct process from the base of the basisphenoid
1 abbreviated or absent

131. Basipterygoid processes well developed and (Rauhut 2003 #58. State 2 removed and recoded in #62 as state 1 (basipterygoid processes abbreviated or absent). State 2 added by JC 2008-08-09)
0 anteroposteriorly short and finger-like (approximately as long as wide)
1 longer than wide
2 significantly elongated and tapering

132. Basipterygoid processes (Norell et al., 2001; Kirkland et al., 2005 #12)
0 ventral or anteroventrally projecting
1 lateroventrally projecting
2 caudally projecting

133. Basipterygoid processes (Norell et al., 2001; Kirkland et al., 2005 #14)
0 solid
1 hollow

134. Basipterygoid recesses on dorsolateral surfaces of basipterygoid processes (Norell et al., 2001; Kirkland et al., 2005 #15)
0 absent
1 present

135. Basisphenoid bulla (Zanno et al., 2009)
0 absent
1 present

136. Paired foramina at anterior end of basisphenoid recess, separated by a thin bar of bone [New character. Added to homologize condition in Shuvuuia and *Xiyunykus* . In Shuvuuia, these

foramina are hypertrophied and developed outside of the basisphenoid recess (which has been lost), but they agree closely in topological position with the foramina from the Tugulu specimen. Many theropods have paired foramina at the posterior end of the basisphenoid recess (e.g., Dromaeosaurids), but having paired foramina at the anterior end is to our knowledge only known in alvarezsaurids.]

0 absent, single or no opening may be present
1 present

137. Basisphenoid recess (Rauhut, 2003, #57, states 1 and 2 recoded as 1 and coded for character 104 as 0 and 1 respectively)
0 absent or poorly developed
1 deep and well-developed

138. Passage of internal carotids between posterior end of skull and pituitary fossa
[New character. In derived alvarazsaurids, e.g., *Shuvuuia* and in some small-bodied troodontids, e.g., *Byronosaurus*, the internal carotids are clearly demarcated by hemicylindrical bony tubes projecting ventrally from the ventral surface of the basisphenoid]
0 no bony tubes present
1 enclosed by bony tubes extending along ventral surface of basisphenoid

139. Basisphenoid recess position (Norell et al., 2001; Kirkland et al., 2005 #9 (with state 2 removed and coded as 0 for #57 of Rauhut, 2003))
0 between basisphenoid and basioccipital
1 entirely within basisphenoid

140. Posterior opening of basisphenoid recess (Norell et al., 2001; Kirkland et al., 2005 #10)
0 single
1 divided into two small, circular foramina by a thin bar of bone

141. Basisphenoid between basal tubera and basipterygoid processes (Rauhut, 2003, #56)
0 approximately as wide as long, or wider
1 significantly elongated, at least 1.5 times longer than wide

142. Basisphenoid in lateral view
0 oriented subhorizontally
1 anterior portion located much more ventrally than posterior portion, recess visible in posterior view

143. Base of cultriform process (Norell et al., 2001; Kirkland et al., 2005 #11; also Rauhut 2003 #62. Character codings the same and characters combined.)
0 not highly pneumatised
1 expanded and pneumatic (parasphenoid bulla)

144. Cultriform process, dorsal surface
[New character. In *Xiyunykus* and in some dromaeosaurs, e.g., *Tsaagan*, the dorsal surface of the cultriform process bears a deep groove along its length. We add this character in the hopes it will be informative when braincase features become more broadly known in transitional alvarezsauroids and other maniraptoran taxa.]
0 without deep longitudinal groove
1 with deep axial groove

145. Cultriform process, ventral surface
[New character. In *Shuvuuia* and in *Xiyunykus*, the ventral surface of the cultriform process bears a keel-like ridge along much of its length. This feature is certainly absent in *Haplocheirus*, but its distribution within the rest of Alvarezsauroidea is poorly known. At least one other maniraptoran, *Citipati*, possesses this feature homoplastically, but its distribution needs more research to determine the .]
0 without ventrally projecting, mediolaterally narrow ridge
1 with ridge

146. Exits of CN X-XII (Norell et al., 2001; Kirkland et al., 2005 #19)
0 flush with surface of exoccipital
1 located together in a bowl-like basisphenoid depression

147. Exits of CN X and XI (Rauhut 2003, #60; originally mentioned in Chatterjee, 1993; additional taxa scored from Benson, 2009 #69)
0 laterally through the jugular foramen
1 posteriorly through a foramen (metotic foramen) lateral to the exit of cranial nerve XII and the occipital condyle

148. Exoccipital lateral to occipital condyle (Added by JNC Oct 2011 to assess homology between

this region in troodontids and parvicursorines)
0 forms roof over exits for CN X and XII
1 unexpanded and does not form roof

149. Supraoccipital sagittal crest (Zanno et al., 2009, #256)
0 with pronounced sagittal crest
1 sagittal crest reduced or absent

150. Paroccipital process shape (Norell et al., 2001; Kirkland et al., 2005 #57)
0 elongate and slender
1 short, deep

151. Paroccipital process direction (Norell et al., 2001; Kirkland et al., 2005 #58; also Rauhut, 2003, #52; also Harris, 1998 #24; originally mentioned in Paul, 1988; state 2 of Rauhut recoded as state 1 of Kirkland, also Benson, 2009 #71; state 2 added by JNC to differentiate condition in Oviraptorosaurs)
0 straight, projects laterally or posterolaterally
1 project ventrolaterally
2 pendant

152. Paroccipital process dorsal edge (Norell et al., 2001; Kirkland et al., 2005 #59; originally in Currie 1995)
0 with straight dorsal edge
1 distal end twists rostrally, distal ends of the processes oriented transversely rather than vertically

153. Ventral rim of the basis of the paroccipital processes (Rauhut, 2003, #54, also Benson, 2009 #72)
0 above or level with the dorsal border of the occipital condyle
1 situated at mid-height of occipital condyle or lower

154. Foramen magnum (Norell et al., 2001; Kirkland et al., 2005 #55)
0 subcircular, slightly wider than tall
1 oval, taller than wide

155. Foramen magnum size (Maryanska et al., 2002, scorings from Zanno et al., 2009 #257)
0 smaller than or subequal in width to occipital condyle
1 larger in width than occipital condyle

156. Occipital condyle (Norell et al., 2001; Kirkland et al., 2005 #56)
0 without constricted neck
1 subspherical with constricted neck

157. Infracondylar fossa of occipital condyle
0 absent
1 present

158. Form of infracondylar fossa of occipital condyle (Benson, 2009 #67; originally mentioned in Allain, 2002)
0 narrow and groove-like
1 broad depression approximately two-thirds the width of the occipital condyle

159. Basal tubera (Zanno et al., 2009, #254)
0 present
1 absent

160. Basal tubera composition (Rauhut, 2003, #55)
0 equally formed by basioccipital and basisphenoid and not subdivided
1 subdivided by a lateral longitudinal groove into a medial part entriely formed by the basioccipital, and a lateral part, entirely formed by the basisphenoid

161. Basal tubera spacing (Norell et al., 2001; Kirkland et al., 2005 #222; originally in Holtz, 2000 #97, also Benson, 2009 #66)
0 set far apart, level with or beyond lateral edge of occipital condyle and/or foramen magnum (may be connected by a web of bone or separated by a large notch)
1 tubera small, directly below condyle and foramen magnum, and separated by a narrow notch

162. Subcondylar recess (Norell et al., 2001; Kirkland et al., 2005 #223; wording changed by JC 2008-07-07)
0 absent
1 present in basioccipital/exoccipital lateral and ventral to occipital condyle

163. Subcondylar recess form (Zanno et al., 2009, #252 with reductive coding by JNC)
0 isolated from nervous foramina CNX-CNXII

1 subcondylar recess and cranial nerves exit together in a deep depression encompassing multiple pneumatic fossae and enclosed by a well-developed rim

164. Exit of mid-cerebral vein (Rauhut, 2003, #61)
0 included in trigeminal foramen
1 vein exits braincase through a separate foramen anterodorsal to the trigeminal foramen

165. Brain proportions (Rauhut, 2003, #64)
0 forebrain small and narrow
1 forebrain significantly enlarged and triangular

166. Anterior tympanic recess in the braincase (Rauhut, 2003, #59; Originally in Makovicky and Sues 1998)
0 absent
1 present

167. Prootic pneumatic recess (Norell et al., 2001; Kirkland et al., 2005 #16; this feature is located on the lateral surface at the junction between prootic, basispenoid and basioccipital, on the dorsolateral margin of the basal tuber.)
0 absent
1 present

168. Form of pneumatic prootic recess (Reductive coding by JNC of Norell et al., 2001; Kirkland et al., 2005)
0 dorsally open fossa on prootic/opisthotic
1 deep, posterolaterally directed concavity

169. Crista interfenestralis (Norell et al., 2001; Kirkland et al., 2005 #7)
0 confluent with lateral surface of prootic and opisthotic
1 distinctly depressed within middle ear opening

170. Accessory dorsal tympanic recess (dorsal to crista interfenestralis) (Norell et al., 2001; Kirkland et al., 2005 #17; edited by JC 2008-07-07)
0 absent
1 present

171. Form of dorsal tympanic recess (This character also scored from state 1 of Rauhut, 2003 #53)
0 small pocket present
1 extensive with indirect pneumatisation

172. Caudal (posterior) tympanic recess (Norell et al., 2001; Kirkland et al., 2005 #18)
0 absent
1 present

173. Form of caudal tympanic recess (This character also scored from state 1 of Rauhut, 2003 #53)
0 present as opening on anterior surface of paroccipital process
1 extends into opisthotic posterodorsal to fenestra ovalis, confluent with this fenestra

174. Exoccipitals ventral to posterior pneumatic recess
0 no lip
1 form anteriorly projecting, posterodorsally curling, dorsally concave, tablike process

175. Otosphenoidal crest (Norell et al., 2001; Kirkland et al., 2005 #6)
0 vertical on basisphenoid and prootic, and does not border an enlarged pneumatic recess
1 well-developed, crescent-shaped, thin crest forms anterior edge of enlarged pneumatic recess

176. Subotic recess (pneumatic fossa ventral to fenestra ovalis) (Norell et al., 2001; Kirkland et al., 2005 #8)
0 absent
1 present

177. Depression (possibly pneumatic) on ventral surface of postorbital process of laterosphenoid (Norell et al., 2001; Kirkland et al., 2005 #221)
0 absent
1 present

178. Interorbital region in adults (Benson, 2009 #74; originally in Forster, 1999 #42)
0 unossified
1 ossified

179. Prominent endocranial expansion of vertical semicircular canal (Often forms a "vestibular pyramid", a mound-like structure on the posteroventral edge of the semicircular canal)
0 absent
1 present

180. Mandibular foramen (Gohlich and Chiappe 2006 #71, modified by JNC: added state 2))

0 absent or reduced
1 large
2 hypertrophied, greater than 50% dentary length

181. Shape of mandibular foramen (Norell et al., 2001; Kirkland et al., 2005 #73)
0 oval
1 subdivided by a spinous rostral process of the surangular

182. Paradental plates of dentary (Norell et al., 2001; Kirkland et al., 2005 #90)
0 lack paradental plates
1 with paradental plates on the medial surface of the tooth row

183. Internal mandibular fenestra (Norell et al., 2001; Kirkland et al., 2005 #74)
0 small and slit-like
1 large and rounded

184. Shape of anterior end of dentary (Benson, 2009 #77 modified by JNC; also Smith et al., 2007 #125; originally in Sereno et al., 2996 #50 and Sereno et al., 1998 #6)
0 blunt and unexpanded
1 dorsoventrally expanded, rounded and slightly upturned
2 with anteroventral process giving a "squared off" appearance in lateral view

185. Dorsal edge of anterior end of dentary in lateral view
[In spinosaurids, *Masiakasaurus*, and in *Haplocheirus*, there is a dorsally arcing eminence on the anterior tip of the dentary along the dorsal margin, but given the phylogenetic distance between these taxa this feature is likely a homoplasy. The distribution of this character within transitional alvarezsauroids, however, is virtually unknown because there is so little cranial material]
0 dorsally flat
1 with dorsally expanded, arcuate eminence

186. Symphyseal region of dentary (Norell et al., 2001; Kirkland et al., 2005 #66 reductive coding by JNC; see also Rauhut, 2003 #76)
0 Broad and straight, paralleling lateral margin
1 medially recurved

187. Degree of medial recurvature of dentary symphysis
0 medially recurved slightly
1 strongly recurved medially

188. Dentary symphyseal fusion
0 absent
1 present

189. Dentary anterior end in lateral view (Norell et al., 2001; Kirkland et al., 2005 #67, also Norell et al., 2001; Kirkland et al., 2005 #212, combined by JNC due to non-independence and modified for clarity)
0 in line with main part of buccal edge
1 anterior end deflected ventrally

190. Width of dentary symphyseal region (Clark et al., 2004, scorings from Zanno et al., 2009 #260)
0 no broader than transverse width of post-symphyseal region
1 broader than post-symphyseal region

191. Orientation of dentary symphysis in lateral view (Zanno et al., 2009, #261)
0 vertical to subvertical
1 projects strongly cranially, oblique with respect to dentary ventral margin

192. Posterior end of dentary (Norell et al., 2001; Kirkland et al., 2005 #69; also Rauhut, 2003 #77 reductive coding by JNC 2009-05-13)
0 without posterodorsal process dorsal to mandibular fenestra
1 with dorsal process

193. Form of dentary posterodorsal process (Reductive coding of Norell et al., 2001; Kirkland et al., 2005 #69; also Rauhut, 2003 #77 by JNC 2009-05-13)
0 developed only above anterior end of mandibular fenestra
1 with elongate dorsal process extending over most of fenestra

194. Labial face of dentary (Norell et al., 2001; Kirkland et al., 2005 #70)
0 flat
1 with lateral ridge and inset tooth row

195. Nutrient foramina on external surface of dentary (Norell et al., 2001; Kirkland et al., 2005 #72 reductive coding by JNC; state 1 added by JC 2008-07-29 to homologize condition in troodontids; additional taxa coded from Benson, 2009 #80)

0 superficial
1 descend strongly posteriorly within a deep groove

196. Form of nutrient foraminal groove (Reductive coding of Norell et al., 2001; Kirkland et al., 2005 #72; state 1 added by JC 2008-07-29 to homologize condition in troodontids;)
0 thin groove of constant height as it extends posteriorly
1 posterior end of groove is dorsoventrally expanded

197. Dentary shape in lateral view (Norell et al., 2001; Kirkland et al., 2005 #71)
0 with subparallel dorsal and ventral edges
1 subtriangular in lateral view

198. Form of triangular dentary (character added by JNC to homologize condition in some oviraptorosaurs)
0 low triangular
1 high triangular

199. Ventral edge of dentary in lateral view (Norell et al., 2001; Kirkland et al., 2005 #224)
0 straight or nearly straight
1 descends strongly posteriorly

200. Dentary paradental groove separating interdental plates from medial wall of dentary (This character added after personal discussion with Roger Benson, described in Benson 2008; additional taxa scored from Benson, 2009 #81)
0 absent
1 present

201. Pronounced coronoid eminence on the surangular (Rauhut, 2003 #72; also Norell et al., 2001; Kirkland et al., 2005 #68)
0 absent
1 present (Rauhut, 2003 #72; also Norell et al., 2001; Kirkland et al., 2005 #68)

202. Foramen in lateral surface of surangular rostral to mandibular articulation (Norell et al., 2001; Kirkland et al., 2005 #75)
0 absent
1 present

203. Number of surangular foramina (Benson, 2009 #100; originally mentioned in Currie and Zhao, 1994)
0 one
1 two

204. Laterally inclined flange along lateral surface of surangular (Norell et al., 2001; Kirkland et al., 2005 #209; also Holtz, 2000 #116, Tykoski and Rowe, 2004 #60; originally in Holtz 1998, also Benson, 2009 #98)
0 absent
1 present

205. Position of lateral flange along dorsal side of lateral surface of surangular
[New character. Basal alvarezsauroids and many theropods, e.g., tyrannosauroids, ornithomimosaurs, bear a laterally projecting flange along the lateral surface of the surangular. In most theropod taxa, the flange is set below the level of the dorsal margin of the surangular. However, in the basal alvarezsauroids *Haplocheirus*, *Xiyunykus*, and *Bannykus*, and in the spinosaurid *Baryonyx*, the flange is developed along the dorsal margin of the bone, often resulting in a flattened, platform-like surface on the dorsal margin of the surangular.]
0 ventral to dorsal margin
1 along lateral side of dorsal margin

206. Anterior portion of the surangular (Rauhut, 2003, #75; originally in Gauthier 1986, also Benson, 2009 #99)
0 less than half the height of the mandible above the mandibular fenestra
1 more than half the height of the mandible at the level of the mandibular fenestra

207. Retroarticular process of the mandible (Rauhut, 2003, #73; also Norell et al., 2001; Kirkland et al., 2005 #79 (with states reversed); originally in Sereno et al 1996, also Harris 1998)
0 narrow, rod-like
1 broadened, with groove posteriorly for the attachment of the m. depressor mandibulae

208. Attachment of the m. depressor mandibulae on retroarcticular process of mandible (Rauhut, 2003, #74; originally in Sereno et al 1996)
0 facing dorsally
1 facing posterodorsally

209. Retroarticular process (Norell et al., 2001; Kirkland et al., 2005 #219)

0 points posteriorly
1 curves gently posterodorsally

210. Articular (Norell et al., 2001; Kirkland et al., 2005 #78)
0 without elongate, slender medial, posteromedial, or mediodorsal process from retroarticular process
1 with process

211. Angular (Norell et al., 2001; Kirkland et al., 2005 #208)
0 exposed almost to end of mandible in lateral view, reaches or almost reaches articular
1 excluded from posterior end angular suture turns ventrally and meets ventral border of mandible rostral to glenoid

212. Coronoid ossification (Norell et al., 2001; Kirkland et al., 2005 #77; also Rauhut, 2003, #80)
0 absent
1 present

213. Form of coronoid ossification (Reductive coding of Norell et al., 2001; Kirkland et al., 2005 #77 by JNC 02-19-2009)
0 large
1 thin splint

214. Splenial (Norell et al., 2001; Kirkland et al., 2005 #76 with states reversed)
0 not widely exposed on lateral surface of mandible
1 exposed as a broad triangle between dentary and angular on lateral surface of mandible

215. Foramen in the ventral part of the splenial (mylohyal foramen) (Rauhut, 2003, #78; reductive coding by JC 2008-09-11)
0 absent
1 present

216. Form of mylohyal foramen (Reductive coding of Rauhut, 2003 #78; also Holtz et al., 2004 #239; additional taxa coded from Benson, 2009 #97)
0 completely enclosed in the splenial
1 opened anteroventrally

217. Posterior end of splenial (Rauhut, 2003, #79; originally in Sereno et al 1996)
0 straight
1 forked

218. Articular glenoid fossa (Norell et al., 2001; Kirkland et al., 2005 #80)
0 as long as distal end of quadrate
1 twice or more as long as quadrate surface, allowing anteroposterior movement of mandible

219. Palatal teeth (Rauhut, 2003, #69)
0 present
1 absent

220. Premaxillary teeth (Rauhut, 2003, #81; also Norell et al., 2001; Kirkland et al., 2005 #81)
0 present
1 absent

221. Number of premaxillary teeth (Rauhut, 2003, #5)
0 three
1 four
2 five
3 more than five

222. First premaxillary tooth size (Turner et al 2007, #251; also in Cuurie and Varrichio, 2004 #42; and in Currie, 1995)
0 slightly smaller or the same size as 2 and 3
1 much smaller than 2 and 3
2 much larger than 2 and 3

223. Second premaxillary tooth (Norell et al., 2001; Kirkland et al., 2005 #82)
0 approximately equivalent in size to other premaxillary teeth
1 markedly larger than third and fourth premaxillary teeth

224. Premaxillary tooth direction
0 decumbent or ventrally projecting
1 procumbent

225. Serrations on premaxillary teeth (Rauhut, 2003, #84)
0 present
1 absent

226. In cross section, premaxillary tooth crowns (Norell et al., 2001; Kirkland et al., 2005 #91; originally in Bakker, 1988)
0 sub-oval to sub-circular
1 D-shaped with flat lingual surface
227. Maxillary teeth (Norell et al., 2001; Kirkland et al., 2005 #83)
0 present
1 absent (Norell et al., 2001; Kirkland et al., 2005 #83)
228. Length of maxillary tooth row (Rauhut, 2003, #70, also Benson, 2009 #92)
0 extends posteriorly to approximately half the length of the orbit
1 ends at the anterior rim of the orbit
2 completely antorbital, tooth row ends anterior to the vertical strut of the lacrimal
3 ends below the junction between the maxillary body and the ascending process
229. Number of maxillary teeth (Benson, 2009 #86; originally in Sereno et al., 1998 #26)
0 10-14
1 15-19
2 20 or more
230. Maxillary tooth direction
0 ventrally or posteriorly inclined
1 procumbent
231. Maxillary and dentary teeth, mesial (anterior) carina (Benson et al., 2009 #220)
0 present
1 absent
232. Mesial (anterior) carina of maxillary and dentary teeth present and (Benson, 2009 #89)
0 extends to base of crown
1 terminates ventrally at approximately mid-crown level or more apically
233. Shape of maxillary teeth (Modified from Clark et al., 1994, state (2) added from Senter, 2007, also Zanno et al., 2009, #267)
0 labiolingually flattened, apicobasally taller than mesiodistally wide
1 lanceolate and subsymmetrical (as in therizinosaurs)
2 simple, conical, incisive crowns (as in Alvarezsaurs)
234. Degree of curvature of maxillary tooth crowns (Benson, 2009 #96; also Sereno et al., 1998, Deperet and Savornin, 1928; originally mentioned in Stromer, 1915)
0 crowns curve posteriorly as they extend distally
1 very little curvature or crowns straight
235. Serrations on maxillary and dentary teeth (Norell et al., 2001; Kirkland et al., 2005 #84; also Rauhut, 2003, #82, also Benson, 2009 #88; originally in Chiappe 1996)
0 present
1 some without serrations anteriorly (except at base in *Saurornithoides mongoliensis*)
2 absent
236. Maxillary tooth implantation
0 separate alveoli
1 set in an open groove
237. Roots of maxillary and dentary teeth (Norell et al., 2001; Kirkland et al., 2005 #228; additional taxa scored from Benson, 2009 #91)
0 mediolaterally compressed
1 circular in cross-section
238. Dentary tooth row (Norell et al., 2001; Kirkland et al., 2005 #217, modified by JNC)
0 fully toothed
1 only teeth rostrally
2 edentulous
3 fully toothed with short edentulous anterior portion
239. Number of dentary teeth (Norell et al., 2001; Kirkland et al., 2005 #85)
0 large, fewer than 25 in dentary
1 moderate number of small teeth (25-30 in dentary)
2 relatively small and numerous (more than 30 in dentary)
240. Dentary teeth distribution (Norell et al., 2001; Kirkland et al., 2005 #231; also Rauhut, 2003 #83; state 2 added by JNC as a potential Troodontid synapomorphy)
0 homodont
1 increasing in size anteriorly, becoming more conical in shape
2 Decreasing in size anteriorly, becoming more densely packed

241. Shape of dentary teeth (Norell et al., 2001; Kirkland et al., 2005 #230 modified by JNC)
0 labiolingually flattened, apicobasally taller than mesiodistally wide
1 lanceolate and subsymmetrical (as in therizinosauroids)
2 simple, conical, incisive crowns (as in Alvarezsaurs)
242. Third dentary alveolus (Benson, 2009 #79; also Harris, 1998 #48, Holtz et al., 2004 #213, Sereno et al., 2004 #71; originally in Gauthier, 1986 #36)
0 subequal in size to other alveoli
1 circular and enlarged relative to other alveoli
243. Dentary tooth implantation (Turner et al 2007 #85; originally in Currie 1987)
0 separate alveoli
1 set in an open groove
244. Dentary tooth direction
0 dorsally or posteriorly inclined
1 procumbent (anteriorly inclined)
245. Serrations on maxillary and dentary teeth (Norell et al., 2001; Kirkland et al., 2005 #87)
0 simple, denticles convex
1 distal and often mesial edges of teeth with large, hooked denticles that point toward the tip of the crown
246. Serration size (Norell et al., 2001; Kirkland et al., 2005 #86)
0 large
1 small
247. Constriction between tooth crown and root (Rauhut, 2003, #87; also Norell et al., 2001; Kirkland et al., 2005 #88 (with states reversed))
0 absent
1 present
248. Enamel of tooth crowns (Reductive coding of Benson, 2009 #95; see Brusatte et al 2007 for a discussion of the distribution of this character)
0 smooth
1 horizontally wrinkled, especially flanking the serrations
249. Form of enamel wrinkles (Reductive coding of Benson, 2009 #95; also Brusatte et al., 2007; originally in Currie and Carpenter, 2000 #42)
0 bands extending across labial and lingual tooth surfaces
1 adjacent to carinae but do not extend across labial and lingual tooth surfaces
250. Vertical striations of enamel of tooth crowns (Benson, 2009 #93; also Sereno et al., 1998 #18; originally mentioned in Charig and Milner, 1997)
0 absent
1 present
251. Axial diapophyses (Benson, 2009 #104; also Carrano et al., 2002 #70, Holtz, 1994 #10; originally mentioned in Rowe, 1989)
0 moderate
1 reduced or absent
252. Axial parapophyses (Benson, 2009 #105; also Carrano et al., 2002 #70, Holtz, 1994 #11; originally mentioned in Rowe, 1989)
0 prominent or moderate
1 reduced or absent
253. Axial neural spine (Rauhut 2003, #93. Norell et al., 2001; Kirkland et al., 2005 #94)
0 flared transversely and sheet-like
1 compressed mediolaterally, anteroposteriorly reduced, and rodlike
254. Epipophyses on axis (Rauhut 2003, #92. Norell et al., 2001; Kirkland et al., 2005 #93)
0 absent
1 present
255. Form of axial epipophyses (Reductive coding of Rauhut 2003, #92 and Norell et al., 2001; Kirkland et al., 2005 #93 by JNC 02-19-2009)
0 present as small ridges
1 strongly pronounced (overhanging the zygapophyses)
256. Pleurocoel in axis (Rauhut, 2003, #91, also Benson, 2009 #102)
0 absent
1 present
257. Number of cervical vertebrae (Norell et al., 2001; Kirkland et al., 2005 #92, state 1 edited by JNC)

0 10
1 More than 10
258. Pleurocoels in cervical vertebrae (Rauhut, 2003, #88)
0 absent
1 present
259. Number of pleurocoels in cervicals (Rauhut, 2003, #89, state 2 added from Benson, 2009 #111; also Norell et al., 2001; Kirkland et al., 2005 #100 (with states reversed), Holtz, 1994 #4; originally mentioned in Rowe and Gauthier, 1990, also Benson, 2009 #111)
0 one
1 two
260. Arrangement of two foramina in cortical surface of cervical centra (Modified from Benson, 2009 #111)
0 one in anterior half of lateral surface, one in posterior half
1 both foramina in anterior half
261. Pleurocoels developed as (Rauhut, 2003, #90)
0 deep depressions
1 foramina
262. Interior pneumatic spaces in cervicals (Rauhut, 2003, #96; Character states changed to avoid redundancy)
0 Structure camerate (few chambers)
1 Structure camellate (many chambers separated by delicate lamellae)
263. Ventral surface of anterior cervicals (Rauhut, 2003, #97, Zanno et al., 2009, #269, with state (1) recoded as state (2))
0 keeled
1 smooth
2 ventral depression
264. Posterolateral crests on lateral surfaces of cervical centra (Rauhut, 2003, #98, this character is the same as Zanno et al., 2009 #270 and additional taxa were scored from that matrix)
0 absent
1 present
265. Anterior cervical centra length (Zanno et al., 2009, #268)
0 less than twice transverse centrum width
1 between two and three time transverse width
2 three to five times transverse width
266. Anterior articular facet of anterior cervical vertebrae (Rauhut, 2003, #101; also Norell et al., 2001; Kirkland et al., 2005 #98, with state 0 recoded as 0, and state 1 recoded as 2.)
0 approximately as high as wide or higher
1 significantly wider than high
2 wider than high and higher laterally than medially (kidney-shaped), with neural canal emarginating dorsal aspect
267. Anterior cervical centra relative length (Norell et al., 2001; Kirkland et al., 2005 #96)
0 level with or shorter than posterior extent of neural arch
1 centra extending beyond posterior limit of neural arch
268. Articulation surfaces of cervical centra (Rauhut, 2003, #95; also Norell et al., 2001; Kirkland et al., 2005 #101)
0 amphi- to platycoelus
1 opisthocoelus
2 heterocoelus (state added from Turner et al 2007)
269. Carotid process on posterior cervical centra (Norell et al., 2001; Kirkland et al., 2005 #97)
0 absent
1 present
270. Epipophyses in anterior cervical vertebrae (Rauhut, 2003 #102 reductive coding by JNC, also Norell et al., 2001; Kirkland et al., 2005 #25, with Kirkland state 0 = Rauhut state 2 and Kirkland state 1 = Rauhut 1; originally in Gauthier 1986)
0 absent or poorly developed
1 well-developed
271. Form of well-developed cervical epipophyses (Rauhut, 2003 #102 reductive coding by JNC)
0 proximal to postzygapophyseal facets
1 strongly overhanging postzygapophyseal facets
272. Prezygapophyseal-epipophyseal lamina on dorsal surface of neural arch (Carrano and

Sampson, 2008 #79)

0 absent or poorly developed

1 extending anteriorly from epipophysis as a mediolaterally thin ridge that separates dorsal surface of diapophysis from rest of dorsal neural arch

273. Postzygapophyses of cervical vertebrae 2-4 (JC 2008-09-17 personal observation)

0 well-separated, or connected only at the base (JC 2008-09-17 personal observation)

1 medially connected along their entire length by a intrazygapophyseal lamina that is dorsally concave for attachment of the interspinous ligaments

274. Cervical neural spines (Norell et al., 2001; Kirkland et al., 2005 #99with state 2 added)

0 anteroposteriorly long

1 anteroposteriorly short and centered on neural arch, giving arch an "X" shape in dorsal view

2 extremely short anteroposteriorly, less than 1/3 length of neural arch

275. Cervical neural spine height (Zanno et al., 2009, #271)

0 dorsoventrally tall, subequal to or exceeding height of neural arch from centrum to base of neural spine

1 moderate, less than neural arch height

2 strongly reduced, less than half height of neural arch (not including spine itself)

276. Prezygapophyses in anterior cervicals (Rauhut, 2003, #99; originally in Makovicky 1995, also Benson, 2009 #110)

0 transverse distance between prezygapophyses less than width of neural canal

1 prezygapophyses situated lateral to the neural canal

277. Prezygapophyses in anterior postaxial cervicals (Rauhut, 2003, #100; Originally in Gauthier, 1986)

0 straight

1 anteroposteriorly convex, flexed ventrally anteriorly

278. Cervical neural arches, posterior surface above neural canal

[New character. In all alvarezsauroids where the condition is observable, the posterior surface of the cervical neural arches immediately lateral and dorsal to the neural canal bears several deep fossae, which are readily seen in posterior view. This feature is variably present in many other theropods, e.g., ceratosaurs.]

0 without mutiple deep fossae

1 with multiple deep fossae surrounding neural canal

279. Postzygapophyses of posterior cervical vertebrae

[New character. This refers to the direction of the main axis of the postzygapophyses, not the midline web of bone that may or may not connect them.]

0 diverge only weakly laterally

1 diverge strongly laterally

280. Pneumaticity of dorsal neural arches (Benson et al., 2009 #229)

0 absent to moderate

1 extreme

281. Hypapophyses in anterior dorsals (Rauhut, 2003, #107; also Norell et al., 2001; Kirkland et al., 2005 #102)

0 absent or poorly developed

1 pronounced

282. Pleurocoels in dorsal vertebrae (Rauhut, 2003, #106; additional taxa coded from Benson, 2009 #115)

0 absent

1 present in anterior dorsals ('pectorals')

2 present in all dorsals

283. Dorsal centra articular surfaces (Taken from Longrich and Currie, 2008; originally in Perle et al 1993; states one and two added in this analysis)

0 amphiplatyan

1 some opisthocoelous

2 most opisthocoelous

284. Ventral keel in anterior dorsals (Rauhut, 2003, #108, also Benson, 2009 #116)

0 absent or very poorly developed

1 pronounced

285. Shape of dorsal centra in anterior view (Rauhut, 2003, #105)

0 subcircular or oval

1 significantly wider than high
2 triangular

286. Dorsal vertebrae, size of neural canal
[New character. In most theropods, the neural canal in the dorsal vertebrae is considerably smaller than the posterior articular facet of the corresponding centrum. Uniquely in parvicursorine alvarezsauroids, the neural canal is much larger in diameter than the posterior articular facet. This has long been recognized in alvarezsaurs (see Chiappe, 2002), but has not been codified as a character before.]
0 small, much smaller than posterior articular facet
1 large, subequal to posterior articular facet

287. Posterior dorsal vertebrae (Rauhut, 2003, #112)
0 strongly shortened, centra much shorter than high
1 relatively short, centra approximately as high as long, or only slightly longer
2 significantly elongated, much longer than high

288. Posterior dorsal vertebrae, basal webbing of neural spines (Benson, 2009 #123; also Sereno et al., 1998 #24; originally mentioned in Charig and Milner, 1997)
0 absent
1 present

289. Posterior dorsal vertebrae, orientation of neural spines (Benson, 2009 #124; originally in Harris, 1998 #68)
0 vertically or posteriorly
1 anteriorly

290. Anterior dorsal vertebrae height of prezygadiapophyseal lamina (Zanno et al., 2009, #272)
0 less than or subequal to height of centrum
1 hypaxially inflated, height significantly greater than centrum height

291. Dorsal vertebrae neural arches, anterior neural pedicle
[New character. See #292 explanation below]
0 without tubercle at base
1 pronounced tubercle at base

292. Dorsal vertebrae neural arches, posterior neural pedicle
[New character. In *Haplocheirus*, the base of the anterior neural pedicle, immediately dorsal to the centrum contact, bears a small tubercle that projects anteriorly. A similar tubercle is present on the posterior neural pedicle in *Xiyunykus* and *Patagonykus*, although these taxa lack the anterior tubercle. In the readily observable dorsal vertebral neural arches of *Haplocheirus*, this posterior tubercle isn't present, but it is possible that the more poorly preserved neural arches did bear this feature.]
0 without tubercle at base
1 pronounced tubercle at base

293. Anterior dorsal vertebrae, anterior and posterior infrazygapophyseal fossae (Zanno et al., 2009, #274)
0 single
1 with one or more accessory centrodiapophyseal laminae dividing fossa into multiple chambers

294. Transverse processes of anterior dorsal vertebrae (Zanno et al., 2009, #275)
0 subhorizontal to vertically inclined
1 pendant

295. Parapophyseal facets of anterior dorsal vertebrae (Zanno et al., 2009)
0 moderate in size (less than half height of centrum)
1 hypertrophied (greater than two thirds centrum height)

296. Hyposphene-hypantrum articulation in dorsal vertebrae (Rauhut, 2003, #103; also Norell et al., 2001; Kirkland et al., 2005 #104with state 1 and 2 scored as 1)
0 absent
1 present

297. Step-like ridge lateral to hyposphene running posterodorsally from the dorsal border of the neural canal to the posterior edge of the postzgyapophyses of dorsal vertebrae (visible in lateral view) (Benson, 2009 #118; also Smith et al., 2007 #174; originally mentioned in Rauhut, 2005)
0 absent
1 present

298. Postzygapophyses of the dorsal vertebrae in posterior view (Benson et al., 2009 #214)
0 without lateral flanges

1 with lateral, small, flange-like lateral extensions of postzygapophyseal facets

299. Postzygapophyses of dorsal vertebrae (Norell et al., 2001; Kirkland et al., 2005 #105; additional taxa coded from Benson, 2009 #117)

0 abutting one another above neural canal, opposite hyposphenes meet to form lamina

1 zygapophyses placed lateral to neural canal and separated by groove for interspinous ligaments, hyposphenes separated

300. Neural spines on posterior dorsal vertebrae in lateral view (originally in Chen et al 1998; Norell et al., 2001; Kirkland et al., 2005 #206; also Rauhut, 2003, #110)

0 rectangular or square

1 anteroposteriorly expanded distally, fan-shaped

301. Neural spines of dorsal vertebrae in dorsal view (Norell et al., 2001; Kirkland et al., 2005 #108)

0 not expanded distally

1 expanded laterally in dorsal view to form "spine table"

302. Dorsal vertebrae neural arches, spinopostzygapophyseal lamina

[New character. In *Bonapartenykus*, the spinopostzygapophyseal laminae terminate abruptly on the dorsal surface of the postzygapophyses, leaving a steep demarcation of the contact. In *Haplocheirus* and in parvicursorines, this feature is absent, but its distribution is unclear in transitional alvarezsaurids because of poor preservation.]

0 grades smoothly into postzygapophyseal dorsal surface or poorly developed

1 well developed and terminates abruptly on dorsal surface of postzygapophysis

303. Middle dorsal vertebrae, anterior centrodiapophyseal lamina (connects the parapophysis and the diapophysis)

[New character.Within alvarezsauroids, this feature is present in *Xiyunykus* and *Patagonykus*, but it is variably present in many other theropods, including some Oviraptorosaurs, Dromaeosaurs, and non-coelurosaurian tetanurans.]

0 absent or poorly developed

1 well developed

304. Scars for interspinous ligaments (Norell et al., 2001; Kirkland et al., 2005 #109)

0 terminate at apex of neural spine in dorsal vertebrae

1 terminate below apex of neural spine

305. Neural spines of posterior dorsals (Rauhut, 2003, #109)

0 broadly rectangular and approximately as dorsoventrally high as anteroposteriorly long

1 high rectangular, significantly dorsoventrally higher than anteroposteriorly long

306. Hook-like extension on anterior end of dorsal neural spines in lateral view (Adopted from Peyer, 2006: She considers this character present in *Dilophosaurus*, *Ornitholestes*, *Huaxiagnathus*, *Scipionyx* and *Sinosauropteryx*)

0 absent

1 present (with associated depression immediately caudal to the projection for spinous ligament attachment)

307. Parapophyses of posterior dorsal vertebrae (Norell et al., 2001; Kirkland et al., 2005 #103, also Benson, 2009 #121)

0 flush with neural arch

1 distinctly projected on pedicels

308. Parapophyses in posteriormost dorsals (Rauhut 2003, #111; originally in Makovicky 1995)

0 on same level as transverse process

1 distinctly below transverse process

309. Transverse processes of anterior dorsal vertebrae (Norell et al., 2001; Kirkland et al., 2005 #107, slight rewording by JNC to specify direction for "thin" and "long", and to remove "only slightly inclined", which refers to something separate from shape)

0 proximodistally long and anteroposteriorly thin

1 proximodistally short, anteroposteriorly wide

310. Notarium of dorsal vertebrae

0 absent

1 present

311. Number of sacral vertebrae (Norell et al., 2001; Kirkland et al., 2005 #110; also Rauhut, 2003 #113, Forster, 1999 #59; originally in Holtz, 1994 #121; additional taxa scored from Benson, 2009 #125)

0 two

1 three

2 four
3 five
4 six
5 seven
6 eight
7 nine or more

312. Pleurocoels in centra of sacral vertebrae (Norell et al., 2001; Kirkland et al., 2005 #113; also Rauhut, 2003 #115; additional taxa coded from Benson, 2009 #126)
0 absent
1 present on anterior sacrals only
2 present on all sacrals

313. Anteriormost sacral centrum, ventral surface
[In *Parvicursor*, and likely in most parvicursorine alvarezsaurods, the ventral surface of the anteriormost sacral centrum has a V-shaped cross section with a deep ventral keel. This feature is not present in the non-parvicursorine alvarezsaurid *Alvarezsaurus* but its distribution amongst other alvarezsaurs and theropods is poorly known]
0 rounded, convex
1 mediolaterally constricted and forms a keel

314. Ventral surface of posterior sacral centra (after Novas, 1997; Norell et al., 2001; Kirkland et al., 2005 #112; Rauhut, 2003, #114)
0 gently rounded, convex
1 flattened ventrally, sometimes with shallow sulcus
2 centrum strongly constricted transversely, ventral surface keeled

315. Transverse dimensions of mid-sacral centra relative to other sacral centra (Benson et al., 2009 #231)
0 subequal
1 mediolaterally narrower
2 mediolaterally wider (State added by JNC 2011-09-26 to include the pattern seen in birds e.g., *Apsaravis*)

316. Sacral vertebrae (Norell et al., 2001; Kirkland et al., 2005 #111)
0 with unfused zygapophyses
1 with fused zygapophyses forming a sinuous ridge in dorsal view

317. Last sacral centrum (Norell et al., 2001; Kirkland et al., 2005 #114)
0 with flat posterior articulation surface
1 convex articulation surface

318. Fenestrae between neural spines of sacral vertebrae (Benson, 2009 #127)
0 present
1 absent

319. Sacral ribs (Rauhut, 2003, #116; originally in Rowe and Gauthier, 1990)
0 slender and well-separated
1 forming a more or less continuous sheet in ventral or dorsal view
2 very massive and strongly expanded

320. Sacral neural arch pneumaticity (Benson et al., 2009 #230)
0 absent to moderate
1 extreme

321. Number of caudal vertebrae (Norell et al., 2001; Kirkland et al., 2005 #121; also Rauhut, 2003 #117)
0 more than 40
1 25-40
2 fewer than 25

322. Pygostyle
0 absent
1 present, centra of distal caudal vertebrae fused

323. Pleurocoels in centra of anterior caudal vertebrae (Zanno et al., 2009, #279; originally in Sereno et al., 1996 #55; additional taxa coded from Benson, 2009 #129)
0 absent
1 present

324. Caudal centra (Scored from Longrich and Currie, 2008 #21; originally from Novas, 1996)
0 amphiplatyan
1 procoelus

325. Shape of anterior caudal centra in cross section (Rauhut, 2003 #127; also Norell et al., 2001; Kirkland et al., 2005 #117; state 2 from Gohlich and Chiappe 2006 #117)
0 oval
1 subrectangular and box-like
2 laterally compressed with a ventral keel
326. Ventral surface of anterior caudals (Rauhut, 2003 #120,121 modified; also Norell et al., 2001; Kirkland et al., 2005 #117, Brusatte et al., 2008; originally mentioned in Rowe and Gauthier, 1990; additional taxa and character states from Benson, 2009 #128)
0 rounded
1 with a distinct keel sometimes bearing a narrow, shallow groove on its midline
2 grooved
327. Relative length of distal caudal centra (Rauhut, 2003, #126)
0 significantly elongated in relation to centrum height
1 not elongated in relation to centrum height
328. Caudal vertebrae (Norell et al., 2001; Kirkland et al., 2005 #115)
0 with distinct transition point, from shorter centra with long transverse processes proximally to longer centra with small or no transverse processes distally
1 homogeneous in shape, with no transition point
329. Position of transition point (Turner, 2007 #116; Rauhut, 2003 #119)
0 distal to the tenth caudal vertebra
1 between the 7th and 10th caudal vertebrae
2 proximal to the 7th caudal vertebra
330. Location of transverse processes of proximal caudals (Longrich and Currie 2008 #22)
0 centrally positioned on centrum
1 anteriorly displaced
331. Centrodiapophyseal laminae of anterior caudal vertebrae (Benson et al., 2009 #215)
0 weak
1 prominent, as well developed as those of dorsal vertebrae
332. Neural spines on distal caudals (Norell et al., 2001; Kirkland et al., 2005 #119)
0 form a low ridge
1 spine absent
2 midline sulcus in center of the neural arch
333. Neural spines of caudal vertebrae (Norell et al., 2001; Kirkland et al., 2005 #118. The anterior spur of Rauhut, 2003 #125 is the same as an anterior ala.)
0 simple, undivided
1 separated into anterior and posterior alae throughout much of caudal sequence
334. Neural spines of mid-caudals (Rauhut, 2003, #124, also Benson, 2009 #131)
0 rod-like and posteriorly inclined
1 rod-like and vertical
2 subrectangular and sheet-like
335. Prezygapophyses of distal caudal vertebrae (Combination of Norell et al., 2001; Kirkland et al., 2005 #120 and Rauhut 2003, #122)
0 between 1/3 and whole centrum length
1 with extremely long extensions of the prezygapophyses (up to 10 vertebral segments in some taxa)
2 strongly reduced as in *Archaeopteryx lithographica*
336. Anterior margin of neural spines of anterior mid-caudal vertebrae (Rauhut, 2003, #123, also Benson, 2009 #130)
0 straight
1 with distinct kink, dorsal part of anterior margin more strongly inclined posteriorly than ventral part
337. Long, hair-like cervical ribs
0 absent
1 present
338. Shaft of cervical ribs (Norell et al., 2001; Kirkland et al., 2005 #124)
0 slender and longer than vertebra to which they articulate
1 broad and shorter than vertebra
339. Posterior cervical ribs and centra (Benson, 2009 #114; originally in Gauthier, 1986 #55)
0 separate
1 fused

340. Ossified uncinate processes (Norell et al., 2001; Kirkland et al., 2005 #125)
0 absent
1 present
341. Ossified sternal ribs
0 absent
1 present
342. Lateral gastral segment (Norell et al., 2001; Kirkland et al., 2005 #127)
0 shorter than medial one in each arch
1 distal segment longer than proximal segment
343. Cranial process at base of chevrons (Rauhut, 2003, #128, also Benson, 2009 #133)
0 absent
1 present
344. Proximal surface of chevrons (Benson, 2009 #134)
0 distinct transverse ridge dividing surface into anterior and posterior facets
1 no ridge, low mounds may be present laterally
345. Proximal end of chevrons of proximal caudals (Norell et al., 2001; Kirkland et al., 2005 #122)
0 short anteroposteriorly, shaft cylindrical
1 proximal end elongate anteroposteriorly, flattened and plate-like
346. Mid-caudal chevrons (Rauhut, 2003, #130; originally Sereno et al 1996, also Benson, 2009 #135)
0 rod-like or only slightly expanded ventrally
1 L-shaped
347. Distal chevrons (Rauhut, 2003, #129)
0 rod-like or L-shaped
1 skid-like
348. Distal caudal chevrons (Norell et al., 2001; Kirkland et al., 2005 #123)
0 simple
1 anteriorly bifurcate
2 bifurcate at both ends
349. Ossified sternal plates (Norell et al., 2001; Kirkland et al., 2005 #128)
0 separate in adults
1 fused
350. Sternum (Norell et al., 2001; Kirkland et al., 2005 #129)
0 without distinct lateral xiphoid process posterior to costal margin
1 with lateral xiphoid process
351. Ventral keel on sternum
0 absent
1 present
352. Midline groove on sternal keel
[New character. Described by Xu et al 2013.]
0 absent
1 present
353. Anterior edge of sternum (Norell et al., 2001; Kirkland et al., 2005 #130)
0 grooved for reception of coracoids
1 without grooves
354. Articular facet of coracoid on sternum (conditions may be determined by the articular facet on coracoid in taxa without ossified sternum (Norell et al., 2001; Kirkland et al., 2005 #131; after Xu et al. 1999)
0 anterolateral or more lateral than anterior
1 almost anterior
355. Furcula (Rauhut, 2003, #131)
0 absent
1 present
356. Furcula shape (Zanno et al., 2009, #281)
0 v-shaped
1 u-shaped, with bowed epicleidea
357. Hypocleidium on furcula (Norell et al., 2001; Kirkland et al., 2005 #132)
0 absent
1 present
358. Coracoid in lateral view (Norell et al., 2001; Kirkland et al., 2005 #136; also Benson, 2009

#140; also Rauhut, 2003, #138; originally Gauthier 1986)
0 subcircular, with low ventral blade and no or small posterior process
1 shallow ventral blade with elongate posterior process
2 subquadrangular with extensive ventral blade
3 strut-like, very tall ventral blade with little or no posterior process
359. Posterior edge of coracoid (Norell et al., 2001; Kirkland et al., 2005 #218)
0 not or shallowly indented below glenoid
1 posterior edge of coracoid deeply notched just ventral to glenoid, glenoid lip everted
360. External surface of coracoid ventral to glenoid fossa and along dorsal margin of posterventral blade (Norell et al., 2001; Kirkland et al., 2005 , #134)
0 unexpanded
1 expanded, forms triangular subglenoid fossa bounded laterally by coracoid tuber
361. Coracoid tubercle (Reductive coding of Benson, 2009 #141; modified by JNC 2012-1-12 to recognize stages in diminishing tubercle in alvarezsauroids.)
0 absent
1 weakly developed, only a low structure
2 strongly developed
362. Coracoid tubercle form (Choiniere et al., 2012 #351)
0 anteroposteriorly short, mound-like
1 anteroposteriorly elongated, ridge-like
363. Strong lateral ridge on coracoid extending posteriorly from coracoid tuber along posteroventral process (Agnolin et al., 2011 #422)

0 absent
1 present
364. Coracoid, ventral half of lateral surface (Angolin et al., 2011 #423)
0 bone smooth or only lightly textured
1 cortical bone surface rugose and heavily textured
365. Coracoid, anterior view
[New character. Added with reference to Angolin et al., 2011, who discuss this feature in *Bonapartenykus*. This feature is also present in *Patagonykus*.]
0 dorsal and ventral portions coplanar or subcoplanar
1 ventral portion directed ventromedially at obtuse angle to dorsal portion
366. Coracoid foramen
0 present
1 absent
367. Scapula shape (Rauhut, 2003 #132)
0 short and broad (ratio length/minimal height of shaft <9)
1 slender and elongate (ratio >10)
368. Scapulocoracoid junction anterior surface (Benson, 2009 #139; also Tykoski and Rowe, 2004 #108; originally in Harris, 1998 #84)
0 indented or notched between the scapular acromial process and the coracoid suture
1 smoothly curved and uninterrupted across the contact between the scapula and coracoid
369. Acromion margin of scapula (Norell et al., 2001; Kirkland et al., 2005 #133; also Rauhut 2003, #134; Zanno et al., 2009)
0 continuous with blade
1 anterior edge enlarged and projects anteriorly at approximately a right angle
370. Notch on posterior margin of scapular blade immediately dorsal to glenoid lip
[New character. This character is describe but not used phylogenetically in Agnolin et al., 2011.]
0 absent
1 present, associated with a small posterior protuberance
371. Posterior surface of scapular blade distal to glenoid buttress
0 without deep groove
1 with deep groove
372. Flange on supraglenoid buttress on scapula (see Nicholls and Russell, 1985) (Norell et al., 2001; Kirkland et al., 2005 #220)
0 absent
1 present
373. Tubercle on posterior surface of scapula dorsal to glenoid
[New character. Added to homologize condition in alvarezsaurids. This feature is easily

distinguished from the supraglenoid buttress of Nichols and Russel 1985, which projects laterally and forms a portion of the anterior border of the glenoid facet of the scapula.]

0 absent
1 present

374. Distal end of scapula (Rauhut, 2003, #133; originally in Gauthier, 1986)

0 expanded
1 not expanded

375. Glenoid fossa (Norell et al., 2001; Kirkland et al., 2005 #138; also Rauhut 2003 #135)

0 faces posteriorly or posterolaterally
1 faces laterally

376. Scapula and coracoid (Norell et al., 2001; Kirkland et al., 2005 #135)

0 separate
1 fused into scapulocoracoid

377. Scapula and coracoid orientation

0 continuous arc in posterior and anterior views
1 coracoid inflected medially, scapulocoracoid L shaped in anterior or posterior view

378. Scapula length (Turner et al 2007 #139)

0 longer than humerus
1 shorter than humerus

379. Deep fossa on anterior side of lateral surface of proximal end of scapula immediately dorsal to scapulocoracoid junction

0 absent
1 present

380. Deltopectoral crest length (Zanno et al., 2009, #286; modified from Clark et al., 2004; originally in Sereno et al., 1998 #3; additional taxa coded from Benson, 2009 #144)

0 less than one quarter humeral length
1 approximately one third humeral length
2 greater than one half humeral length

381. Deltopectoral crest (Norell et al., 2001; Kirkland et al., 2005 #140)

0 large and distinct, proximal end of humerus quadrangular in anterior view
1 less pronounced, forming an arc rather than being quadrangular
2 very weakly developed, proximal end of humerus with rounded edges
3 extremely long (as in *Shuvuuia* and *Mononykus*)

382. Deltopectoral crest orientation (Benson, 2009 #145; originally in Sereno et al., 1998 #31)

0 longitudinal
1 oblique distolaterally and distal end of crest oriented laterally rather than anteriorly from the humeral shaft

383. Lateral surface of distal end of deltopectoral crest (Turner, 2007 #141)

0 smooth
1 with distinct muscle scar near lateral edge along distal end of crest for insertion of biceps muscle

384. Ratio femur/humerus (Rauhut, 2003, #139; additional taxa coded from Benson, 2009 #142)

0 more than 2.5
1 between 1.2 and 2.2
2 less than 1

385. Outline of proximal articular facet of humerus (Rauhut, 2003, #140)

0 broadly oval (more than twice as broad transversely than anteroposteriorly)
1 distinctly rounded, often globular (less than twice as broad anteroposteriorly than transversely)

386. Internal tuberosity of humerus (Chiappe et al 2003; Novas 1996)

0 small and confluent with humeral head
1 offset from humeral head by distinct notch, often projects proximally above humeral head
2 hypertrophied but not distinct from humeral head (as in Suchomimus)

387. Shape of internal tuberosity on humerus in anterior view (Rauhut, 2003, #141)

0 triangular, often rounded
1 rectangular

388. Humerus in lateral view (Rauhut, 2003, #143; originally in Holtz, 1994, also Benson, 2009 #143)

0 sigmoidal
1 straight

389. Transverse width of distal humerus (Zanno et al., 2009, #293)
0 greater than 2.7 times shaft width
1 between 2 and 2.5 times humeral shaft width
2 less than twice shaft width

390. Ectepicondyle of humerus (lateral epicondyle) (Choiniere et al., 2010)

0 small, often rectangular and does not form articular surface
1 large, rounded and forms articular surface

391. Ectepicondyle of humerus
[New character. In alvarezsaurids including *Patagonykus* and more crownward taxa, there is a distinct notch between the distal end of the humerus and the ectepicondyle.]
0 confluent with humeral distal end
1 set off from humeral distal end by a distinct notch

392. Entepicondyle of humerus (medial epicondyle) (Norell et al., 2001; Kirkland et al., 2005 #225 modified by JC 2008-07-28; additional taxa coded from Benson, 2009 #147 ("ulnar epicondyle"))
0 absent or small and tabular
1 large, projects medially from ulnar condyle as a distinct process and is distally separated from ulnar condyle by a groove

393. Distal humeral condyles (Norell et al., 2001; Kirkland et al., 2005 #226)
0 primarily developed on distal end of humerus, but may also have some articular surface extending to anterior edge
1 limited to anterior surface, condylar surfaces not present on distal end

394. Ulnar shaft (Discussed in Martinez and Novas, 2006, coded by JNC 2-16-2009)
0 straight
1 bowed

395. Olecranon process of ulna (Rauhut, 2003, #144, modified by JNC; also Norell et al., 2001; Kirkland et al., 2005 #142; originally in Novas, 1998)
0 absent or weakly developed
1 well-developed
2 hypertrophied

396. Shape of olecranon process (Benson et al., 2009 #219; also Smith et al., 2009 #348; originally mentioned in Calvo et al., 2004)
0 transversely broad
1 mediolaterally thin, blade-like

397. Crest extending along posterior surface of ulnar shaft from olecranon process (Benson et al., 2009 #225)
0 absent
1 present

398. Proximal surface of ulna (Norell et al., 2001; Kirkland et al., 2005 #144)
0 single continuous articular facet
1 divided into two distinct fossae

399. Proximal end of the ulna in proximal view (Wording modified from Benson, 2009 #149)
0 without extensive coronoid process and radial process on radial side of proximal end (without extensive coronoid process on radial side of proximal end)
1 coronoid and radial processes large (coronoid process large, extends at right angle to olecranon)

400. Distal articular surface of ulna (Norell et al., 2001; Kirkland et al., 2005 #143)
0 flat
1 convex, semilunate surface

401. Distal condyle articular surface of ulna (Longrich and Currie, 2008 #33)
0 unexpanded or spatulate, articular surface limited to distal end
1 bulbous, trochlear articular surface extends onto dorsal surface of ulna

402. Radius length (Rauhut, 2003, #145)
0 more than half the length of humerus
1 less than half the length of humerus

403. Radial shaft (Benson, 2009 #151)
0 straight
1 bowed laterally

404. Radius and ulna (Norell et al., 2001; Kirkland et al., 2005 #211)

0 well-separated
1 with distinct adherence or syndesmosis distally

405. Ossified carpals
0 absent
1 present (Personal observation, but may appear in some ceratosaur matrices)

406. Lateral proximal carpal (ulnare?) (Norell et al., 2001; Kirkland et al., 2005 #145)
0 quadrangular
1 triangular in proximal view

407. Trochlea on the proximal surface of distal carpal 1 (Modified from Rauhut 2003, #146)
0 absent
1 present

408. Two distal carpals (Norell et al., 2001; Kirkland et al., 2005 #146)
0 in contact with metacarpals, one covering the base of Mc I (and perhaps contacting Mc II) , the other covering the base of Mc II
1 two distal carpals not present, single distal carpal capping Mc I and II

409. Distal carpals (Norell et al., 2001; Kirkland et al., 2005 #147)
0 not fused to metacarpals
1 fused to metacarpals, forming carpometacarpus

410. Rectangular buttress on ventrolateral surface of proximal end of Mc I (Originally in Russell and Dong, 1993; coded from Zanno et al., 2009, #295)
0 absent
1 present

411. Length of Mc I (Derived from Norell et al., 2001; Kirkland et al., 2005 #149,with states reversed)
0 approximately half the length of Mc II
1 subequal in length to Mc II

412. Shape of Mc I (Rauhut 2003, #164. Also Norell et al., 2001; Kirkland et al., 2005 #149)
0 significantly longer than broad
1 very stout, approximately as long as broad

413. Contact between Mc I and Mc II (Rauhut, 2003, #148, also Benson, 2009 #156)
0 metacarpals contact each other at their bases only
1 Mc I closely appressed to Mc II, at least the proximal half of McI flattened

414. Medial tab on proximal end of Mc I ('proximo-radial process' of Gishlick and Gauthier, 2007) (Mentioned in Gishlick and Gauthier, 2007)
0 absent or poorly developed
1 well-developed

415. Proximomedial tab projects
[New character. Added to differentiate between *Haplocheirus*, where this process is present but not hypertrophied, and other alvarezsaurs, where the process is hypertrophied.]
0 mostly medially, does not extend proximally beyond proximal articular surface
1 far proximally, well beyond proximal articular surface

416. Distal end of Mc I (Rauhut, 2003, #149)
0 condyles more or less symmetrical
1 condyles strongly asymmetrical, the medial condyle being positioned more proximally than the lateral

417. Distal articular end of metacarpal I (Norell et al., 2001; Kirkland et al., 2005 #210; redefined by JC 2008-08-09)
0 ginglymoid
1 rounded and smooth

418. Distal condyles of Mc I
[New character. In most alvarezsauroids, including *Haplocheirus*, *Tugulusaurus*, and *Bannykus*, but notably not in Ceratonykus, the collateral ligament pits on the lateral and medial surfaces of the distal condyles of metacarpal I are absent or reduced sto shallow concave areas]
0 with collateral ligament fossae
1 collateral ligament fossae absent

419. Medial side of Mc II (Rauhut, 2003, #150)
0 expanded proximally
1 not expanded

420. Shaft of Mc II
[New character. In *Haplocheirus*, the shaft of Mc II is straight, but in the alvarezsaurids *Bannykus*

and *Linhenykus*, the shaft of Mc II is conspicuously bowed. The hand of other non-parvircursorine alvarezsaurids is incompletely known, but it appears that this may be a transient feature in the reduction of the alvarezsaur manus.]

0 straight
1 curved, medial surface is concave, lateral surface is bowed
2 curved, medial surface is convex, lateral surface is concave

421. Distal articular end of McII

0 ginglymoid
1 without ginglymus

422. Shaft of Mc III (Rauhut, 2003, #151)

0 subequal in width to Mc II
1 considerably more slender than Mc II (less than 70% of the width of Mc II)

423. Proximal articular end of Mc III (Rauhut, 2003, #152; originally Gauthier 1986)

0 expanded and similar in width to Mc I and II
1 not expanded, very slender when compared to Mc I and II

424. Proximal articular surface of Mc III

[New character. Added to differentiate the flat proximal condition in *Haplocheirus* and most other theropods from the cuplike condition in parvicursorine alvarezsauroids.]

0 flat or slightly convex
1 deeply concave and cuplike

425. Length of Mc III

0 much longer than Mc I
1 subequal in length to Mc I
2 much shorter than Mc I

426. Proximal outline of Mc III (Rauhut, 2003, #156)

0 subrectangular
1 triangular, apex dorsal

427. Shaft of Mc III (Rauhut, 2003, #157)

0 straight
1 bowed laterally

428. Extensor pits on the dorsal surface of the distal end of metacarpals (Rauhut, 2003, #155)

0 absent or poorly developed
1 deep, well-developed

429. Number of manual digits with one or more phalanges (Rauhut, 2003 #153; modified by JNC)

0 five
1 four
2 three
3 two (as in *Tyrannosaurus rex*)

430. Number of metacarpals

0 five
1 four
2 three

431. Paired flexor processes on proximal ventral surfaces of proximalmost phalanges (From Sereno, 2001)

0 absent
1 present

432. Lateral and medial surfaces of phalanx I-1

0 face predominantly medially and laterally, either flat or convex
1 face dorsolaterally and dorsomedially and are shallowly concave

433. Flexor surface of manual phalanx I-1 (Choiniere et al., 2010; mentioned in Chiappe et al., 2003)

0 convex or flat
1 concave, 'axial furrow' along proximodistal axis

434. Shaft diameter of phalanx I-1 (Norell et al., 2001; Kirkland et al., 2005 #207)

0 less than shaft diameter of radius
1 greater than shaft diameter of radius

435. Proximodistal length of phalanx I-1/length of Mc I (Rauhut, 2003, #158)

0 1 or less
1 between 1 and 1.5
2 more than 1.5

436. Penultimate phalanx of the second finger (Rauhut, 2003, #159)
0 shorter than first phalanx
1 longer than first phalanx

437. Penultimate phalanx of the third finger (Rauhut, 2003, #160)
0 as long as, or shorter than, more proximal phalanges
1 longer than each of the more proximal phalanges
2 longer than both proximal phalanges taken together

438. Length of third manual digit (Rauhut 2003, #154; originally from Gauthier 1986)
0 longer than second finger
1 shorter than or equal in length to second finger

439. Proximal articular surface of manual ungual I-2 (Longrich and Currie, 2008 #46)
0 dorsoventrally much taller than mediolaterally wide
1 mediolaterally as broad as tall

440. Unguals on all manual digits (Norell et al., 2001; Kirkland et al., 2005 #152)
0 generally similar in size
1 digit I bearing large ungual and unguals of other digits distinctly smaller

441. Transverse ridge immediately dorsal to the articulating surface of unguals (Norell et al., 2001; Kirkland et al., 2005 #153 use the term "lip" to describe this feature; also Rauhut, 2003, #162; originally from Currie and Russell 1988)
0 absent
1 present

442. Flexor tubercle placement (Zhang et al 2008 #151)
0 proximal
1 distal
2 absent

443. Curvature of ventral surface manual ungual I (Zhang et al 2008 #298)
0 strongly curved
1 weakly curved
2 straight

444. Curvature of ventral surface of manual unguals II and III (Zhang et al 2008 #299)
0 strongly curved
1 weakly curved
2 straight

445. Flexor tubercle size (Zhang et al 2008 #348)
0 large (> 1/3 articular facet height)
1 small (< 1/3 articular facet height)

446. Lateral grooves of manual ungual I-2 in ventral view (Longrich and Currie, 2008 #45)
0 unenclosed
1 proximal end of grooves partially enclosed by lateral notches
2 proximal end of grooves passes through foramena on ventral surface of ungual

447. Fusion of pelvic elements in adults (Benson, 2009 #160; originally mentioned in Rowe and Gauthier, 1990)
0 absent
1 present

448. Ilium (Rauhut, 2003, #166)
0 brachyiliac
1 dolichoiliac

449. Ilium pneumaticity (Benson et al., 2009 #217)
0 little or none
1 large external pneumatic foramina and internal spaces

450. Dorsal margin of ilium (Zanno et al., 2009, #307)
0 subhorizontal or gently inclined relative to axis of pubic and ischial contact
1 rises steeply as it extends anteriorly, at least 30 degree angle from the axis of the pubic and ischial contact

451. Ventral edge of anterior ala of ilium (Norell et al., 2001; Kirkland et al., 2005 #154 reductive coding by JNC; also Rauhut, 2003, #168, also Benson, 2009 #163)
0 straight or gently curved
1 ventral edge hooked anteriorly

452. Form of hook of preacetabular ala of ilium (Norell et al., 2001; Kirkland et al., 2005 #154 reductive coding by JNC; also Rauhut, 2003, #168, also Benson, 2009 #163)

0 weak
1 strong

453. Preacetabular part of ilium (Rauhut, 2003, #169; also Norell et al., 2001; Kirkland et al., 2005 #155)
0 significantly shorter than postacetabular part
1 subequal in length to postacetabular part
2 significantly longer than postacetabular process

454. Anterior rim of ilium (Rauhut 2003, #173. Norell et al., 2001; Kirkland et al., 2005 #156)
0 shallowly convex or straight
1 strongly convex or pointed anteriorly

455. Dorsally-positioned, anteriorly-concave notch on anterior rim of ilium
0 absent
1 present

456. Preacetabular part of ilium (height) (Rauhut, 2003, #170; state 2 added by JNC 2011-09-27 to reflect the dorsoventral expansion of the ilium in alvarezsaurs)
0 approximately as high as postacetabular part (excluding the ventral expansion)
1 significantly higher than postacetabular part
2 significantly lower than the postacetabular part

457. Cuppedicus fossa (Norell et al., 2001; Kirkland et al., 2005 #164)
0 absent
1 present

458. Form of cuppedicus fossa (Reductive coding of Norell et al., 2001; Kirkland et al., 2005 #164 by JNC 02-19-2009)
0 deep, ventrally concave
1 fossa shallow or flat, with no lateral overhang

459. Cuppedicus fossa position (Norell et al., 2001; Kirkland et al., 2005 #163)
0 ridge bounding fossa terminates rostral to acetabulum or curves ventrally onto anterior end of pubic peduncle
1 rim extends far posteriorly and is confluent or almost confluent with acetabular rim

460. Preacetabular portion of ilium (Norell et al., 2001; Kirkland et al., 2005 #229)
0 parasagittal
1 moderately laterally flaring

461. Brevis fossa shape (Rauhut 2003, #176; also Kirkland et at, #161, Sereno et al., 1994; originally mentioned in Molnar et al., 1990; additional taxa coded from Benson, 2009 #168)
0 shelf-like, narrow with subparallel margins
1 deeply concave, expanded posteriorly with lateral overhang

462. Brevis fossa lateral view (Turner et al., 2007 #217)
0 Poorly developed adjacent to ischial peduncle, without lateral overhang and medial edge of the brevis fossa is visible
1 well developed fossa along full length of postacetabular blade, lateral overhang extends along full length of fossa, medial edge of brevis fossa covered in lateral view

463. Medial brevis shelf (Longrich and Currie, 2008 #53)
0 strongly developed, projects medially
1 low ridge on medial surface of postacetabular ala

464. Shape of postacetabular ala of ilium in lateral view (Norell et al., 2001; Kirkland et al., 2005 #158; also Rauhut, 2003, #174)
0 squared
1 acuminate

465. Postacetabular ala of ilium in lateral view
[New character. Added to homologize the condition of the ilium in *Haplocheirus* and other alvarezsaurs (state 2). In some dromaeosaurs and some oviraptorosaurs, the ventral edge is concave but does not extend far ventrally.]
0 ventral edge flat
1 ventral edge concave
2 ventral edge concave and distal end extends ventrally below level of the ventral margin of the ischial peduncle

466. Articulation of iliac blades with sacrum (Rauhut, 2003, #171 in part)
0 vertical, well-separated above sacrum
1 strongly inclined mediodorsally, almost contacting each other or sacral neural spines at midline

467. Vertical ridge on iliac blade above acetabulum (Rauhut, 2003, #172, state 2 from Benson, 2009 #162; also Harris, 1998 #103; originally mentioned in Bonaparte et al., 1986; additional taxa coded from Benson, 2009 #162)

0 absent
1 low ridge with associated foramina
2 well-developed

468. Shape of pubic peduncle of ilium (Rauhut, 2003, #175)

0 transversely broad and roughly triangular in outline
1 anteroposteriorly elongated and narrow

469. Iliac pubic peduncle length relative to iliac ischial peduncle (Rauhut, 2003, #177, also Benson, 2009 #165; state 2 from Choiniere et al 2010, and was first mentioned in Martinelli and Vera, 2007)

0 significantly longer than ischial peduncle, ischial peduncle tapering ventrally and without clearly defined articular facet
1 subequal in length to ischial peduncle
2 anteroposteriorly shorter than the ischial peduncle

470. Articulation facet of pubic peduncle of ilium (Rauhut, 2003, #178; additional taxa scored from Benson, 2009 #169)

0 facing more ventrally than anteriorly, and without a pronounced kink
1 with pronounced kink and anterior part facing almost entirely anteriorly

471. Anterior margin of pubic peduncle (Rauhut, 2003, #179)

0 straight or convex
1 concave

472. Supraacetabular crest

0 absent
1 present

473. Form of supraacetabular crest (Norell et al., 2001; Kirkland et al., 2005 #157)

0 forms hood over femoral head
1 reduced, not forming hood

474. Antitrochanter posterior to acetabulum (Norell et al., 2001; Kirkland et al., 2005 #162with states reversed)

0 absent or poorly developed
1 prominent

475. Postacetabular blades of ilia in dorsal view (Norell et al., 2001; Kirkland et al., 2005 #159)

0 parallel
1 diverge posteriorly

476. Tuber along dorsal edge of ilium, dorsal or slightly posterior to acetabulum (Norell et al., 2001; Kirkland et al., 2005 #160)

0 absent
1 present

477. Dorsal margin of postacetabular ala in lateral view (Turner et al 2007 #226; originally in Novas, 2004)

0 convex or straight
1 concave, brevis shelf extends caudal to lateral ilium making it appear concave in lateral view

478. Caudal end of postacetabular ala in dorsal view (Turner et al 2007 #227; originally in Makovicky et al 2003)

0 rounded or squared in dorsal view
1 lobate, with brevis shelf extending caudally beyond caudal terminus of the postacetabular ala

479. Ilium and ischium articulation (Norell et al., 2001; Kirkland et al., 2005 #227; additional taxa scored from Benson, 2009 #179)

0 flat or slightly concavo-convex
1 with process projecting into socket in ischium

480. Pubic orientation (Norell et al., 2001; Kirkland et al., 2005 #175)

0 propubic
1 vertical (Acc. to Norell et al., 2001; Kirkland et al., 2005 #175)
2 moderately posteriorly oriented
3 opisthopubic

481. Prepubic tubercle on proximal end of pubis anterior to acetabular portion

[New character. In parvircursorine alvarezsauroids as well as in *Bonapartenykus*, the anterior margin of the dorsal portion of the pubis is anteriorly expanded into a tubercle]
0 absent
1 present

482. Strongly expanded pubic boot (Rauhut, 2003 #184; also coded with respect to Norell et al., 2001; Kirkland et al., 2005 #176, state 2 (no anterior or posterior projections); originally in Gauthier 1986)
0 absent
1 present

483. Pubic boot projects (Norell et al., 2001; Kirkland et al., 2005 #176; see also Rauhut, 2003 #184-187; additional taxa coded from Benson, 2009 #175)
0 anteriorly and posteriorly
1 with little or no anterior process
2 only expanded anteriorly

484. Ratio length of pubic boot to length of pubic shaft (Benson, 2009 #174; originally in Gauthier, 1986 #48)
0 less than 0.3
1 more than 0.5

485. Pubic boot outline, distal view
0 triangular
1 narrow, with subparallel margins

486. Pubic apron (Norell et al., 2001; Kirkland et al., 2005 #177; also see Rauhut, 2003 #167)
0 present
1 absent

487. Form of pubic apron (Norell et al., 2001; Kirkland et al., 2005 #177; also see Rauhut, 2003 #167)
0 extends medially from middle of cylindrical pubic shaft
1 shelf extends medially from anterior edge of anteroposteriorly flattened shaft

488. Pubic apron (Norell et al., 2001; Kirkland et al., 2005 #179)
0 about half of pubic shaft length
1 less than 1/3 of shaft length

489. Pubic apron (Rauhut, 2003, #182)
0 completely closed
1 with medial opening distally above the pubic boot

490. Pubic obturator foramen (Rauhut, 2003, #180; originally in Holtz 1994)
0 present
1 absent

491. Form of pubic obturator foramen (Rauhut, 2003, #180; originally in Holtz 1994, also Benson, 2009 #172)
0 completely enclosed
1 open ventrally (obturator notch)

492. Pubic fenestra below obturator foramen (Rauhut, 2003, #181)
0 absent
1 present

493. Pubic shafts in lateral view (Rauhut, 2003, #183; also Norell et al., 2001; Kirkland et al., 2005 #178; additional taxa coded from Benson, 2009 #172)
0 straight
1 anteriorly convex
2 anteriorly concave

494. Lateral face of pubic shafts (Turner et al 2007 #231; originally in Senter 2004)
0 smooth
1 with prominent lateral tubercle about halfway down the shaft

495. Length of Ischium (Norell et al., 2001; Kirkland et al., 2005 #171; also Rauhut, 2003, #191; originally from Gauthier 1986)
0 more than two-thirds pubis length
1 two thirds or less of pubic length

496. Obturator process of ischium (Norell et al., 2001; Kirkland et al., 2005 #167; also Rauhut, 2003, #189; reductive coding by JNC 2009-5-12)
0 absent
1 present

497. Position of obturator process (Reductive coding of Norell et al., 2001; Kirkland et al., 2005 #167; also Rauhut, 2003, #189by JNC 2009-05-12)
0 proximal in position
1 located near middle of ischiadic shaft
2 located at distal end of ischium
498. Ischial shaft (Turner et al 2007 #166; originally in Makovicky et al 2005)
0 Rodlike
1 anteroposteriorly wide and plate like
499. Lateral blade of ischium (Turner 2007 #168)
0 flat or laterally convex
1 laterally concave
2 with longitudinal ridge subdividing lateral surface into anterior (including obturator process) and posterior parts
500. Ischium, lateral view (Norell et al., 2001; Kirkland et al., 2005 #166 modified by JNC; additional taxa coded from Benson, 2009 #178)
0 straight
1 distally curved anteriorly
2 distally curved posteriorly
501. Ischium, anterior view (Separated from Norell et al., 2001; Kirkland et al., 2005 #166 by JNC)
0 straight
1 laterally convex
2 twisted at midshaft and with flexure of obturator process toward midline so that distal end is horizontal
3 laterally concave
502. Contact of obturator process of ischium (Norell et al., 2001; Kirkland et al., 2005 #168)
0 does not contact pubis
1 contacts pubis
503. Ventral notch at distal edge of ischial obturator process (Rauhut 2003, #190: changed wording and added word "shaft" to clarify; originally in Sereno et al 1996, also Benson, 2009 #182)
0 absent, grades smoothly into ischial shafts
1 present
504. Obturator process on ischium (Rauhut 2003, #188; also Norell et al., 2001; Kirkland et al., 2005 #169; also Benson, 2009 #181)
0 confluent with pubic peduncle
1 offset from pubic peduncle by a distinct notch
505. Morphology of offset triangular obturator process of ischium (Turner et al 2007 #234)
0 wide base along ischiac shaft, rostral process short
1 narrow base, rostral process elongate
506. Distal end of ischium (Rauhut 2003, #193; also Norell et al., 2001; Kirkland et al., 2005 #173)
0 strongly expanded, forming ischial "boot"
1 slightly expanded
2 tapering
507. Distal ends of ischia (Norell et al., 2001; Kirkland et al., 2005 #172)
0 form symphysis
1 approach one another but do not form symphysis
2 widely separated
508. Distally placed process on caudal margin of ischium (Turner et al 2007 #232; originally Forster et al 1998)
0 absent
1 present
509. Tubercle on anterior edge of ischium (Norell et al., 2001; Kirkland et al., 2005 #174)
0 absent
1 present
510. Posterior process (ischial tuberosity) on posteroproximal part of ischium (Rauhut, 2003, #192; Norell et al., 2001; Kirkland et al., 2005 #165)
0 absent
1 well-developed
511. Form of posteroproximal ischial process (ischial tuberosity) (Turner et al., 2007 #230)
0 small, tablike
1 large, proximodorsally hooked and separated from the iliac peduncle by a notch

512. Semicircular scar on posterior part of the proximal end of the ischium (Norell et al., 2001; Kirkland et al., 2005 #170)
0 absent
1 present
513. Femoral length (Zhang et al 2008 #309)
0 longer than tibia
1 shorter than tibia
514. Femoral head (Norell et al., 2001; Kirkland et al., 2005 #180; also Rauhut, 2003, #197; fovea ligamentum capitis of Baumel and Witmer, 1993:64))
0 without fovea capitalis
1 circular fovea present in center of medial surface of head
515. Oblique ligament groove on the posterior surface of femoral head (Originally in Rauhut 2003; additional taxa scored from Smith et al 2007 #296, also Benson, 2009 #189)
0 absent or very shallow
1 deep, bound medially by a well-developed posterior lip
516. Femoral head and greater trochanter (Rauhut, 2003, #194; Originally in Holtz 1994)
0 confluent with greater trochanter
1 separated from greater trochanter by a distinct cleft
517. Femoral head direction anteroposterior (Rauhut, 2003, #195)
0 directed anteromedially
1 directed strictly medially
518. Femoral head direction dorsoventral (Benson, 2009 #187; originally in Harris, 1998 #121)
0 ventromedial
1 horizontal
2 dorsomedial
519. Greater trochanter (Rauhut, 2003, #196)
0 anteroposteriorly narrow and narrowing from medial to lateral
1 anteroposteriorly expanded, forming a trochanteric crest
520. Lesser trochanter (Norell et al., 2001; Kirkland et al., 2005 #181)
0 separated from greater trochanter by a deep cleft
1 trochanters separated by small groove
2 completely fused (or absent) to form crista trochanteris
521. Lesser trochanter shape (Norell et al., 2001; Kirkland et al., 2005 #181)
0 alariform (Additional taxa coded from state 2 of Rauhut, 2003 #198 "broadened (wing-like)")
1 cylindrical in cross section
2 very short and ridge-like
522. Proximal extent of lesser trochanter (Reworded for clarity from Rauhut, 2003 #199)
0 at distal end of femoral head
1 more proximally placed, but distal to greater trochanter
2 as proximal or more proximal than greater trochanter
523. Accessory trochanteric crest on distal end of lesser trochanter (Norell et al., 2001; Kirkland et al., 2005 #185; additional taxa scored from Benson, 2009 #191; also Norell et al., 2001; Kirkland et al., 2005 #215 because it is coding the same thing)
0 absent
1 present
524. Posterolateral trochanter (Norell et al., 2001; Kirkland et al., 2005 #183; also Rauhut, 2003, #200; Turner et al 2007 call this the "lateral ridge")
0 absent or represented only by rugose area
1 posterior trochanter distinctly raised from shaft, mound-like
525. Fourth trochanter on femur (Norell et al., 2001; Kirkland et al., 2005 #184; also Rauhut, 2003, #201; originally Gauthier 1986, also Benson, 2009 #192)
0 present
1 absent
526. Broad groove on extensor surface of distal femur (Rauhut, 2003, #202; also Norell et al., 2001; Kirkland et al., 2005 #186; also Forster 1999; originally in Perez-Moreno et al., 1993 #42, also Benson, 2009 #193)
0 absent or poorly developed
1 well developed
527. Femoral medial epicondyle (medial distal crest, expanded medial lamella) (Benson, 2009

#195; also Carrano et al., 2002 #135; originally in Forster, 1999 #87)
0 stout ridge or absent
1 flange like, medially extensive
528. Popliteal fossa on distal end of femur (Norell et al., 2001; Kirkland et al., 2005 #187)
0 open distally
1 closed off distally by contact between distal condyles
529. Infrapopliteal ridge present posteriorly between medial condyle and crista tibiofibularis (Benson, 2009 #197; also Tykoski and Rowe, 2004 #155; originally in Hutchinson, 2001 #18)
0 absent
1 present
530. Distal end of femur (Rauhut, 2003, #203)
0 anteroposteriorly broad and distally flattened
1 less broad and well rounded
531. Lateral femoral distal condyle
0 distally rounded
1 distally conical
532. Distal projection of lateral femoral distal condyle (Benson, 2009 #196; originally in Brusatte and Sereno, 2008 #84)
0 approximately the same level as the medial condyle
1 distinctly further than medial condyle and distal surface of medial condyle is flattened
533. Anteroposterior length of proximal end of tibia in proximal view (Zanno et al., 2009, #327)
0 exceeds mediolateral width
1 less than mediolateral width
534. Cnemial crest proximal projection (Choiniere et al., 2010)
0 approximately at the same level as posterior condyles
1 projects strongly proximal to posterior condyles
535. Anteroposterior length of cnemial crest (Benson, 2009 #199 with modified wording; also Carrano et al., 2002; originally in Forster, 1999 #89)
0 prominent but not expanded
1 anteroposteriorly expanded
536. Accessory ridge on lateral surface cnemial crest (Choiniere et al., 2010)
0 absent
1 present
537. Medial cnemial crest and lateral cnemial crest(also called the cranial cnemial crest in birds) (Norell et al., 2001; Kirkland et al., 2005 #192: this crest is a medial projection off of a greatly enlarged cnemial crest, homologous (perhaps) to the condition found in birds.)
0 absent, only one cnemial crest
1 present, two cnemial crests
538. Fibular condyle on proximal end of tibia (Rauhut, 2003, #204)
0 confluent with cnemial crest anteriorly in proximal view
1 strongly offset from cnemial crest
539. Medial proximal condyle on tibia (Benson, 2009 #201)
0 round in proximal view
1 arcuate and posteriorly angular in proximal view
540. Posterior cleft between medial part of the proximal end of the tibia and fibular condyle (Rauhut, 2003, #205)
0 absent
1 present
541. Fibular crest (ridge on lateral side of tibia for connection with fibula) (Rauhut, 2003, #206; originally in Gauthier 1986)
0 absent
1 present
542. Form of fibular crest (Reductive coding of Rauhut, 2003, #206 by JNC 02-19-2009)
0 extending from proximal articular surface distally
1 clearly separated from proximal articular surface
543. Shape of fibular crest (Longrich and Currie 2008 #64)
0 quadrangular
1 low and rounded
544. Fibular crest distal extension (Zanno et al., 2009, #328)

0 proximally positioned
1 extends to midshaft of tibia

545. Fibular crest length (Zanno et al., 2009 #341)
0 short, less than one fifth tibial length
1 long, between one quarter and one third tibial length

546. Bracing for ascending process of astragalus on anterior side of distal tibia (Rauhut, 2003, #207; state 2 from Choiniere et al., 2014; additional taxa coded from Benson, 2009 #206)
0 distinct 'step' running obliquely from mediodistal to lateroproximal
1 anterior side of tibia flat
2 Step-like ridge running proximodistally rather than obliquely

547. Fibula (Norell et al., 2001; Kirkland et al., 2005 #188)
0 reaches proximal tarsals
1 short, tapering distally, and not in contact with proximal tarsals

548. Lateral surface of proximal fibula (Benson, 2009 #207)
0 shallow longitudinal trough situated posteriorly
1 trough absent or weak groove present, surface convex

549. Proximal fibular margin (Zanno et al., 2009, #329)
0 subhorizontal
1 cranial portion extends proximally beyond level of posterior portion

550. Fibular proximal dimensions in proximal view (Zanno et al., 2009, #331)
0 anterior portion subequal to posterior portion in mediolateral width
1 anterior portion mediolaterally wider than posterior portion

551. Insertion of m. iliofibularis on fibular shaft (Rauhut, 2003, #211; Originally in Mader and Bradley 1989; also Holtz 1994)
0 not especially marked
1 present as a well-developed anterolateral tubercle

552. Position of insertion of m. iliofibularis on fibular shaft (Zanno et al., 2009, #330)
0 proximal
1 midshaft

553. Ridge on medial side of proximal end of fibula, that runs anterodistally from the posterproximal end (Rauhut, 2003, #209; originally in Rowe and Gauthier, 1990)
0 absent
1 present

554. Medial surface of proximal end of fibula (Norell et al., 2001; Kirkland et al., 2005 #189. Also see Rauhut 2003, #210; originally from Sereno et al 1996with states reversed)
0 concave along long axis
1 flat

555. Deep oval fossa on medial surface of fibula near proximal end (Norell et al., 2001; Kirkland et al., 2005 #190. Also see Rauhut 2003, #210)
0 absent
1 present

556. Astragalus and Calcaneum (Norell et al., 2001; Kirkland et al., 2005 #191)
0 condyles indistinct or poorly separated
1 distinct condyles separated by prominent vertical tendinal groove on anterior surface (Make sure to double-check this character, because it was previously poorly defined and Turner 2007 did a much better job defining it)

557. Astragalus and calcaneum (Norell et al., 2001; Kirkland et al., 2005 #195; Sereno et al 1996)
0 separate from tibia
1 fused to each other and to the tibia in late ontogeny

558. Fibular facet on astragalus (Rauhut 2003, #213)
0 large and facing partially proximally
1 reduced and facing laterally or absent

559. Height of ascending process of the astragalus (Rauhut, 2003 #215)
0 lower than astragalar body
1 higher than astragalar body
2 more than twice the height of astragalar body

560. Shape of ascending process of the astragalus (Norell et al., 2001; Kirkland et al., 2005 #193 revised by JNC; additional taxa coded from Benson, 2009 #211)
0 broad, covering most of anterior surface of distal end of tibia
1 narrow, covering only lateral half of anterior surface of tibia

561. Notch on medial edge of ascending process of the astragalus (Mentioned in Chiappe et al., 2002)
0 absent
1 present
562. Fossa on anterior surface of mesial base of ascending process of astragalus, sometimes bearing accessory fenestrations (Modified from Novas, 1997)
0 absent
1 present
563. Ascending process of astragalus and astragalar body (Rauhut, 2003, #216; also Norell et al., 2001; Kirkland et al., 2005 #194; originally from Welles and Long 1974)
0 confluent or only slightly offset from astragalar body
1 offset from astragalar body by a pronounced groove
564. Astragalar condyles (Rauhut, 2003, #217; originally in Sereno et al 1996)
0 almost entirely below tibia and face distally
1 significantly expanded proximally on anterior side of tibia and face anterodistally
565. Horizontal groove across astragalar condyles anteriorly (Rauhut, 2003, #218; originally in Welles and Long, 1974)
0 absent
1 present
566. Calcaneum (Rauhut 2003, #219)
0 without facet for tibia
1 well-developed facet for tibia present
567. Distal tarsals (Norell et al., 2001; Kirkland et al., 2005 #196)
0 separate, not fused to metatarsals
1 form metatarsal cap with intercondylar prominence that fuses to metatarsal early in postnatal ontogeny
568. Metatarsals coossification (Norell et al., 2001; Kirkland et al., 2005 #197)
0 not co-ossified
1 coossified
569. Shafts of metatarsals II-IV (From Makovicky et al., 2009 #287; originally in Zanno, 2008)
0 not closely appressed beyond proximal half of metatarsus
1 closely appressed throughout most of metatarsus, adjacent surfaces flattened for contact
570. Maximum length of metatarsals
0 greater than 50% tibia length
1 less than 50% tibia length
571. Metatarsal I
0 present
1 absent
572. Metatarsal I (Norell et al., 2001; Kirkland et al., 2005 #203)
0 attenuates proximally ,without proximal articulating surface
1 proximal end of Mt I similar to that of Mt II-IV
573. Metatarsal I (Rauhut 2003, #222 and Norell et al., 2001; Kirkland et al., 2005 #202)
0 contacts the ankle joint
1 does not contact the ankle joint
574. Position of distally-placed Mt I
0 reduced, elongated and splint-like, articulates in the middle of the medial surface of Mt II
1 broadly triangular and attached to the distal quarter of Mt II
575. Metatarsal II proximal end of flexor surface (Corwin Sullivan, personal communication)
0 flat or small tab present
1 large quadrangular flange present
576. Distal end of metatarsal II (Norell et al., 2001; Kirkland et al., 2005 #198)
0 smooth, not ginglymoid
1 with developed ginglymus
577. Tuber along extensor surface of MtII (Turner et al 2007; from Chiappe 2002)
0 absent
1 present (Turner et al 2004 #235, originally Chiappe 2002)
578. Posteromedial margin MtII diaphysis (Choiniere et al., 2012)
0 well-developed flange absent or area rugose
1 with flange projecting caudally or medially

579. Distal end of metatarsal III (Norell et al., 2001; Kirkland et al., 2005 #199)
0 smooth, not ginglymoid
1 with developed ginglymus

580. Metatarsal III (Rauhut 2003, #220, with state 2 added from Norell et al., 2001; Kirkland et al., 2005 #200.)
0 subequal in width to Mt II and IV proximally (see Norell et al., 2001; Kirkland et al., 2005 #200 state 0)
1 pinched between II and IV and not visible in anterior view proximally
2 does not reach the proximal end of the metatarsus
3 mediolaterally much wider than either II or IV

581. Metatarsal III shape of proximal end (Benson, 2009 #212; also Sereno et al., 1994, 1996 #20; originally mentioned in Paul, 1984)
0 rectangular, medial and lateral surfaces pinched
1 hourglass-shaped, medial and or lateral surface(s) concave

582. Medial side of anterior surface of distal end of MtIII (From Makovicky et al., 2009 #289; originally in Kobayashi and Barsbold, 2005)
0 unexpanded
1 expanded

583. Metatarsal III shape of shaft in cross section (Benson, 2009 #213; originally in Gauthier, 1986 #51)
0 rectangular
1 wedge-shaped, plantar surface pinched

584. Shaft of MT IV (Norell et al., 2001; Kirkland et al., 2005 #204)
0 round or thicker dorsoventrally than wide in cross section
1 shaft of Mt IV mediolaterally widened and flat in cross section

585. Length of MtIV (Taken from Longrich and Currie, 2008)
0 subequal to Mt II
1 markedly longer than Mt II

586. Posterolateral margin of MtIV diaphysis (Turner et al 2007 #229; originally in Novas and Pol, 2005)
0 well-developed flange absent or area rugose (in *Allosaurus* and tyrannosaurids, a flattened, ridge-like and long rugosity is present in this area)
1 with flange projecting caudally or laterally

587. Metatarsal V (Rauhut, 2003, #223)
0 with rounded distal articular facet
1 strongly reduced and lacking distal articular facet
2 short, without articular surface, transversely flattened and bowed anteriorly distally

588. Pedal digit IV (Rauhut 2003, #221; see also Kirkland et al.,2005 #205.)
0 significantly shorter than III and subequal in length to II, foot is symmetrical
1 significantly longer than II and only slightly shorter than III, foot is asymmetrical

589. Extensor ligament pits on dorsal surface of phalanges of pedal digit IV (Turner et al., 2009)
0 shallow, extensor ridges not sharp
1 deep and extensive proximally, corresponding extensor ridges sharply defined in dorsal view

590. Pedal phalanges of digit IV (Turner et al., 2009)
0 anteroposteriorly short, with proximal and distal articular surfaces very close together, particularly in distal elements
1 anteroposteriorly long, proximal and distal articular surfaces well-separated

591. Shape of ventral surface of pedal unguals (Reworded from Makovicky et al 2009 with reference to Longrich, 2008)
0 ventrally concave in lateral view
1 straight in lateral view

592. Ungual and penultimate phalanx of pedal digit II (Norell et al., 2001; Kirkland et al., 2005 #201; also Rauhut, 2003, #224)
0 similar to those of III
1 highly modified for extreme hyperextension, ungual more strongly curved and about 50% larger than that of III

593. Ventral surface of pedal unguals (Longrich, 2008)
0 without a flexor fossa, ventral surface of proximal end convex
1 with a pronounced flexor fossa on ventral surface of proximal end

594. Form of flexor fossa on pedal unguals (Longrich, 2008)
0 without development of flexor tubercle
1 small flexor tubercle present within flexor fossa

Data matrix.
'Herrerasaurus_ischigualastensis'??000000?00??100----???0010-
00000000000????0?00000001?2010000000000-0--01100--00100000??-?00?0-
00?00??00?1????01???11??000?0?0000?0?????0???000????01?1?0?0?0-
000????0????????????0???1?000-
0?0011???????010??000??????????1010?000?010000000???0?000??00-
0000100?0????0010?001000?000000000000000000000100?0?01?1??0?000?00???200000000?000?00
00?????????00???????????0??0-???00?1000010?001110?10000100??0100?0?00001?0??01000-
0?0120000000110000?11100000???0000?0-0??0????00-
000001001????00??01010???00000?0101????00-1?1?0-??010000?2000000000000000?0000----
0?0??100????0010000000?000?0-0?00?0000?000?01000-
'Dilophosaurus_wetherilli' ??001020?00??0?0----1?????0-010?0010011(0 1)0(0
1)1??0?0?1001?2010001000010?0--1111???0010?0?0???00010-
01?0????111????000?011????????0000?0???0?1???000???1?1?0?0?0?10000??101?????0???????1?1?
1?0-???0???0-10?001010?01??????????1010?000?1000000000??1?10???00-
0110110?1101?0011?0011?0??0?000110000200???10?10000????1100?030?00???0?0000020?000?010
00?0???10?0????????????0??2000000100???00?0??100?10000100?0100?0??010?01?00?000-
1?00201000000110000?1?11000????11000-???0????1?00?0001101????100?00-
00???100?00?100????10-????0-??1100000100000000?0000??00110?000?0??100????001000000??0?
0?10??0??0000?0?2001000-
'Coelophysis_bauri' ??000011??0??000---????0?00-
0101001?1?10???0?00001001????0000000000-0--11100--00100??0??-000?0-
01?00??0??0?????0???????0?????0000?0???001?????0?????1???0?0?1000?????????????????0?001?0
00-0?0011???10?100-??000??????????1010?010?020000000?????000??00-
1??0100?11000?02??0010?0?100??00(0
1)00002001??000????0????00?0?03??00???100000020?000100?01?0??????00??????????0???????001
100?000?0?01?0?10000100??0100????000??1?000000-1?0000?000?0?1000??111100?0???001000-
0000????100000001101???000?000-00???0001101100????10-
1?0?0-??1100?0??0?0000000?00101?0?110???0??0?001????001000000??0???10???0?0?0????100100
0-
'Tawa_hallae' ??0001201000??00----
01001?11000?00000?1????0?000?0011?200?001000010-0--12100--?1100??0???000?0-
00?0?0-??11010000??0?????????????0?020?00?1???000???????000??1??0???????1??????????????10??0
00-00000-00-100?00-??0????0????????00??00002000?0000?0000?000100-
0???????1100?00??00?1?????0???0?????0??????????1????1?????0????????0???????0??00????0?0?00?
??????????????????????????1?10000100?0010011000???????????????0??1????000???10??2?0000?01
1100001110?000000000000-0???0-
-?0?00??00000100?000?0?110?????????10????????????????????????001??2000000??100????????????????
?????????0001000000000?010-?00?00???00?1???????
'Carnotaurus_sastrei' ??10000000001000---
0??00?010000100010001???1?1001110??200000000?000-10101000--?10-
1100000001?1101100101000010000??00-???0????1000??2??0???????0?00????00?1001??0?0????????
????????1?101100??00?01000-100?0100-0??0?1110100010100??01000000?0?0000000???0-
00001010101101?10?00??11102?1011100?000100?0010?1000010011?110040??11?0???????00????0????
?0000???0?????0???00--0000-000?01000011010?0221?0110?2000000-000111000---
-????????1?0???01?????????????????????11000-
0000??????00?00??00??0?000000100????1000000100?000?0-
00000-??000?10000?0111011??011?00?110?0??
'Ceratosaurus_nasicornis' ??10100010001100---
00?00?01000000001000010001111010101101-1000000001110?11100--001001000000010-
01110101001010001 0?00-
0?00?0??1000?02??0?1???000???0???1?1???10?00???0??????????????1?????000-???0???0-10?00100-
0?0??????100?100??000?11000?0000??0?0?0??00-
0000111?11011000000011??0?0011101?000100?00???10000000110110040?01?0?1?000?01000?0?01
000010??10000??????????00020000?0100?0?1000?????????????????10001100101????0100??100000

10001011100??1??????2????1100??0000????1?000000100100000000000-000101000100100000010-
00000-
0?1100000000001101100011101111 0?0?0000?1000???001000011?????????????00?0???????0?0?
'Limusaurus_inextricabilis' ??10001010011101 01?00?000?11?0?1?01?000?---
00000?000??01-?000000000-10012100--00120??0??00000-
00?1????0000?0?020?00-?????01?000??0?20-?00?
?01?1110-?0-1000-??0?10?0??0????11------1----------------------
000??0?1101?1?2?0000-??01?0???0??????????????????00???1????0??????????10000?000???0200000
00-0??1101000??0-10002000000110000100001?10011011?000?00000?000000----?0000--
010001000101(1 2 3)100-00011--02???000000-
000110001000?1001001001000000100???0?0000?00-??000?0?00?00?1???00?010?0?0????00011???
?????????0??10??????1???????001?0???00??0??00???2?00000-
'Majungasaurus_crenatissimus' ??10100010001000-
-??1000001000010001000111 00?1011010112010000000010-101?1010--010-
110000011?1101111101100010 0010?00-
0000001?1000012??001?00000001??1?????0???0?0?????0-?????????0?10110???0?00?????10?0?100-
0100001001000101000000110000000000000000100-0000111010-
1?10000?011?0200011101000010010010 1?0000001010110030???1?0011000020000001100000000?10
0100??????????000-
00000???000?0?00022100100120000??????????0??????????????1?0???01???11????????????????0100
1000001000100 0?0001001000000100????????????0???0??????-??0???????????000000??0010001110
1101010000111100010000100010??0??0???0000000?0??0?0000011
'Cryolophosaurus_ellioti' ??????????????????????????????1??1?1??000??0??0????0?2?
????0?00001011??1100--001000????00000-
01?00??10?10??00????10?0?0???????0???????????????0???10101?????000??????????0-????0???????
????????????????0-0-110??0????????????????0(1 2)??0?0000????????????????????10-1(0
1)????00?????0?????0?????0?00?00???10010??0?1?10?0????????00???0????????00?0?0????0?00????
??????????????????????????????????0?????????10000??
??????00??????????????0?0??1??10?????000???????00????????00????10??????01001?010??011?0??0
000??????????0?????????? 001100000?????????????????????????????
'Monolophosaurus_jiangi' ??001000100101?10000?00?01110001000?0000??00111100
0100201100000001111??11100--0010000000-000110011000-
0011000001??010????????0000?????0?1???00??????1010100?100000-??????????????0?0011110-?00
00-00-100?0100-1????0100211?1010000002000?00000000000?010100??1101?10-
1?100?01011?00010?1001010010000000 11?000000?10010030?00?0?????0?02????0?????0?????????
???
??????010010000010 0100?0?0100001000000000 0-??0101000100100?00110-
100?0-??
'Piatnitzkysaurus_floresi' ?????0?????????1??????????1000????110?00100?1???????????
??????????????????????????0????????????????0????????????????????????????0??????0?1???0?????10
??1?1?0?10?0010?010-?????????00????0?10?????????????0???????????????????????????0?1?01?00?0?
?0?0????0100111110?10-
1010?1?0011????10??001?10010???????11000????1?00??30??0???0???0?02????0??????????????????
????????0??21000?0?0????00?0??1?0?10?00???0??100????0??????????????????????????????????????
???????0100??0????????????010000???????????1?00????0000?010?????11-
1???0-??110010?0100010?0100??0??10111???0?0????0???????????????????????????01?0???????????
'Baryonyx_walkeri' ??0101201001110??????10?0?11?0?000??00100000101???
?0??2??????0??0?101??10100--
0010011???10?000??1????11???????020?010??????????0000000??1?10010???0?10101000110000-
0?10-00-10??0?????11110-?0100-00-10010??1111000???0101?103000000?200000101021010 00100-
1101111?10-
1010?001011?00010??011210011 00??000110000??0100100?????????0??0002????????????00???01??0
?101010???0000-???00?00?0?10000?2010?0201000101210011010 1???????????????????????????0000
????0100?????1?0???????????1000???0000100????00?0-????0????0?0100000?100?0000-
0?010110??????1000100???????????????0?0100000?00???????????????????????????????????????
'Eustreptospondylus_oxiensis' ??0??020100?1100----
00000?1100??0001001000?0???????1??????????000?1-11011000--0011011?00000010-
010?0????010?00000?00-????????0??0?????0?1?00010??01010001?0111?100-??10-00-
10000??0??1?110-?0????0101001?????????????0?????010000002000?0000000101000100-
0110111010-11100101011?00?10??00100002000??000110000??01000?030?00?0?0?(0

1)00002???00?00?????????????????????????????????1?00?0?100?0?20001000l?0000??????????????
??????????????????????????????????????10???0??0???01000?000000110000000010000001010000l
00010?0?01?000-
010100100000001000100010000011100000001100000000100011??00?????00000100000?001000-
'Afrovenator_abakensis' ?????0???????1?101001??0??11010100110?00000?1???????
0?201100??0?001010011100--00?????????00010-
01??0????01000000l?010????????0?0??
????????????????????????????????????02000100000???????0100-0110110?10-
1?0001?1?11???010??0?(0
1)?10?10????????1?00????????0?????????????0???0??0?0?0?100???????11?00???????????????????????
??????????0??0?01???????00?????????1???011??1??00???????121000?2???010000000100100??0????
00?00010000100?0000000-
0?0?01???0001000100110100000-?0?0?1100010001000100????0?0??????001????00?0000100?111??1
?0?110????01?????2???0???
'Allosaurus_fragilis' ??0010(0
1)01001010?0000?0000?1000010?110?0100100001?1?011?1-000000000101???(0 1)100-
-?111010000-01000-010?0???01?0100010101120?00010??0001001001?00?000010?00101001100100-
1?10-?0-0-?000000010000-?0000-00-10000110-
11?00010010101020000002100100000000000000101000001110l0-
1000000101100001001011010000100000010000001010010030?00000000000002000000100100 0???1
?0100??????1000002100001010000000000010000000010000000000000000101000011101000001000 10
1210000211100000000010010100010001000001000010000000000101000?101000010000001101000 0
-000101100011001100100010001011l0000?0001000000001100011 10?1?0011000000l010?02001000-
'Neovenator_salerii' ??00?010100001010000l0000?11000100110001010001011l
0??0???????????????????????1???????????????????
?????????????????????????????????????1?100-?000??00-??00??????????????????102100000?10010
0000000010?010100001111011111100001011?00010??00200000010??001101101??1100100?0?10???
????00010000010001 0?10??100100?????????0002100101?10?0?1010?????????????????????????????
??0110100??010001000??000001000??1100101001010100 0
0100000011000000-
000101200011001100101010101111100100011010 01????100?????01??????000?01?10002001000-
'Sinraptor_dongi' ??001000000000?10000000?010-
000110110000?0000001000000200000000100101??11210--?110011000-010010011000-
00010000010?0113000001000010000 10?1?00000??10?00101000100100-??10-?0-
0-?0?00?1011000-?0000-
010100?01110010000??0101010100000002100?00000000000?010100110111010-
1010000101l?001100101101001010001001100000101?010030?0000?????0002?00?0??1?1001???1??
1??1?0000???0????????1010?00100?0???????0???????????????????????????????0????11001012100??
???1000000100100101001???000?0??1000010000000001100??11010000100000011010000-
00010?100011001100100010101011l?000000?00?00000010000011000?0011000000l010?0100?000-
'Acrocanthosaurus_atokensis' ???010100?0??101?0??10?0?10-?0?1?0110001001000?0?0?0
112011??0?00001010??1110-
-?11?0??0???0110111l?00??10110??01001?1120?0??1?0??0?????0?1?0?0?????1??0101?0?10000????
10-?????????0?????200-?0000-?10???0?101011???0??0?0??101??000021001?00?0?0??1?0???1000?1
??1?111110??001?11??0?10????2?1???01??????10?1?0??0??01?0?1??????????0?02??????10?1?0????
1?010??????????0???1000?10100?010?0?0100000??10????02??00?101??0?0??011101?000?10001002
200002??100?0???00???10???????????????10???????????10?1010??0?0(0
1)0??0????00?11?10?00???01012?1001?011?0??00100?101?????0?0??????0??0??100????001?000-
00??0?1010??2?01000-
'Giganotosaurus_carolinii' ???0?00????????0????10????10???1?001000100??01?1?0????
2???????????1010??1110--??1?0??????01101111?01??1???????1?0??10(1
2)??????????1?????????????0????0???101?????????????1??????0-????1?????201-?0?0???10???0???10?1
1?????????????1???000200?0?10?0?0??0?0???110001??1?111110??(0
1)?1?11??0?10??0?2?0???0???????10010????1?01??????0???0?000?01??????000???0???1000???????
????0????????????10??010?0???
?0???10000?110?1??0?0?000?11?????10?1010??010100?0???????11?1???????01012?100??01??0????
?0??10111???0?0??1?00????1??????????????????????????????????????
'Aorun_zhaoi' ??00??00?10?110101001100??0-
00010011100????00000?0000?2000100000000-0--1110110?1100??0???100?0-
00??????010??0?0000?11????????0?0???0?10??010

-00000-00-0-0?01?0-1??000??111?01010001002001-0010001100000100-
0???????1101?101001?11010110000?00?00?00000000??0?000??00???????????????0010???????????
??1??0??00???1?10000110-
10110111?0?00221001212101000110??????????????????????????????????11????????????????????
???????????????????????00000???1000020?0??????000?100110?00100011?00000?01000?00100
0-
'Ornitholestes_hermanni' ??00?0000?01?00101011100??10000?01101000000000?0?00
1110001100000010-10011100--00101?0100?10000-000201000?001000000011(2
3)0????100000???0?0?????0?01001111101?100-????-????????????????0010?10-?0000-00-100?00-0-
11100011010001012011102101-001000000?010100-???????10-
1?101200011??111100?11?1001??0001001??100001010110?0???10?0?10?0020000?10?0??????????1
?1???????????????????????????0?10?11000010001?0??0?0?0?0?????0??????????????0???????????
???????????1?0101??0100000?0?0010011000000?10????010?01000010?000001?10000-
0????????????10?0?10?????????????????00??0??????????????001??????0?100?0?0?0?0010?0-
'Zuolong_salleei' ??00??00?00?010101001?0?0?0-
0000001110000000?????00???????0??010?0-0--1?10111011???00??00000-0?1100-
00?100000001011300??0????0???
????????????????????????10100001020?0000100???????010100??????10-
0?101100011??0?100??00?001?????????????????????????40?001?000??000000??0?00???????????????
??????????????????1?10???1???0?10001???0100???????00?000?????????????????????????????????????
?0???00100??????????00?0???0???100?00000011?0010112?00????????????????????1?0110001000010
01000001010111?0???011??000?????????????010????100010101000????0???
'Albertosaurus_sarcophagus' ??1????0000011??0????10?1???0??0??0100000101110?11??
00201100110001111011201??000-
12000010111100112010101?00101010010300000100000000000?1?00010?01??1000?00110000100?1
0-?00???0000?001??00-?0?00-00-
10000101011?011100???010??00?10?00??0?0000000?0001010???011?0???????0000011?00??????0?
0?00?????????1??000??0?00?0041?0?00???10??0??00??0??0?00?0????0??0000001??000020000???0
0?0??0000?11?001????????00??0?00??01000000010-?0????????????3200?0??-?0000????????10101?1
00000000?20???1000000?0010??0001???000100000000102000101?1101100110000100100000001?11
10011001?1000100?2000110?001?0011000001111000?0?1000-
'Daspletosaurus_torosus' ??100020100111110100??00?10-
00000101?000???11101??????2??0001100011111011101100011?01001?000?0-
011201010110?1?101001030???01?0000?????????????0?00?????????????????100?10-?0-
10?0000???1?000-0000??00-
1000010101????????211?101000010200000000000000000010100???????????????0???0????0?????????
??????????????????????????0?????????????????????????????????????1?111??????????????2????????????????
???????01???0????-????????0?????????????????????20?
??01?0002???1?0?10?????01?111???2???????????????
????????1?????????????1????????0?0-
'Dilong_paradoxus' 0-100020000110?101001100?10-
00000011100????010210100012011001100000-11011100--0010001100000000-001200-
0001?00010??011??0?????0000010010?1?0000000?????1?1??1??0001??0?????????????0?0-11000-
00000-00-0-0000-1011111111010101010000102000000000000100000100-0??????10-
1?100000010??0010???0?01001?????????????????????1????0???????????????0?0??????2?000?0??1??????
????????0?????????1?10?0000?01?1???10001??????????????????????????1??1??10?110010022?0??21??
??00000??1?0??1??010000??0??20000100?0?0???11??????????????0??????0??0????11?01?0001?00?
?0?10??0?001?111??010???10???0002000111??01???????0?00?0??0?2???000-
'Gorgosaurus_libratus' ??101020100011?101001100110-
000000010000???1110?11101?001100110001111?11110????0??1210000001?0-
00?20101011??10?0?????3?00001000000000??0?1?0000000?????????????000?????????????????0?00??
000-00000?00-
1000010101110?0??0????10100001020000000000000000000?????????0???????0?0??????00??110??0
??0000000?0?????0000?1????0?????????????????????????????000???????????????10???????????1??????
0??02???32????????????????????100101011100????0
?12100?100?000?00101?0001???00?10010?0010?????????????????????????????00????????????0?0?10
???????????????01000110??????1????????0?0-
'Guanlong_wucaii' ??10100000011111010011001111000000111000010010110
001012011000100000-11011100--0010001000-00000-
0012010001101101200011300?10??000000000011000000001101000100011000100010-

1001000000?001?000-00000-010100?00-10111??1??0????101000010200(0 1)1001000?000000100-1??????010-
10101000010?1001000010010010000000010010001110010030?0010?0010000000?0??2000000????
?11??????????00020000?1?10000000?01100110000100011100010000010100000010-
1001001100100210000212100 0???0010010101010000000000210011001000010101100010000001000
000000-
10001001 1?011000110011001000001011111 00?2000?1?001000110011110 01?0011000000101000210
1000-
'Kileskus_aristotocus' ??10??00?00?0?11000011000?0-
00??011110000100??????01???
???01?10?01???
???????0110001021000000000???????010??0???
???
????????????????????????000???
??00??????00
1?1???????????
'Proceratosaurus_bradleyi' ??10??0000000111010011001110 00?001110000???0??1???01
0?0011000100 00?????01?0????01????????????00-?0??????0110?0000???112?0??0??0?00011010?1??0
11?????????????????0??????????????????0?001?000-00001000-100000-
10111100??0???01010000102101-0010000000010100-
0???
???
???
??
'Juratyrant_langhami' ??
???
???
????????????????10-
1?100?00011?00???0?00100001000000??1?0???0010?00??030?001000???000????00???????0???1????
???
??????0100110??1100?0100??21001100?00000011?1000?0000101000100000 1?0?101????????0????1
100101010101011000 1??0???
'Tanycolagreus_topwilsoni' ??1??000?00?00?????????????????????????????1?0000???0??
?????0??00000-
101122011000100??0???00?11001??????????????0000010???
???1000????101??01??0?10??0??000?0????????????
011???1??0-??1?????????????????0?0?000100??????100110??0?001?0?0???????????00?0???????20?00
????1???????????????000200000111000000000110111000110001110000000101?11000010-
100000110000022100020?100000000???????????????????????????????????????1110101???00?????????
?????????1010110001100010010000010101110001000110001000200011110010001000000010?0002
001000-
'Tarbosaurus_bataar' ??0???000000011010000?100110-
000000??0000????11001010012011001100 0?111101???100001??01000-
0?1010?112?111011001010??01031000010000?000??0?1?10010001?000001001??0000-??????0-????
?0??0011?00-?0000-00-
100001?10111011100101010100001 0200??0?0000000?00010???????????????????????????????????????
?0????????????????????????0??0????0????
??????0?????????????????????????????????0-??1?0???02???32?0???????????????????????????????????????
???
?????01??????1??????1??????000?0-
'Tyrannosaurus_rex' ??10?00000001101010111 00110-
000000010000010111011010002011001100011111011101 10?111101000-
011?100112011101100101010 0103?00001000000100 1001?10?00001000000 10010-0?010?010-?0-
1000000000011000-00000-00-
10000101011?011100101010100001020000000000000000001010000011 1010-
11?00000011?0001011001000000000000001000000000100100 31?00000?01000001000000020000000-
100110??????1010002000001010000000000011000100 12??00010001001001?0?000010-101000------
032000021--
00000?000100101011100000000?120001100000010010110001010000100000000102100101011012001
21001000100010001011 1?0010?0??0?010002000110 1001?0011100001-110002000000-

'Stokesosaurus_clevelandi' ???
???
???
???
???
??????????????????010010101110010100??2100110010001 0????????????????????????????????????
??

'Xiongguanlong_baimoensis' ??10?000??0?1?1?0????100110-?0??011?000001?0102001?
?0?0011001100010-1?1??100-
-?0111??0?00001???0?120111011??1000000103?001?1?0000000???1100000???0??10001000110000-
???101??0110210000000???????0100-
0000111010-
1?10000001000001001000000010?100100100110?10100100???????????????????????????????????
???
??????0100??1??1100?000001210??10000000??????????????????????????????????11001000110011 0
0100???

'Compsognathus_longipes' ??00?011000????1010?1?00??1101000???0000???0000000
00??00001000000?0-0--111?0????1?1101???000?0-001000-
0?0??????0????????????????0????????1?????????????????010??0????????????????????0011000-?0?00-
00-0-000100--0?000??1210?1010001002101100100000000000100-0??0??1010-
1?0?0?00010??0?1???0?10?0?200?00???????10??0010?103?????0??0000?0?000?01000010000-
1?1000??????1??00020000?1110???000000?0??1???1?00??01???000000????0?01011???00?1000?1?2
2???12??10000011001??0-
1000???0???0??0?0???????00?001111???0010000100010001010 0?0-?1??????000??1????1???00?0???
???00?0??????0?0001000?10?001?0011000?00?0?0?02001000-

'Huaxiagnathus_orientalis' ??0???000001?0?10100??00??11000???11??0????0000?1000?
????110?0000?0-0-
-?1?0????1???1?????????0-?0??0????0??????????????????????0??????????????????????????????????
???????????????0?001?000-00?00-00-0-0????????????????????10??0??00?0?1-00100000000?0100-
0??????0?????????????11??10????0??0?0?100??????????10???01??10?????????????0????00?0?120?100
0??1?0000??????100000200 00?1110?00000000100???????????00????00?00?0100?001???00?0?10?0?
1?22???12111?000000?01?00-
10001010???0??0?0??02??000?1011?????????0001000000001010000-?1??????0??????????????0??????
??????0???0????00?2000110?001?0011?0??00?0???020??000-

'Sinosauropteryx_prima' 00000000001?001??0?0?????0-
0000????0?0????0000????????????100????00-0--?1100????11?????????0??0-
00??????00??00012?0?10???????????0??????????????????????0???????0??????????????????0?001?00
0-00?0??0??0-0?0?????????????????10100?1002101-0?1000000?000100-0??????010-
1?1?0?10?10??11????0?20???100?00???????1000?01???03?????????000?0?000??1120?10000-
1?1110?????????00020000?111000010000 1200?00?00?????01?0??00000?1100?0111110?1??11?010?2
2???12111010001000 1000-
1000???0???0??0?001020?000?1011?????1???0001000000011?100?0-?1??01?0001??0??0?1????0?01?
?10?00?0???0??0?00?20001101001?001100??00?0??0020??000-

'Nqwebasaurus_thwazi' ????????2??????1000??1011?0-00??00?1???----
0??????????????????????0-10?1?20111011002010 01??0????010?0-
0???????0?00?10?????????????????????????????010?00?101???0000-??????????????????????????????
??????????????????????????????????03?11-21211???????--00-0???????10-
1??01200?0-?11111???100100???0???????0??????
?????1002100001?10000000??0100?100?0100?10??0?00000010?000000101001001000101221001 11
1001010110????????????????????????????????????1???0?0????00??????????????????1??0??1?????000
0010000001011111?0010?0010?0?0002001110?001?0011000000001000?0101010

'Anserimimus_planinychus' ???
??0???????????????????
???????????0??1?????????????-???????
??0????????????????????????????????
??????????????0?????????111200000????0100000?012????????2???????????????10000?1010-
01?1001001?002210??11111001221 0????10100?100?11?0???????1010000?0010???00???00?100010
0010??000-??001?1---?00?010110???0??1???

'Archaeornithomimus_asiaticus' ???
???

??0???
??????????????00----
20220011000121100000000020000000010000000000010031?1010?0?100002000000?00??0?????0?
???????????101210000111001?1000?00200100012000010000000000???001010-
11?100110110022??011???00011210010?0-00001000100001000001000010??00101100011-
010010101000100000100101001000110110001000001011111000010?0????100002000110100101---
100010011000?0??1?1?
'Beishanlong_grandis' ??
??
??
????????????????????????????????????1??
???????????1???????????1112100001?00010000?0002????0001000000000???000??????????????????
?????????????????000002?0???1?????????????10
?0??00021?0110?10001010???1100010?0?1010100?1000110???1?001?????00??10????001011
'Gallimimus_bullatus' ??0???0021000000101?101110-
0?000???000-???00000000?11001?110?000?0-0--1???11101120001000?0?0-?000?0-
001?010112??00-??01?010?10?0?110??1?0000100010?0001001??00011??110?0-100010??10-
0??10?1010-00-0-0?01?10?1?1000-0???011------1---??-?-?-2---???--
-?????10??010-??202210010??12?????0?0?????????00?1??000??0??0?0040?1??0???100?0??00??0??0
?01?0????0??0?????????011200000??10?1??0000?02?0?0???2??0000??0000??1?0??001010-?10?????
???0?2????0??2?00011010?1?010100?100011?0??0????1010000?0010??01001-?00010001000101000
0-110?????00?1001?0????00?00??????00???00?0?0100??0001???00101---100?01?1?010?0001010
'Garudimimus_brevipes' ??0000002?000001001?0101110-00000111000----
0?0000000??01-0110000010-0--111011101120?0100-000?0-00?000-
00?00?00?001?113?01?01001000?11?0????00010001?000010?01100?11?????????0???00???-
0001001?10-00-000?00?1001?1000-0?1??11------1----------2------------000?-
1??????????????0-???2???000001001000001001???000000001?040?10100?0??0000???00?0??0??0???
??
???????????010011100010011100?10010010010001001001000101020??????????????????101011000
11001000110010001011100000?0100?010002000110?00100011100000?1101020000011
'Harpymimus_okladnikovi' ??00000021000?010?0??1011?0-000?0?11100----
000000000????????0???????0--1110111?112010?00-000?0-00?000-
00100100????????????????0??0?0010001001011
000-0000010100??0000000-0011------1------?--11?0??0?--?--
-?????1010-???0??0000-??02?0???0001?02??000?00????00??110?11040?1?10???1000020000?00000
01????0?0110??????????11??????1?10?1?100?0?12?0?0001200000100000000011?10000010-
10010011001102210002111000111100100101001???11?0??0??00100?000000????010????00???????
????????????0101?00???0??????????????????????10????????0001000110?00101---
100000?0?000?0001011
'Ornithomimus_edmontonicus' ??0?????210000???11???01????0?020??????????????00???0
11?01111000000?0-0-
-????1111112?0010000???0-?00?????010010101??011??????????0?0?11???1?00?????0???000?0?0??0
?0???1110?0-10?010??10-0??10?1010-00-00??01?10?1?111??0???011------
1?-???-?-??2????????????????10-?010-?1??22100???102?????00??00???0??1??1??0?0??0??0?0040?1?0
0???100?0??0???0??0?01?00-??0??0?????????11121000???00?10?0000?02?0?????2?????0??0000??1
?0?0001??0-?1?????????0?22???0??2?00012210???010100?10001100???????1010000?0010??010???
?00010??10001010000-
110?????00?1001?0?10?0???0??????00???0??0?0100??0001???001?????100?01?1?0?0?0?01000
'Pelecanimimus_polyodon' ??00000021000101000111011?0-
00010011100-???00000?000000001111?000??0-0--1120111011202?1?0-000?0-
00????????????????0?010?00???1??10?????????1?00?01?????????????1???01????1?????10????0??00?00
1000?1??00-0-00010100??1?0??0????1030001103201-102110201010--10-0???101010-
1?????00010?1???1?0000?1??????00??????????????????0??????????????????????????00?110??????00
0001???1?????????1?10?10000?0??200?0001?000?0100?0000011??00?1010-
01?10?110110?221000112100011210??
??0??
'Shenzhousaurus_orientalis' ??000000021000001000??1011?0-000?0011000?---
0000010001?????1????????0-0--1120111?1????0?00?0?0?????00?0-
0???0?01????0????0???1???0??????????????????????????????????0??????????????????0?001?001000010-
00-0-000??1??1?0????0?????1------1---1-----11001001--00-

0??????????????????0????????????0?00???200?000??????00???0001?041?1??0??????000?0000?0??0??
?0??0?01???01??10?22
10???1210001011001000-
10001010100???1000100?000100100101001?0101000000000010000-???1????001100??00100?????
???
'Struthiomimus_altus' ??0???0021000??1?10?110?110-0000011?000---
-?0000100?11???1110?00010-0--11?01111?12000100010?0-?000?0-
0010010112?1011??01?0???1??0????0?1000?????0??0000??0???0?010??110?0-100010?000-
0001001011000-00??01010?1?1010-0???011--?-??1----------2-----------
-??10-?010-?????2100???002?????000??????0?????1??000??0??0?0040?1?00???100?0??00??0??1?01
?0????0?10?????????111210000??10010?0000002001000120000010000000001?0?0001010-
010101110110022100001?2?00011210010010l???10001100?10????101000000000100100001-
0000100010001010000-110?01?00011001?0010?0?000???1100010?0?10?0100?200011??00101---
100?01011010?0001010
'Achillesaurus_manazzonei' ??
??
??
???0?2?0??00??1121???10??
?????????0?0??
?????????????????????010????????0-
-?01102?0????111????0??211???0??????????????????????10???
?????100(1 2)001110?00??????0??0?0?010??????????
'Albertonykus_borealis' ??
??
??
??0????????????????????
???02101011??1??????????????????????
?????1?????1?011?12???
??????????????000?111111001??????????????????????????????????02-01????????0??
'Albinykus_baatar' ??
??
??
??
??
????????????????????01?????????????1??12?0????????1?00??????????????????????????????????1??????
?????????????????????????01???10010?11?11111??11100011000002?110102010000-
'Alvarezsaurus_calvoi' ??
??
??
????????????????0---?00?0?0?0-
00121011000?1???????????????????????????30020????????12100?0?1002????????????1?????????????00
0-
00001?0010??000?0???1?011?1101000-
10020--
0110021002001101?00????????????????????????????????????00?11?1??001???????????????????????20??
??????1011??11111001??????00?00?01110??10000-
'Bannykus_wulatensis' ??
???1?01???????
????????00-
0001??????????????????????1??????????????????01011???
???????1(0
1)01??111010100?1?111????1?????????????????????????????0??0?1????001??00???1?00?0100??0100
????????????111100001?0?0010001012000002100101102110110?0?1?1100011111011101111100220 1
112???01000111010???????????0010010????11000000??????????????????????????????????110001000
1000000011100010111111002011 0??00000010?111?10010????1000000010002?01????
'Bonapartenykus_ultimus' ??
??
??
??0?0??00?0????????10011010110 1????????????????????????
?????????????????????????1110-

11101?01001?001???1??????
??110????????????????????1111??0?1??????????????????????????????12??????????????????????????
??
'Ceratonykus_oculatus' ??
???11?01-1?0????1110111?1120????0?100?0-00??????100??0?????10-??????????0?021????0?-
001?????????????00-0000-?1??????????????10?????????????0??0-
0??1???0?221??1???????????
?????????????????????????????????01?1??????????????????????1011?????1?00-
0000???????????????0????10?????????????????????????1?????0?????????0???11?2???????????????????
??????????????????????????1?????????????????????????????????1???????????0??????????????????????
?????????11211?110??010?????00102-?1001????????
'Haplocheirus_sollers' ??00??001101111101011100111000010011100?0?00001000
01?01-1100000000-0--1000111?112010000-10000-
00??00??010011000??010?0011010000021?0001?0001000010?0111??00-
00110??????1?110??00?1011110-0000100100-
0001111010001??0210010100010012011001000110100100-0??????00-?--
101?00?10?10111?101001001??0100001??1??0??1?101030?00?0?0?(0
1)?00??00100?00?00000??1?01???????????1102000001111?1000001011001101101101100?00?1100001
?100000010101101110110022011121210101000101 0??????11-?01002?000001001??000011010100?
?0100110000011011 0?0-
01??0110001000000?1010001011110?00?011010??000?20???1?10010?????00?00?010002001000-
'Kol_ghuva' ??
???
???
???
???
???
??001?0011000002-?11112010?0??
'Linhenykus_monodactylus' ??
???
???
?????????????????10-1??0??111????1210????121212????????????00???0?11?0?(0
1)?20?10??10?121?00???0020????????1???1011?0???
???1?10101111010111111-
210032111?2???11011?120??0?
?1?1???0010???11??0????0111??11?????????112??0?1?1?01?00110?0002-?1000?????0??
'Mononykus_olecranus' ??
???
??1100??????????????1001011??0??1??1-
212?1??0????--10-0???????0-?--
1010?11????1211?101021012001000000-?10100???1010???2??1?????0121?0?1??????????????????10
1100???1000-00001?1100110000023000010 1111-
0021000111011?101011110101101011110022111 12-?111011?120?0?0-???????????????1?00111??00?
310---???1???00?0-0000??-?????0-?10011112-
2001001011100001101111001 1?11???1011??1101101001?0011000102-11000?010100-
'Parvicursor_remotus' ??
???
???
??(0
1)2??12?0?00???0?????00??010????1???1?????0121???10??
??0????????0-
-?????2?01?0?0-?1????3?0---1---1--0000-0000----1(1 2)00???1?011112-
2001001011100001011111000110 00?01?101211011010010????000102-11001?001100-
'Patagonykus_puertai' ??
???
??????????0??????????????????????1??
??????????????????????????????????????0?00?001?0?01???10000?01???1???30?2011000??012100?0
02??????????????????????0???1000-
1110????????001????0??011?111100201001 1???????00?10??101?????????1??11112?????011?11010?
??????110????????00100111????02?11011---01001????????????????0-

0??????11120001000111000011111111??10111??000010(1 2)001100110??????1????000??????010?0-
'Shuvuuia_deserti' ??000000110000010101?101102100000?11000-???0000000
0011000-11001-1?100--12201110112020000-10000-001000-
0100000000??10-??01?1200000021110101--01001011111?00100-
0010-??11001011110001100?001000001000-0-0000-0-010011110??001????0??03201-212110202?10-
-10-0??10-??10-
10100211110?1121011?102?01???000???0??10000?001?1050?20?1?0010012100110100200101????1
110101100???1000-0000110?0010000000230000101?11-002????110?11?101011110-011010112--
02211112---1101121201000-10020--0111021012001111000?310---1--11-00000-0000----22000-
010011112-200100101???0??1???101?001??????101112110110100100011000102-11000?011100-
'Tugulusaurus_faciles' ???
??
??
???0??00??????0
???00?1111101?????????
???011?2???0?000?01??101000
00???0000001010001011111???20????????001010?1101???????????????????????????
'Xixianykus_zhangi' ???
??
??
???????????????????????????????????????0??2?01??0??????0-
0100???00100050?2211?10???101??
??01000-?0020--
0110??10110110111???310---1---0100000-0000----220?10010010112-
20010010111000010111110011???100101011111110?1110????0?00?2-?10?0????????
'Xiyunykus_pengi' ???
??????????????????????????????1010??????????????????????????????20???0?????00201101100111011
110?00110001000011?????1?0110?0??1???????????????010??00010110100100-?????????????????????
???02???0?????????????1?1(0
1)010001101010001?1?11000100010?0?11001001???1???1100????????????001000?000000?????????
?????????????111100000110?10010010110010020001011?21101???????????????????????????????????
??1?????????????0
000101000101111110020110??000?0??1?????1001?001100?0000010????0?0?0-
'Alxasaurus_elesitaiensis' ??????????????????????????????????????0?????????????????
??
???????????????????????????????1?00110101??10-
101?0??????????????????????????????00110?102110001010?0??????????????????0????????????0?00?00
1001??0011??000??01001004??1??0??????????01??00?2?01??????0000??????????0?????????????0???
??0?1000100111001101???00??0?100010000-
00?0??1100101220??02???00100100???110100?110101?0???????0-??000?20???????1?????011110?1?
1?220?0-???????111?00???0??0?10???????????????1?????????????????00?010-?00?00???0?0?001?0??
'Beipiaosaurus_inexpectus' ??
??
?????????0??????????????????0???1?00???1?1??10-101??100-?????????????11------
0220001100132010001010-
0??????????????????????1?????0????????0????????????????????0?????????????????????????????0?
??????????????001002000001?10?0?000?0?100??????????10????????0????00100?????0?0??????0?22
??????1?00?0000?01011110011??????1?????????????????????????0????????????????0???????1??????0
0???0?0?0??0????0?????010????????10?0??0??????001???11???????????????????0-
'Erlikosaurus_andrewsi' ??100000100110000?-0?101110-
00001101000????0?00???11????1000001010-0--?1100--?110?10000010000-
00100??0000010000?0??0???1012100001---
01?0???00?00?1000000000-??00-????????10??000?1010?011?1011010-101000-0-11?0000-
0100011-?----
0220001?0013111?001010-???
???????1??10??????????????????????????????
??
??0?????????0??0??????????0????
'Falcarius_utahensis' ??????????????????????0????????????010??001?0???????????
????????????????????????????100000?0?00-

00??0??????????1?10010??????????????1?00??00?00??1?01000?000??0?011?110-
10011010??0??1?00100000??00-1010?????????????????????????0?20001(0 1)00101110001110-
0??????1101?212100010?11211001120100200100110100101010?0010041?101000?1000120010011
000010???101111?????01011002000 1?1?10000100 1?11001?0010000111100110 0?000110010010-
1010001100110220000211100100000010111100111010101?00000011010??110100101010100011210
23001022000-
010?01101011000000100000101111100010?11??010000100011010001001100??001000???001000-
'Nothronychus_graffami' ???
???
???
?????????????????????212?????????2??????20?00?001??111????????10???04??10?0?0?2010??10?0?
?0?000100?????00???????10100?2????00???0?0100000110?1?1111001??100?100000?????????0-????
0????????22?????????00000000101112001 1??1???1?0??100???1100?10101000111--
2?0121?0?11102?10???0??112111100??00?000?0010110???111??1101?0??0?2000?1??0001010-
0000001?0110?0?0??0-
'Segnosaurus_galboensis' ???
???
??????????1????????????????????0010?????1?1??1??101??0-
0-?1?000??0???0????????0???001?0?10111?00101???????????????????????????2???????????????????
???????????????????1??0???2??????????10?????1???11?0?0?
?1100110????????????????????????????????????22????????00???????1011110011102010000101000-
01000?201010????01000011110?1?102?101??0??????????????0?0??1??0???????11???11?????0??100
00????001010-?00000??01102001????
'Nothronychus_mckinleyi' ???
???0?1
??00?0?0??1??????1??1??10?10??0???0???00110?101
?1???1010-
0???????110??2??010011?11?????112010??001???11100100??010??00????????????100????0????????
?????????????????10???????????1?0??0?110?0?1101?011110?1101000100??0????????????0??????????0?
??????????????0000????1?????????????0???????????????12???????????????12110?111021100-
00????????????0?????????0??0??????11??111?1?10?????????????????0-?0??????????????????
'Avimimus_portentosus' ??????????1?1???????????????????????????????????????
????11??1?????????????????????0000?????0-?01??????0?00?000???11?????????????1?1?0??1?10?00?
?01??120?0?0??0?00-?110-????????0??00????11??????????11??10-
0-?0?00???????111-??-??????????2??2????-?-
-?????110?11101001200011 0?112111001001001000000111100000?0000011060?1010?0?2?0000???00
??????????????????????????2002000001100100001000110010010100001001000000 01?111????0-???
?1??????0??2?????????????????0100??1???100001110101000 1111?00?00100100011-
0001111023001 0?0000-
01101121011010000010100000100110001 1?11100010112000110111100?11000102-110102010100-
'Caudipteryx_zhoui'
100000??0?111?0?0000????????0?0?0?11??????????00???01?????000?01?0??????1?0101?10-?10000
010??0-
00??0???00????000??0?????????????1??????????????????????????????????0??????????????????0?201?001
101111100-110?1??0-???0?0?????0?10???1101?--??-12??2????01-
-?0-???????0?0-????1??00???????????0?(0
1)0????00?????????????????????04??1??0???2100??11????0??00?0???1?011?0?00??11000???????1?0
0?001?0?0?????1??10?00??0000?0??0001?110?0000-?0?1?111?010?22???021-
100?000000100101001???0?1?10?0?000?????00??????????01???2011110??00102?0?0-?100?111111?0
?????10??00?????????011???10????012000 11?1001??0????01?1?0???1?0??000-
'Chirostenotes_pergracilis' ??1????????1?00??????1101??????????????-?????????????????
??1?0100???01?1???0???
120?011??0?00-1?110??????1????10-0?01111111100-100-10-0-?0??00??0?????????????1?-
-????-??2????-??????????????110??211??0?????12?????1????????0??10?1??1???????????52?10????0
??1?0??????????????0??????????????????20?200000?????0??10???????????????????????????????????
?????00?0????0???22000???????10000??1000-100010000??1??010000-
000000??12??00?????2011110230011 21000-
01????0?0??????0?0?11??0?0??????00??????????00?????1???0?1??0???00?01?010?0?0??00??
'Citipati_osmolskae' ??100000001010010 1?0?110100-00020111000----
0000100101?0010100001000-1101100101?10-1111000100?0-001200-

000?000000??010?01101111101?0??001?01010?1010112000000-0?001??1111110?0000021-
0201101111100-110-10-0-1010001100-0111------1----------2-----------
-??10-?110-????210010??1??????1?0?10?????????1??100??0??0?1?52????0???10?????1???0??2?01?
1????0??001001111200????????00?0??1100?10?1?????1?000?0???000??0?1?10?00????0?????????0
?22???0????0010000?????0-100????001?1???????0-01000?1010??0110???20?11102300102?000-
01??????2110010?0?10?????0???????????????0000??000????001??0???00?00?0?0?0?0??000-
'Conchoraptor_gracilis' ???0????0011100?0?00??1?????0?020??????-??????01????1?
???110??010?0-???????101?10-?1000001???0-?01??????0?000000??0?????????1??1??????????????0??
????0?2???1????00-??110????????0??21-0?01101??1100-110-1??0-?0?000??0???111-??-??1?-
-????-??2????-?????????????????????????0?????????????1????????????????????0??1??0????1?1??0?????
??1???????????????????????0????1???12?020000???00????0100??0?1????????00?0???0?????????????
????0?????????0?????0?????00100100????0-100?11?001?1???????0-
10000?1010??00?1???20???1????010??000-
0???????11?001??0?10?????0????????????????????0???????????01??0???00?00?0???0?0??0???
'Gigantoraptor_erlianensis' ???
??
????????????????????????????20-0001111011100-110-1??0-00?00?0-????????????????-???-??-2-----
-??101012?0000?0??
0??????????1???????????????????1?00??0100???110?110002?0??00-00000000?????00010-
10?000??
???????111110????10001???????????????????????????????????????1????????????????????0?0-
'Incisivosaurus_gauthieri' ??1000000101110100000100110-
000111110001???00001000011000100000?1000-1001200001000-101000010000-
00100100000000100101020110121011O1-
000001?0110???11?012001000-?0010?11??1100-??0?0?2011?01111111000-0-0000-0-
00?0?0110??0?101200?00?00??1?2001101?10--
10-???
??
???????????????????????????0?????????????????0???
???1?????????
'Microvenator_celer' ???
??
??????????????????????????????(1 2)0????11?1101100-????00-0-
0????????????????????0???????2??2????-?-
-??????1101?110???????0010?01?1?11102??10100000?00100100010??0?10?0?????????????1?11??00?
?2???????????????????????2002???????00?0??0?0??010110??02?00010000?000?0???????0????0?????
????????????2???0010?00001000-1001101001???1010000-
00?00100100?0101???20????????0??????????1??11?1012101000010O??000???11?001??00100000012
0001101???????????????????????????0-
'Oviraptor_philoceratops' ???100??0?11?????1????10????0?0?0??10??-???0??01????1??
??01000???00-????1?0101?????1?0?0??0??0-
001?0????0??0??00???10???1?1?1??1?1?0?1??????????????????????0????00-??????????????0?21-??0
11?1111100-110-10-0-?0?00???0????11-?????1?-
-??-?-??2????-??1???????????????0?????????????????0??????
?????????????????10???????????1111???????????????0???1???1??0????????00?0???0????0???1??????
??0?????????0??????0????00?0000?????0-
1???????0??1???0?10?????0???????
???????????????????????01????????????????????????????
'Rinchenia_mongoliensis' ???1?????011100?0?00??1?????????????0??-??????01????1?
???0100?010?0-
10?????101?0????0?00?????0-?0??????00?000?00????????1???1??1???????????0???????????0??????
?????????????????????21-0?011?11111?0-??0-10-0-?0?0?0??0???111-??-??1?-
-??-?-??2????-??0??????
????????????????1??????????????1??1???????????????0???1?0??0?0????????????0???0????????1???????
???????????????????????00?000??????102???111?01?1??
????????????????????0?????????0????????????????????????????????0????????????????????0??????
'Buitreraptor_gonzalezorum' ?????????0000??1?10???0?1???0??????????1???????0??????
?????00????0??????????????????1?10010??0-
001?0????0???0?10?001???????????????????????????????????????0?????????????????????????1?0?0
-?0????01010?????????????????????????????0??01-0020???00?00??00-

0??110?010-??201210110??12????010??????????0??1????0?????1?1??0?1??0???????1??01??10?2?01
??????1??2???????102?1???????1100???101101001???????????0??1??????1?110??????????????????
22????????????????0100??????????01?10?0????111?111?10????010????20112122??0112?0?1001????
???1??1???????????????????????1????????11?2000 1????01?0011?10??1-01001?0????1??
'Deinonychus_antirrhopus' ??000000200001?111101000?00-
0000000100010000000010000110??1000001010-0--1???100?110??10???10??0-
00??0???1001000?0??11031?110100?0???????????1?????????00??0????0?0?????????10??????0011?
00-?000??00-0-0001010?1001???1101010110000 02100?001000000?000100-0001111?10-
0010120001 1?0101101002010000000000 0010010101110 1100??????0?????00120010010?1001?1????1
?12?????1???21120000??110?0??1010?10?1?0???1000010001000?00?1110000110100100110011 1220
000212?0010000001?00-1??1100001?1??0?0000-1110012010??0101???001111222001021010-
010101111111110000 1000000 1011111?00101??10?0000020001101001 10011?10?100011101001010-
'Dromaeosaurus_albertensis' ????????2?0??1??1?1??100????0???0?010??10100??0????01
1???100??0101??????1???????0-
1210111???????0??0?????????000??01031???0?0??0001000001?00000100?0?0011?01010000-1110-
10-100000000011000-00000-00-0-000100-1110101111 01010?000110?000?00000000000?0100-
0???????????0??0????????????????????????????????????
??
???????????????????????????1??
??010-
'Linheraptor_exquisitus' ???000002000?1?1101?1?00?10-
00000001000????000?0100???0001000101010-???1110100?10-1?1000?100?0-00?200-
00001010?0?0?10????????0000?????????????????1?10101????????????????????????0??0??000-
000???00-0-0?010101????0??111???01?????02101?0?1000000??00100-???1111010-
1??00?0??11?11??1??????????????????????00???????10??????????10?0???000??001000?1101???1?1
110???????????????1?????01?????10?11???01?0??1?????10??????????????????0???00?????????2?????
???????1?0?????????????????????????????20????010????01??????????????????1???????????????????
00?101??111??10???10???0??2000110??011001??10?10?0?10???01010-
'Mahakala_omnogovae' ??
??????????????????????????????100?00???????????????????????????????????????0???????????????
????1001??1???????????10?0-
10??
???40?1??0???????1??01??10?2??????????1???????????
???????????????????????????????????1???10??????1110?0?????????????????????????????????????1
000-0???0-??11?1???????0-
11100???????????????????????????????????????1?11?111?0?0???0?0????????00??????0???00??0001?
???01?00??010?10-?1??0?0??010-
'Microraptor_gui' 100????01000???1??????00??0-
0??????????????00000???0?????1?0?0???????????1?0??????????0????0??0-
00????????????????????????????0??10?0??
?000??00-0-0?????????01???1????10???01?02?0??0?10000???00--
10-??????????????????0?111??1??????00??0?1????????????100???0?1?004????1????10??1?001??1--1-
01?1??????120100?1110?0?????????100???1111010?11???0????110??????000?110000000-
10?10?0????0?22???02??1001000000100101?0?111??1?1???????0-011?1?200--?010?1-
0011121?2??011011010?1??????11?111????10???0?0??????00??????0?0?0???0001???10110011?10?1
1?0??01?101010-
'Saurornitholestes_langstoni' ???????????????????????????????????????1??????0??????????1
??????????????????????????1?111??????????????????????????????1?????????????????????????????????
?????????0???????????????0????1??00-?0?????????0????0-???????????1????1???0???0??0?1000000?
000100-???1111010-
1?1??200011?11?1011?12??000???00???1??10100110110?41?1010100????1??0???1??2?01?1????1??
1?????????20??????????????????????0?????1???00?0?00?1?000??1?0?00?0-
100???110011022??002???00100000?10?10210110100??1?0010011100100???????????????0???1????
0?0??0???????????????????????????01011111???101??1?0110011000110 0101?0011?10?10?001?0?001
?10-
'Sinornithosaurus_millenii' ?000??001?000??1111?1000??0-
00001?11??01???0?00010????0000000?010?0-0--1??0100??1011101001 00?0-
001201101?0?0??0??0010??????????0???????????????0????????????????????????????????????0?101?00
0-00000-00-0-0?010100??0?0??1????101010000?10010?1?00000?000100-
0???????????????????????11?????1?????????????????????????????04?????0???????1??0??????2??????

???????01001111?20120000???10?0??1011?10?????????????0????????0????0000????1?????????1?22
???02?2?00?0000?????0-1???11??01?0???????0-
11??0?2011?????1???011121????011211010?1??????????1????????????????????????????????0??1001
???001?00???10??1?0???1?001?10-
'Tsaagan_mangas' ??00000020000?011010?100110-
000000010001???00000100?1?0001000101010-0--1100?????0-1110001100?0-001200-
000010?000?00103????0?000000100100100100010110101011 00???0000??10110-1000000?001?000-
00000-00-0-
0?01010111010111111?010101?0002101?0?1000000?000100-??????????????00?0?????110??01??????
???1?
??
??
?????????????????????????????
'Unenlagia_plus_Neuquenraptor' ???
??
??????????0??????????????????????1???
?0?????????????????????????????????????20?00101??????1??101??11011?0?0?1??0?0?????????????
??????????1??????????????????????110000?110?0010011021 1????????????????????????????????????
??????????????????00???01000-210011100111??010000-??111?20110101011-
00011110??0011????11010?01?1112101000?10??00?????11??01??????????????????????1???????0???
?11??1????????
'Velociraptor_mongoliensis' ??00000020000?0111101100110-
000000110001???000001000110000?00101010-0--1120100?10-111011010 0?0-
000?0???00?10?000?0010?1?1?0100?00??0010??001?00??1?01001?000??0?00-??11110-
100000010011000-00000-00-0-000100-?1?0?0111101010?110000?0011001000000000100-
0??111?010-???012000????10??0101???10?00????0?100101??1?01?0050?1010?0010001??010?1????
01?110??1?12010001100201200000??100000?1110010?1?0??01?00010??1100??0?1?10000????00100?
????0?2200002?2?0010000001000-11001110010 1?00?1000-01100020110?01011-
000111102200102 1010-
0111??1?11?11?000010000010?1???100???0????0000?20001???101?0011110010001111?001010-
'Epidexipteryx_hui' ?-????????????????????????????????????-??????00???0???
????0?001?00-????????????????0???00?0??0-
001??10????????00???0??10?2?0?00?
??1?10-00-10001??0?01?00???????010?011100?01??2120?101?0?1??00-
0??????0?????????????????12????000????100??????????00???0????04???0?0?0?2100???020?1??2??1
00????????1?0??????2?1????????00??0100?1?01?02?????0?0010?0?0??001????????0????????????0??
?22???0??????00001?010?0-
200011??????????0???????0??0?0??????????0?010102?????110????1?????????????????????0?????????
???0?????????1??0001???111000110????0?0???0????????
'Epidendrosaurus_ninchengensis' ???0?????????
???????????????????????????1???0????????01???
?????????????????????????????????0011111?0-00-100?10-
0?????????????????????????????1???1?1?0?1?????????????????????????????1???????0????1????????
?????0?????0?1??????????????????????????1???????????1??????????2???????????????100?1?00??2??
???????10?0??0?000????????0???1??01???0???22???0??00?000001???????????????????????????????
???????????????????????????????????1????????????????????0??0?????????0???????????????????????
?001100??00???????0???0??
'Scansoriopteryx_heilmanni'
0??0???10?001000-???1???????1?????????0??
????0??0?20??0
0??01?11-00-100?10-
0?0??1?0??0????1??0??0?0?100??????
????00???0????030???00?0?10?0??001??10-
0-??0???1??11??????????2??2000??1??????0?0?1?01??2???1?0?0?00???0000001?11000011010?1010
0?0?0?2200002100001000000?10?0-2001????01?1?00?????????????0?0-?????????(0
1)00??0?2?????1100???1???????2-???1????1?????????????????0?????????????????1???0??0011?00?00??
?00??101?0??
'Jinfengopteryx_elegans' ????????1?01???1?10?0?????????001???????????????0???????
????????????????????1??????????00????????0?0?????????????2??0????????????????????????????????
??100????????????????????????????0????0-?0??0-

0????????????1??????1?????0?000??0???????????1???????0?????????1???????????????????????????
???????????????????????0??????????10?????02????????0?011?????2?????????2?1????????1?????10
10???????????????????????????110?0?????????????????22?????????0?0????????????????????1????
???????????3????????????0??0??????????????
????????????????????????????????
'EK_troodontid' ??
??1????11??????????0????????0?????????
???????10????1????1??0?????01????????????????????????100-???0????1??????????????????????0?
??????1???????0-??
???1000010-?0?1?????
??1?22?00?2???0000??0???????????????????????????
???0011?00?1????0???1???10-
'Byronosaurus_jaffeei' ??00000011000001000 1?100110-
000001110001????00001000??0000???0????110--
1110111 0?0-?0???000?11?0??1???0?00011?100111????01
0-??110010101 11?????0?000-00000-0110-0?010???????????????101?001002201-002000120000--10-
0??10-?????????2??0??????210???1???????????????1??1???????0????????????????????0???2?????????
??
??1???????????????????????????????1??????
?????0?0?01??11????????????0???????????????????????????????????????1??
'Mei_long' ??00000011011?00?-??0?00?12100000?11000????00001
000??01--11?0?11?0-0--12?01110?0-1100?00??0???001100-
0???????10???11??????????0????????????????????110??????0???????????????0??0??????00-
0000??0110-0???????100????1????10?0?0?001201-0020???0???0--?0-0??110000-?--
101210110??1010??01?0?1?1000???00????00???00111040???1??0?1000?0001??2--0-
0?0?1?0???12?????1111 20120000?11000001101000000 10?111000110?0?????0????????????????10???
00???2??000?121001 0000001????????0--00??1?0011000-01100000???01001-
0101??10???0??20??10?10?01??1110110?0?11?000?????????011?0?100??0112000110?00100011???1
?1??10112101010-
'Saurornithoides_mongoliensis' ??1000??1100000??10?01?0????0???0?01???01000???0??????
???1???????????????110???????????0???????0-
00??0?????????????????????1?01????00?11?????????1??????????????0-
0??0-????????0-?11?0?000?0?100000??011??0?0?????????????1?????0?000??02101-
0010001000001010-
0???????????????????????1?????0?00??????0??000?00??0???000??0?0?10?000???001????00????????
??
??????0??01?1???0??111023001020000-
0???1111111011???01??????????1???0???1???1??
'Sinornithoides_youngi' ????????1?000???????????1???0?00???????????????00???????
???????01?????????????????0-??????????????0??????10?????????0????????????????????????????????
???????????0????????????????1????000??00-?000??0110-
0?????????????????????0?000??0?101???1??0100?10?010-????????10-???0??101????????????1???????
??????????????????????0??????0???10??1??01??1??0??1?0????1?12?????1???20120000???00?0??1??0
?01????????2??0?10?????????????10?0010-?0?1?????????22???02????0?0000???????????????001?1????
???0-
1???0?00??????01???00?1210??00?0210?0-?1???????1?011???????00?????????00??????0???0???????
????0100011?0?10????011?101010-
'Sinovenator_changii' ??1???0001010??0010?1100111000?11???000??????000???0
11????????0??????????????????0-???0?00???????01????????00??12??011??????????00010110001--
010??0110101111 0110010-?111001010 1001??1?0??0???000??011100?0??0-?1?0?0??????0101?00??0
?2?1?0?10?010??0?01?0-?????????10???0?210?10??01?????00???????0000?01??10000???0?1?40?1?
00???10??1??01??2??0?????????????1?????????20120000???10000?101?0?????????????????????????0?
??10?001????????????????????????????0000????????????111????1???????0-
11?00?2011??010????0011210??0011210010?10?01?11110110?0?11?00??11?111?0011???1000?10??0
001???001??????00?10???1?1?1???1??
'Troodon_formosus' ??0???001?0?0?010?011100?10-
0000?1??0??1??????00???0????????00????110--????100??0-?00?0001??00-?01??0-
0???0?0?1???01030??1?11??0?00110000???001??010?1000001???010-1110-10-
10?111??1?1??010?100??0110-
00?????????0????????101?00?00?201?0?1000220?001010-????111?10-

111??210110?01?10???1001001????????1??101??1??0?1050?1?10?0????011?0?0?20?0??1?????????2
??????????????????1?00000110??0?0?1?0?10???01?0????????0????000010-?0??0???00???220??????
?001?????????0-???????????????????????????0010??0001???0??11??23??1?21000-
0?0001?11120110?0?1???0?00110101??1????1?01000120001100?001?????100111?1?011?1??010-
'Zanabazar_junior' ??10000011000001010101001110000001110001????000010
00??1???1?????????????????111?????00?000100?0-
00120100?0000?0???????????????????001100?0????01??0??110001010-
0010-??10-???0-?1110???0?001000000-0110-000????????????1100?1010001102101-
0010001000001010-
0??40?1010?00???0??00???2-?0??????
???1?12??
??1??????0??????????????????????
???????????????????????????????20001?????1???????????-?1???????????
'Apsaravis_ukhaana' ???????0??
??0?????????????????1????????????????????????????????
?????????????????????????????????0??001010011100-0-0???????????????????????????????????????2-
---??????????????110-1?001?12110??1?11???00??0??????????????????????0???50?1210?0?210?10-
0200-??2?0110????????1?1001???3000-
000111000001101001100?00011001110001010000111110001????110?11-
0?1????????????????????11000-01010--0???1?1?1?000-10????3012--1---1-00000-00000-1-
22000??11?11112-2001?????1100????????????11????????11?????110?111?00-?000110?11010?001000-
'Archaeopteryx_lithographica' 1100001010010001010?1?00?00-
000100010001???00000100011001111000021?0-0--1100111110-1101000??0?0-000000-
010?00?000???0-
210??12?010?0?0???????????0??0?01110001???0???????1001011?00??10010000-?0000-00-0-0?00-
0-?1?0?00-0??001010001002001?212010000?00--10-
0??1???0?0-??????10??????0?????????0??????????????00?????0??04?????0???20?01??01??1???2?00?
0????1?111?00?1?102012-
00001100?0001111?110020??1?000010????000?011?10?00?0-?0??01???0?1?22?0?0??2??0000000???
00-010???1001?1??0????0-
01100?20?1????11???0011210000010?2101001??????11?0110?0??0?00??0??????0010?????00?00020
00110?11110011?00?00?0?010?0??010-
'Confuciusornis_sanctus' 11-000-01?000000----0?01?0--
00110011000????00000?0?01?11-111001-1?0-0???1?0????10-??00?0?10??0-
000?0????0?00?000??1?????????0??0?????????????????????1?????0??????????????????????0?10??00
0-?0011000-100?0100-?1?000??0???011---?--1----------2----??-----
-??????0???????????????????????0????0???0??????0??100?????1??05?????0???20?01??1-?0?????0101
10??????111001110?0?????????1???00?11110110020??11001110001?1000011?11?00?0-?01?0????0?1?
22?0?0?????000000?1?000-010????0?1?1????????0-??100?300---??1????0010-
1?????1?211011?11??????2-
20?1?????0??0??1??????00???????01??1??2110???111100??001?10?01??0?0??000-
'Rahonavis_ostromi' ??????????0????????????0??????????????????????????????
??
??
??????????????????????????????????????0120111000000?1001000010010?041?001?000??101200
10010020??????1?1112??????????????????1100000110??0????2?????????10001010000?????????????
?????????????????????00??????01000-21011110011100010000-0111002011??00111--
0011210000011121011010001112-
20110000100000101111110011100100100012000110100110011011110101110?001010-
'Sapeornis_chaoyangensis' ??0000000?010??1?1??0???????0??????1?00?????00001000
??????11-002?00-???12?0????10-11???0?100?0-0000?0-
0??10-?001001010-
00-0-0-00-0-?1?101??0??0010100?100?011-2120?2--------?0-
0??????0??????????2??????211?????0??????0???????????????????05???01??0?210-0??1-
0000??????0??????????????1112010-000?1100?0011011?11012?001?0011100?10000001111100010-
10?10111?0?1022?0?020010010000001000-11010-???1?1??010000-0?100?30110101101-01010-
10001011010011010?11112-
2?11??00100?0????????????1????????11??1001???11100010??0?10??10?0?0??000-
'Jeholornis_prima' ????????????0?????????????????????1???????????0???0???
?????????????????1?0????????????????????????????????????1????0????????????????????????????????

??????????????????100?????????0?????000-?0??????????????????????????????????????1------?-
-?1?0????---0-
0??????????????????????????????2??????????????????0?????????3????1????20?02??02?0?1??????0
??????????????11?2??????????0???0?1011?1(0
1)?0??????????10??001?0??1?111?0?????????????0?1?22?0???????000000???00-
110?10??0??1???1??1??????????????????????????????
?????????????????????????1?????????????????????????
'Yixianornis_grabaui' ???100??2?0?0???????????1??????????????????????0???????
?????-????????????????????????????????0-?0?????????????????1??????????????????????0???????????
???????????????????????????????0?0?0-10????00-????0?????1??0????????10100?101?--1--
12??100?0??--10-
011????110-???????0?????????????02?????00????????????0?????0??07???00????21?????1-?000?2???11
???-????1100001003000-
00011100?0?110110110???000100??10001000000010111?0??????????????0???22?????????01000??110
?0-000?0-?????1???????0-1?1???3012--?????-?1?01210000???20?01?010?????2-
201???????0??0???????????11????????1?1???????11??0010?????0???????0???0??
'Shishugounykus' ??
???
???
????????????????????????????????????00?0?1??????????????????????????30?1???????0000000?00?0??
??????????????????????????????????1??0?00000????00110110????????????????????????000110101000????
??022000?21?101000001??0??????????????????001??100??????????????????????????0000????????0-
0101011000?000000?10100010111111002?10010000?????????????0???????1??0??01???????0???

Table S1:

Measurements (in mm) of IVPP V23567. *, meaturement based on the preserved portion only; ?, measurement could not be taken due to damage; X, element not preserved.

		Left	Right
**DV ?**	Length		21.0
	Width		17.1
**DV ?**	length		21.5
	width		18.2
**SV 1**	proximodistal length		17.3
	dorsoventral height anterior margin		14.1
	dorsoventral height at midpoint		12.5
	mediolateral width		16.0
**SV 2**	proximodistal length		17.4
	dorsoventral height anterior margin		11.8
	dorsoventral height at midpoint		9.1
	mediolateral width		18.4
**SV 3**	proximodistal length		16.9
	dorsoventral height anterior margin		9.6
	dorsoventral height at midpoint		8.2
	mediolateral width		19.9
**SV 4**	proximodistal length		15.7
	dorsoventral height anterior margin		10.6
	dorsoventral height at midpoint		9.4
	dediolateral width		15.5
**Anterior CaV ?**	length		17.4
	anterior Dorsoventral Height		10.9
	medial Dorsoventral Height		9.4
	mediolateral width		12.8
**Middle CaV ?**	length		20.3
	anterior Dorsoventral Height		10.8
	medial Dorsoventral Height		7.8
	mediolateral width		11,9

**Middle CaV ?**	length	?	
	anterior Dorsoventral Height	11.3	
	medial Dorsoventral Height	10.5	
	mediolateral width	7.6	
**Posterior CaV ?**	length	26.9	
	anterior Dorsoventral Height	9.7	
	medial Dorsoventral Height	8.6	
	mediolateral width	7.3	
**Scapula**	length	X	*67.1
	width at acromion	X	?
**Humerus**	length	*49.3/~85.1	X
	diameter of shaft	9.3	X
**Ulna**	length	X	?
	diameter of shaft	X	5.5
**Radius**	length	X	?
	diameter of shaft	X	5.4
**Metacarpal II**	proximodistally length	X	21.7
	mediolateral width of proximal end	X	10.9
	mediolateral width of distal end	X	9.8
	dorsoventral high of proximolateral end	X	8.2
	width of the middle shaft	X	8.4
**Phalange II-1**	proximodistally length	X	45.6
	mediolateral width of proximal end	X	?
	mediolateral width of distal end	X	7.7
	width of the middle shaft	X	7.3
	max diameter of distal condyles	X	11.3
**Ungual II-2**	proximodistally length	X	*36.8
	mediolateral width of proximal end	X	7.3
	dorsoventral high of proximal end	X	15
	length of the flexor tubercle	X	10.5
**Metacarpal III**	proximodistally length	X	~42
	mediolateral width of proximal end	X	~8.9
	mediolateral width of distal end	X	8.2
	dorsoventral high of proximolateral end	X	~6.3
	width of the middle shaft	X	5.3
**Phalange III-1**	proximodistally length	X	31.6
	mediolateral width of proximal end	X	7.8
	mediolateral width of distal end	X	6.7
	width of the middle shaft	X	4.5
	max diameter of distal condyles	X	7.6
**Phalange III-2**	proximodistally length	X	41.7
	mediolateral width of proximal end	X	6
	mediolateral width of distal end	X	5.8
	width of the middle shaft	X	~3.6
	max diameter of distal condyles	X	8.5
**Ungual III-2**	proximodistally length	X	36.5
	mediolateral width of proximal end	X	7
	dorsoventral high of proximal end	X	14.6
	length of the flexor tubercle	X	?
**Phalange IV-1**	proximodistally length	X	13.7
	mediolateral width of proximal end	X	~4.7
	mediolateral width of distal end	X	5.2

	width of the middle shaft	X	3.2
	max diameter of distal condyles	X	5.1
**Phalange IV-2**	proximodistally length	X	13.5
	mediolateral width of proximal end	X	4.3
	mediolateral width of distal end	X	4.1
	width of the middle shaft	X	3.1
	max diameter of distal condyles	X	4.9
**Ungual IV-2**	proximodistally length	X	16.9
	mediolateral width of proximal end	X	4.6
	dorsoventral high of proximal end	X	7.9
	length of the flexor tubercle	X	4.4
**Ilium**	length of pubic peduncle	7.9	X
	width of pubic peduncle	8.8	X
	length of ischial peduncle	8.8	X
	width of ischial peduncle	9.1	X
	width of dorsal margin of acetabulum	26.2	X
**Ischium**	length of ischial shaft	31.9	X
	length of obturator flange	23.1	X
**Pubic**	width of pubic shaft	5.3	X
**Femur**	proximodistal length to lateral condyle	?	173.2
	proximodistal length to medial condyle	?	175.8
	~mediolateral width at femoral head	?	10.9
	~mediolateral width at distal condyles	26.0	26.6
	midshaft diameter (mediolateral)	?	10.4
	midshaft diameter (anteroposterior)	?	16.1
	greater tochanter anteroposterior thickness	?	10.9
**Tibia**	proximodistal length	222.7	?
	anteroposterior length at cnemial crest	27.8	25.6
	length of fibula crest	26.9	*21.9
	mediolateral width at distal condyles	26.0	25.8
**Fibula**	width of proximal end	21.3	21.2
	max diameter of shaft	5.5	?
**Metatarsal II**	width of distal articular surface	10.5	X
**Metatarsal III**	width of distal articular surface	10.0	X
**Pedal Phalanx III-1**	proximodistal length	31.5	X
	width of proximal articular surface	10.1	X
	width of distal articular surface	9.1	X
**Pedal Phalanx III-2**	proximodistal length	23.8	X
	width of proximal articular surface	8.5	X
	width of distal articular surface	7.4	X
**Pedal Phalanx IV-1**	proximodistal length	22.1	X
	width of proximal articular surface	9.3	X
	width of distal articular surface	9.8	X
**Pedal Phalanx III-2**	proximodistal length	19.4	X
	width of proximal articular surface	8.6	X
	width of distal articular surface	8.4	X
**Pedal Phalanx III-4**	proximodistal length	13.1	X
	width of proximal articular surface	5.9	X
	width of distal articular surface	5.5	X

Table S2:

Variations in the character-matrix between the three Shishugou alvarezsaurians

**Char.**	*Haplocheirus*	*Shishugounykus*	*Aorun*
15	1	?	0
20	1	?	0
27	1	?	0
48	1	?	0
53	1	?	0
55	0	?	2
56	1	?	0
58	1	?	0
73	0	?	1
74	0	?	1
78	1	?	0
82	2	?	0
109	1	?	0
117	0	?	1
184	1	?	0
192	1	?	0
195	1	?	0
204	1	?	0
206	0	?	1
211	1	?	0
214	0	?	1
215	2	?	1
228	1	?	2
229	2	?	0
242	1	?	0
258	0	?	1
268	0	?	1
271	0	?	1
277	1	?	0
279	1	?	0
291	1	?	0
305	1	?	0
314	0	1	?
325	?	0	1
378	0	1	?
394	0	?	1
400	1	?	0
412	0	0	1
413	0	?	1
414	1	1	0
419	1	0	1
421	1	0	1
425	1	?	0
431	0	0	1

432	1	0	0
433	1	0	0
442	1	0	0
444	0	0	1
445	0	0	1
469	0	1	?
484	0	?	1
505	0	1	?
536	1	1	0
542	0	1	0
543	?	1	0
549	1	0	0
560	0	?	1
576	1	0	0

***Shaded characters represent autapomorphies**

Table S3:

Additional morphological variations between the three Shishugou alvarezsaurians

1. **Orbital region**
   0. dorsally expanded
   1. laterally expanded

2. **Maxillary fenestra**
   0. occupying less than half of antorbital fossa
   1. occupying over half of antorbital fossa

3. **Serrations on maxillary teeth**
   0. apically directed and restricted to distal portion
   1. located posteriorly

4. **Dentary tooth**
   0. homodont
   1. heterodont with enlarged dentary tooth 4

5. **Alveolar margin of anterior end of dentary**
   0. flat
   1. dorsally convex

6. **Anterior portion of proximal end of scapula**
   0. bears a swell
   1. flat

7. **The notch between the internal tuberosity and the humeral head**
   0. shallow
   1. deep and narrow

8. **Depth / length of Mnual phalanx II-1**
   0. less than 0.2
   1. more than 0.2

9. **Mnual phalanx II-1**
   0. distally curved
   1. straight

10. **Medial and lateral surfaces of Mnual phalanx II-1 shaft**
    0. convex
    1. depressed

11. **Ventral surface of Mnual phalanx II-1**
    0. has a deeply furrowed ventral surface
    1. slightly furrowed

12. **Depth / Width of Metacarpal III**
    0. less than 1
    1. more than 1

13. **Metacarpal III**
    0. laterally bowed
    1. straight

14. **Manual phalanx III-2**
    0. shorter than metacarpal III

1. as long as metacarpal III

15. Compared to length of metacarpal III , Metacarpal IV
0. about half of metacarpal III
1. less than half of metacarpal III

16. Unguals III and IV
0. straight in lateral view
1. strongly recurved in lateral view

17. Flexor tubercles of Unguals III and IV
0. distally located
1. proximally located

18. The obturator process of ischium
0. distally located
1. proximally located

19. The groove for the ascending process of astragalus on tibia
0. slightly oblique
1. proximodistally oriented

**Char.**	*Haplocheirus*	*Shishugounykus*	*Aorun*
1*	0	?	1
2*	0	?	1
3*	1	?	0
4*	1	?	0
5*	1	?	0
6	1	0	?
7	1	0	?
8	1	0	0
9	0	1	0
10	1	0	1
11	0	1	1
12	1	0	0
13	0	1	0
14	0	1	1
15	0	1	1
16	0	1	1
17	0	1	1
18	1	0	?
19	?	0	1

*** characters represent diagnosis characters from [4,5]**

Figure S1:

Strict consensus of 202 MPTs, L = 3202 steps, a consistency index of 0.218 and a retention index of 0.602. Numbers below nodes indicate Bremer Support values.

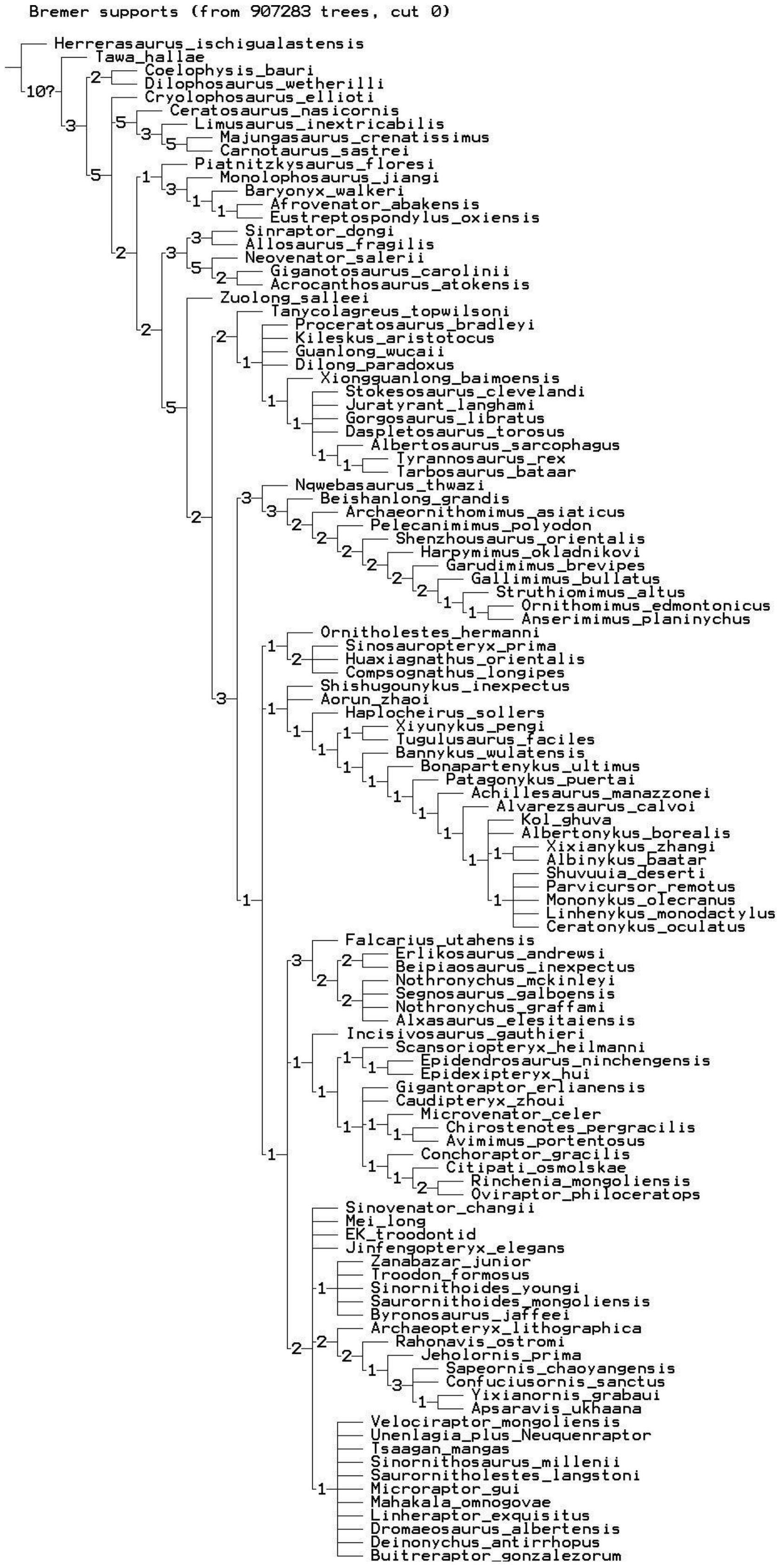

Diagnosis of *Shishugounykus inexpectus*:

Diagnosis. *Shishugounykus inexpectus* differs from all other alvarezsaurians in the following features (* marks the autapomorphies; we use the II-III-IV identity of manual digits): supratemporal fossa occupying large portion of frontal and with indistinct anterior border (sharp anterior border in early-branching alvarezsaurians such as *Haplocheirus sollers* and supratemporal fossa occupying a small portion of frontal in late-branching alvarezsaurians); scapula with hollow acromial process but without lateral concavities*; humeral internal tuberosity pinched distally*; metacarpal III straight in dorsal view (laterally bowed in most other alvarezsaurians including *Haplocheirus sollers*); ungula III-3 subequal in size to ungual II-2 (considerably smaller in most other alvarezsaurians including *Haplocheirus sollers*); iliac medial surface with step-wise transition from ischial peduncle to pubic peduncle*; distal end of metatarsal II asymmetrically ginglymoid*.

1. Supratemporal fossa occupying large portion of frontal and with indistinct anterior border.

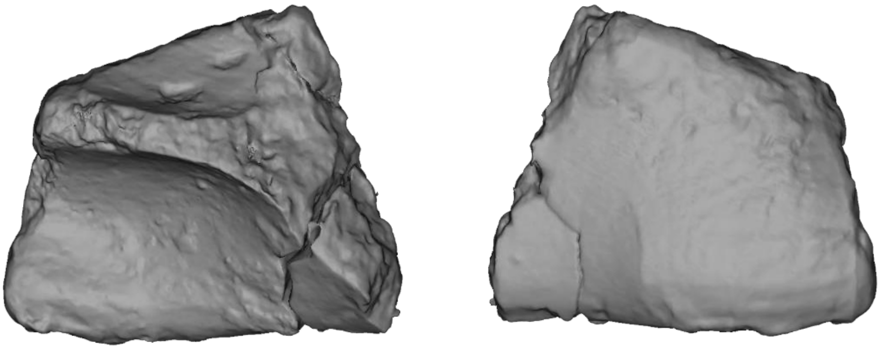

2. Scapula with hollow acromial process but without lateral concavities.

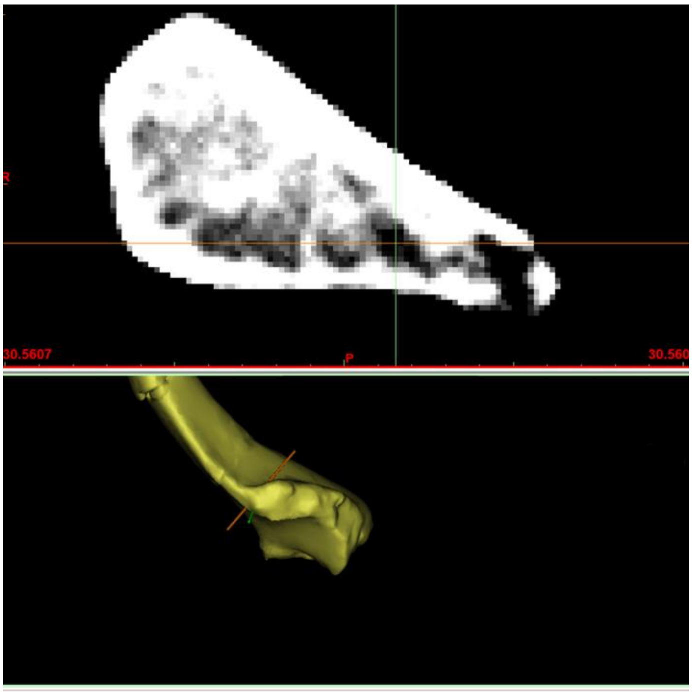

3. Humeral internal tuberosity pinched distally.

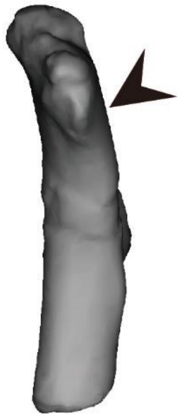

4. Metacarpal III straight in dorsal view.

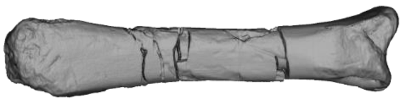

5. Ungula III-3 subequal in size to ungual II-2.

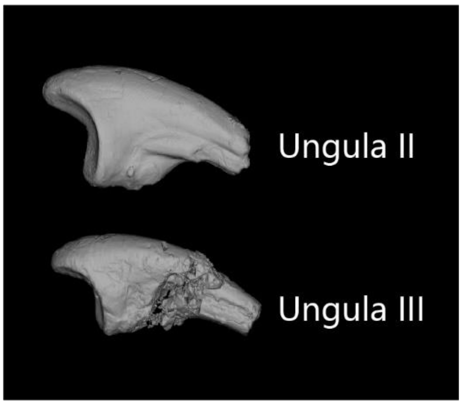

6. Iliac medial surface with step-wise transition from ischial peduncle to pubic peduncle.

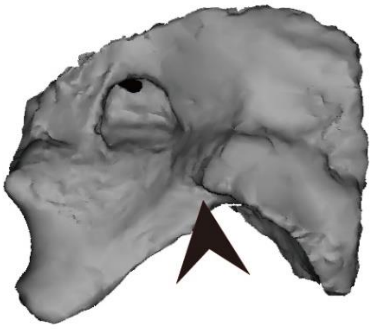

7. Distal end of metatarsal II asymmetrically ginglymoid.

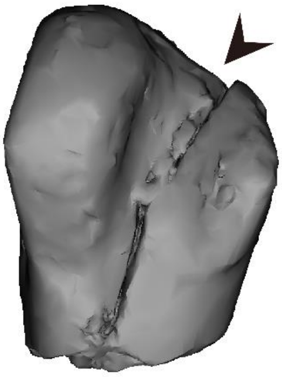

Figure abbreviations:

Vertebrae:
na: neural arch
t: tubercle
c: centrum
prz: prezygapophyses
poz: postzygapophyses
sp: neural spine
tp: transverse processes

Scapula:
sb: scapular blade
acr: acromial process

Humerus:
hh: humeral head
hit: humeral internal tuberosity
hdc: humeral deltopectoral crest

Maus:
mpII: metacarpal II
mpIII: metacarpal III
pII-1: phalanx II-1
pIII-1: phalanx III-1
pIII-2: phalanx III-2
pIV-1: phalanx IV-1
pIV-2: phalanx IV-2
uII: ungual II
uIII: ungual III
uIV: ungual IV

Ilium:
ipp: ilium pubic peduncle
iisp: ilium ischal peduncle
spacc: supraacetabular crest
f: fossa

Ischium:
ispp: ischium pubic peduncle
isip: ischium iliac pubic peduncle

lt: lateral tubercle
iss: ischium shaft

Femur:
fh: femoral head
fgt: femoral great trochanter
flt: femoral lesser trochanter
fpt: posterolateral trochanter
fft: femoral forth trochanter
pf: popliteal fossa
fmc：femoral medial condyle
fmc：femoral lateral condyle
fet: femoral ectocondylar tuber

Tibia:
tcc: tibial cnemial crest
tfc: tibial fibular condyle
tpc: tibial posterior condyle
tga: tibial grove on anterior surface

Pes:
mtII: metacarpal II
mtIII: metacarpal III
ppIII-1: pedal phalanx III-1
ppIII-2: pedal phalanx III-2
ppIV-1: pedal phalanx IV-1
ppIV-2: pedal phalanx IV-2
ppIV-4: pedal phalanx IV-4
